# From Donor‐Acceptor Ligands to Smart Coordination Polymers: Cyanothiazole‐Cu(I) Complexes for Multifunctional Electronic Devices

**DOI:** 10.1002/chem.202500215

**Published:** 2025-05-02

**Authors:** Karolina Gutmańska, Agnieszka Podborska, Tomasz Mazur, Andrzej Sławek, Ramesh Sivasamy, Alexey Maximenko, Łukasz Orzeł, Janusz Oszajca, Grażyna Stochel, Amarjith V. Dev, Chakkooth Vijayakumar, Konrad Szaciłowski, Anna Dołęga

**Affiliations:** ^1^ Chemical Faculty Department of Inorganic Chemistry Gdansk University of Technology Narutowicza 11/12 Gdańsk 80–233 Poland; ^2^ Academic Centre of Materials and Technology AGH University of Krakow Mickiewicza 30 Kraków 30–059 Poland; ^3^ National Synchrotron Radiation Centre SOLARIS Jagiellonian University Czerwone Maki 98 Kraków 30‑392 Poland; ^4^ Faculty of Chemistry Jagiellonian University in Krakow Gronostajowa 2 Kraków 30–387 Poland; ^5^ Chemical Sciences and Technology Division CSIR‐National Institute for Interdisciplinary Science and Technology (NIIST) Thiruvananthapuram 695 019 India; ^6^ Unconventional Computing Lab University of the West of England Bristol BS16 1QY UK

**Keywords:** copper(I) iodide, cyanothiazoles, fluorescence spectroscopy, X‐ray absorption spectroscopy, X‐ray diffraction

## Abstract

Cyanothiazoles, small and quite overlooked molecules, possess remarkable optical properties that can be fine‐tuned through coordination with transition metals. In this study, we investigate a promising application of cyanothiazoles, where their combination with copper(I) iodide forms a new class of complexes exhibiting outstanding optical properties. X‐ray crystallography of copper(I) iodide complexes with isomeric cyanothiazoles revealed key structural features, such as π─π stacking, hydrogen bonding, and rare halogen⋅⋅⋅chalcogen I⋅⋅⋅S interactions, enhancing stability and reactivity. Advanced spectroscopy and computational modeling allowed precise identification of spectral signatures in Fourier‐transform infrared (FTIR), nuclear magnetic resonance (NMR), and ultraviolet‐visible (UV–Vis) spectra. Fluorescence studies, along with X‐ray absorption near edge structure (XANES) synchrotron analyses, highlighted their unique thermal and electronic properties, providing a solid foundation for further research in the field.

## Graphical abstract

Cyanothiazoles, paired with copper(I) iodide, form complexes with luminescent properties. X‐ray crystallography revealed two types of coordination polymers, while spectroscopic, synchrotron, and electric measurements supported by density functional (DFT) calculations studies highlighted their unique electronic features, offering a foundation for future research.

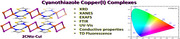

## 1 Introduction

Thiazole‐based compounds (**tzs**), characterized by the presence of an electron‐donating sulfur (‐S‐) and an electron‐accepting nitrogen (‐C═N‐) within their five‐membered aromatic ring, exhibit a unique combination of donor and acceptor properties.^[^ ^1^ ^]^ These compounds may find broad applications across various fields, including organic chemistry,^[^ ^2^ ^]^ medicine,^[^ ^3^ ^]^ and advanced material technologies.^[^ ^4^ ^]^ Their electron‐accepting ability is particularly valued, primarily due to the electron‐withdrawing nitrogen atom in the imine group (C═N) within the ring structure.^[^ ^4b^ ^]^ This feature significantly influences the interaction between the highest occupied molecular orbital (HOMO) and the lowest unoccupied molecular orbital (LUMO), leading to a reduction in the bandgap energy.^[^ ^5^ ^]^ **Tzs** are most commonly small molecules or conjugated polymers, which have been employed as n‐type or p‐type semiconductors in organic field‐effect transistors.^[^ ^6^ ^]^ They have demonstrated impressive electron and hole mobilities.^[^ ^2^, ^7^ ^]^ Small molecular compounds based on tzs can serve as efficient ambipolar materials, characterized by HOMO and LUMO energy levels of HOMO ≥ −5.6 eV and LUMO ∼ −3.15 eV, respectively.^[^ ^8^ ^]^ Alternatively, they can function as hole transport materials, where the required HOMO levels range from −5.61 to −5.28 eV. Moreover, extending the molecular structure by introducing thiazole units or substituting thiophene units with thiazole counterparts results not only in deeper HOMO/LUMO energy levels but also enforces an anti‐planar conformation of the polymer backbone.^[^ ^7b^ ^]^ These modifications can also induce a red shift in the absorption band and may slightly increase the Stokes shift compared to thiophene analogs.^[^ ^9^ ^]^ The compounds have been utilized as electron donors in solution‐processed organic photovoltaic devices and as electroluminescent or electron‐transporting materials in organic light‐emitting diodes.^[^ ^10^ ^]^ In sensor applications, thiazole compounds stand out for their ability to detect metal ions and other analytes at trace levels. This is achieved through interactions between the nitrogen atom or a substituent group and heavy metals, which induce changes in optical properties, such as color shifts or fluorescence alterations.^[^ ^11^ ^]^

**Scheme 1:**
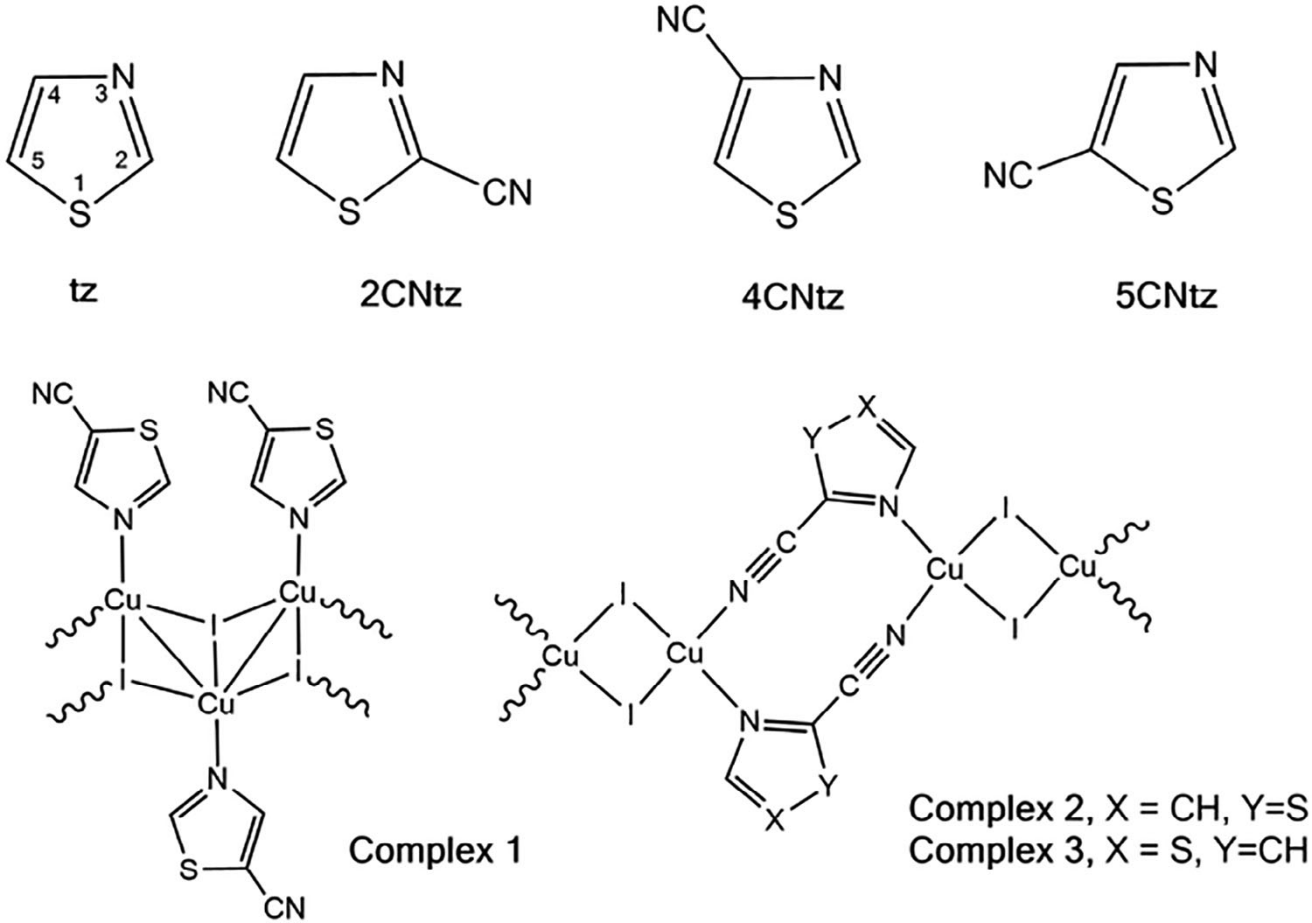
Thiazole (tz), cyanothiazoles (**CNtz**), and their CuI complexes **1**–**3** studied within this work.

The presence of ─C≡N group in the studied monocyanothiazoles and their complexes (Scheme 1) has a significant impact on the chemical and physical properties of the thiazole ring, making these compounds particularly attractive both from a theoretical research perspective and for potential applications. The nitrile group, being a strong deactivator of the aromatic system, has the ability to withdraw electrons from the thiazole ring. Through inductive and mesomeric effects, it reduces the electron density on the heteroatoms of the ring, such as sulfur and nitrogen. As a result, these heteroatoms become weaker electron donors, limiting their ability to bind metals inside the thiazole ring.^[^ ^12^ ^]^ The decrease in electron donation from the ring may simultaneously increase the preference of metals like Ag⁺ or Cu⁺ to coordinate with the nitrogen atom of the nitrile group, which we observed within the studied complexes (Scheme 1). Exploiting these simple relationships, we aimed to design a novel, previously unexplored class of hybrid materials based on copper iodide and cyanothiazole isomers with unusual optical properties.

The main motivation to study the structure and properties of new cyanothiazole‐based complexes is the quest for new materials with unprecedented electrical and optical properties with potential application in optoelectronics and neuromorphic computing. Resistive switching, a crucial process in memristive devices, provides both memory and plasticity features, and may result from a series of phenomena. The resistive switching process can be volatile or non‐volatile. For computational applications, volatile switching with low ON/OFF ratio is acceptable (and for some applications, like reservoir computing, even desired),^[^ ^13^ ^]^ but for memory applications,^[^ ^14^ ^]^ as well as for memristive cryptography,^[^ ^15^ ^]^ the non‐volatile switching is desired.^[^ ^16^ ^]^ The most common volatile resistive switching is usually based on one of the following mechanisms: formation of metastable conductive filament, Mott transition in semiconducting materials, ionic diffusion, or capacitive effects.^[^ ^17^ ^]^ There is a huge amount of data on these systems, and intense studies on the volatile (or semi‐volatile) memristors are being conducted, both for bulk and for interfacial processes. In the interfacial mechanism, the change in conductivity is related to the change of energy barrier at the interface between metallic contacts and the semiconducting layer (mostly hybrid organic/inorganic material, like lead iodide perovskite). Formation of the Schottky barrier and the reversible population of metal‐induced gap states are considered as the main phenomena responsible for switching.^[^ ^18^ ^]^ The performance of Schottky barrier‐based devices can be easily tuned by appropriate molecular engineering of the active layer as well as the selection of metal contacts.^[^ ^18^, ^19^ ^]^ On the other hand, these devices offer rather low ON/OFF ratio and their current/voltage characteristics are highly nonlinear, and the OFF current is significantly high.^[^ ^20^ ^]^ All these features make them difficult to use for memory applications, however, their significant role in unconventional computing is unquestionable.

The non‐volatile memristive devices operate on the basis of a couple of phenomena: ferroelectric ordering, spin polarization, phase change, and formation of conductive filaments.^[^ ^17a^ ^]^ The first three mechanisms were well explored from the material engineering point of view. Whereas the filamentary mechanism, despite its simplicity and robustness, has not been paid enough attention. There is a huge file of reports on filamentary memristors, but most of them were fabricated by serendipitous chance, and in our opinion, the materials were not optimized for the best performance. In the filamentary switching, the electric field across the structure results in the formation of conductive pathways due to migration and concentration of dopants, and thus the local increase of conductivity. The same can happen upon redox reaction — resulting in the formation of metallic filaments. The latter is accompanied by the oxidation of metallic contacts and movements of the metal ions within the solid‐state matrix (usually polymer or ceramic material).

Filamentary devices, in contrast to interfacial ones (semivolatile memristors), offer slower switching, require forming prior to use, but at the same time provide much higher memory retention times and very high ON/OFF ratios. The most significant drawback of filamentary memristors is the necessity of the forming process–multiple voltage scans used to predefine random conductive pathways. It is expected that metal complexes with redox‐active metal ions will contribute to the formation of conductive filaments and increase the ON/OFF ratio of memristors, but at the same time decrease the switching speed.^[^ ^16^, ^21^ ^]^ Furthermore, so far there were no reports on material‐related optimization of their performance, and there is only a single report related to coordination‐controlled formation of conductive filaments in silver‐doped organic materials. The only prototypical system reported so far is based on poly(methyl metacrylate) thin films doped with azobenzene.^[^ ^22^ ^]^

## 2 Results and Discussion

### 2.1 Syntheses and Molecular Structures of CuI Complexes with Cyanothiazoles (**CNtz**)

**Scheme 2:**
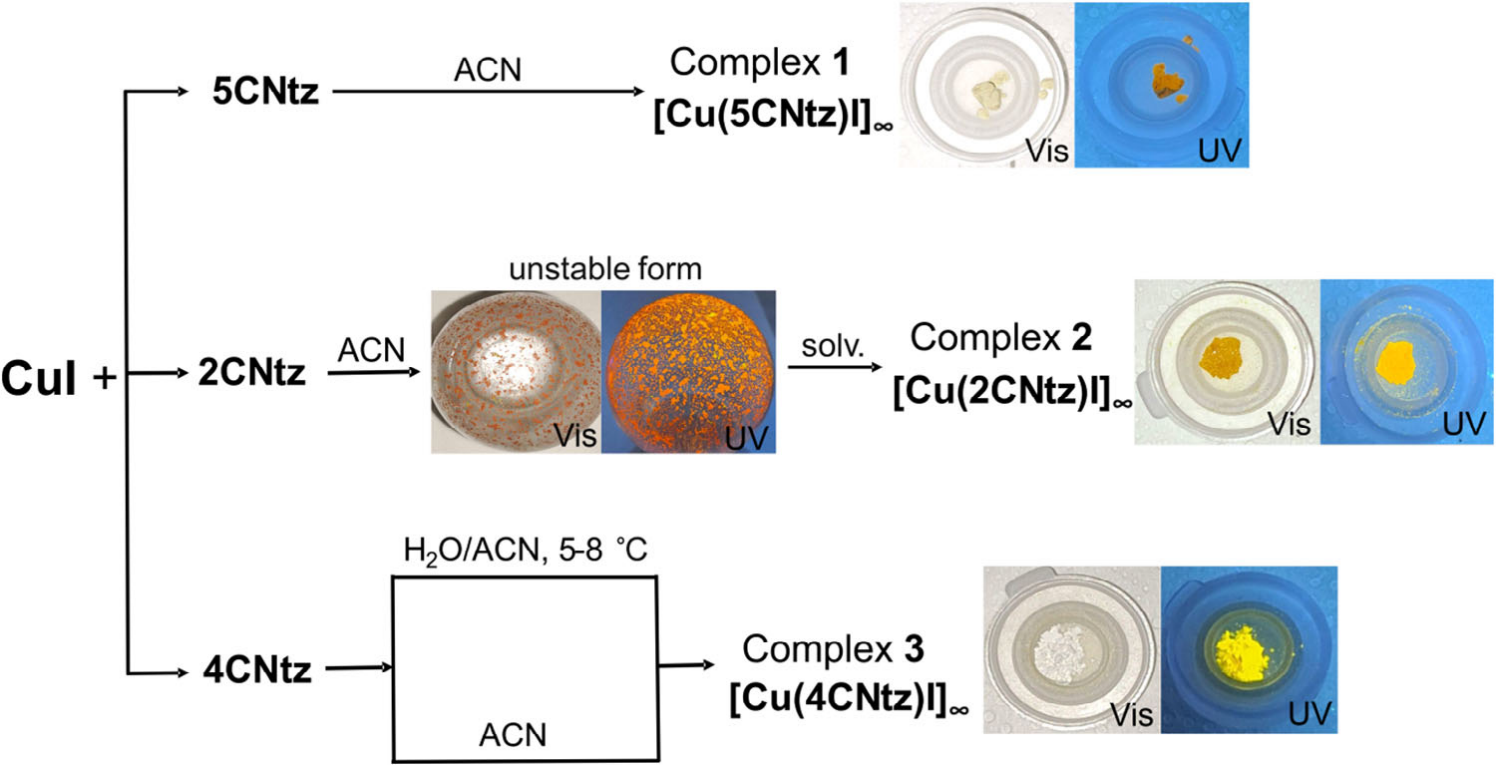
Reactions of CuI with **CNtz** isomers. The formation of the unstable form of **2** (polymorph?) is indicated. The details are described in the Experimental Section.

Due to the remarkable ability of CuI to form various polymorphic modifications, which may be thermodynamically unstable,^[^ ^23^ ^]^ the synthesis of well‐defined complexes of cyanothiazole with copper(I) iodide presents a certain research challenge. Fortunately, in the reaction between CuI and **5CNtz**, only one stable polymeric complex, **1**, was successfully obtained. For **2CNtz**, regardless of the molar ratio of reagents or reaction parameters, an intermediate compound is initially produced, which quickly transforms into a stable form referred to as complex **2**. It is suggested that this intermediate is a so‐called “disappearing or vanishing polymorph”, characterized by an extremely short lifetime, rendering detailed experimental investigations virtually impossible.^[^ ^24^ ^]^ In the case of complex **3**, depending on the synthesis method employed, two different forms can be obtained, which can transform into one another. The discussed synthetic pathways are illustrated in Scheme 2. The processes highlight the complexity of systems based on copper(I) iodide, while also underscoring their research potential. The further studies focused exclusively on stable polymorphic forms, which may find application in future technological solutions.

**Figure 1:**
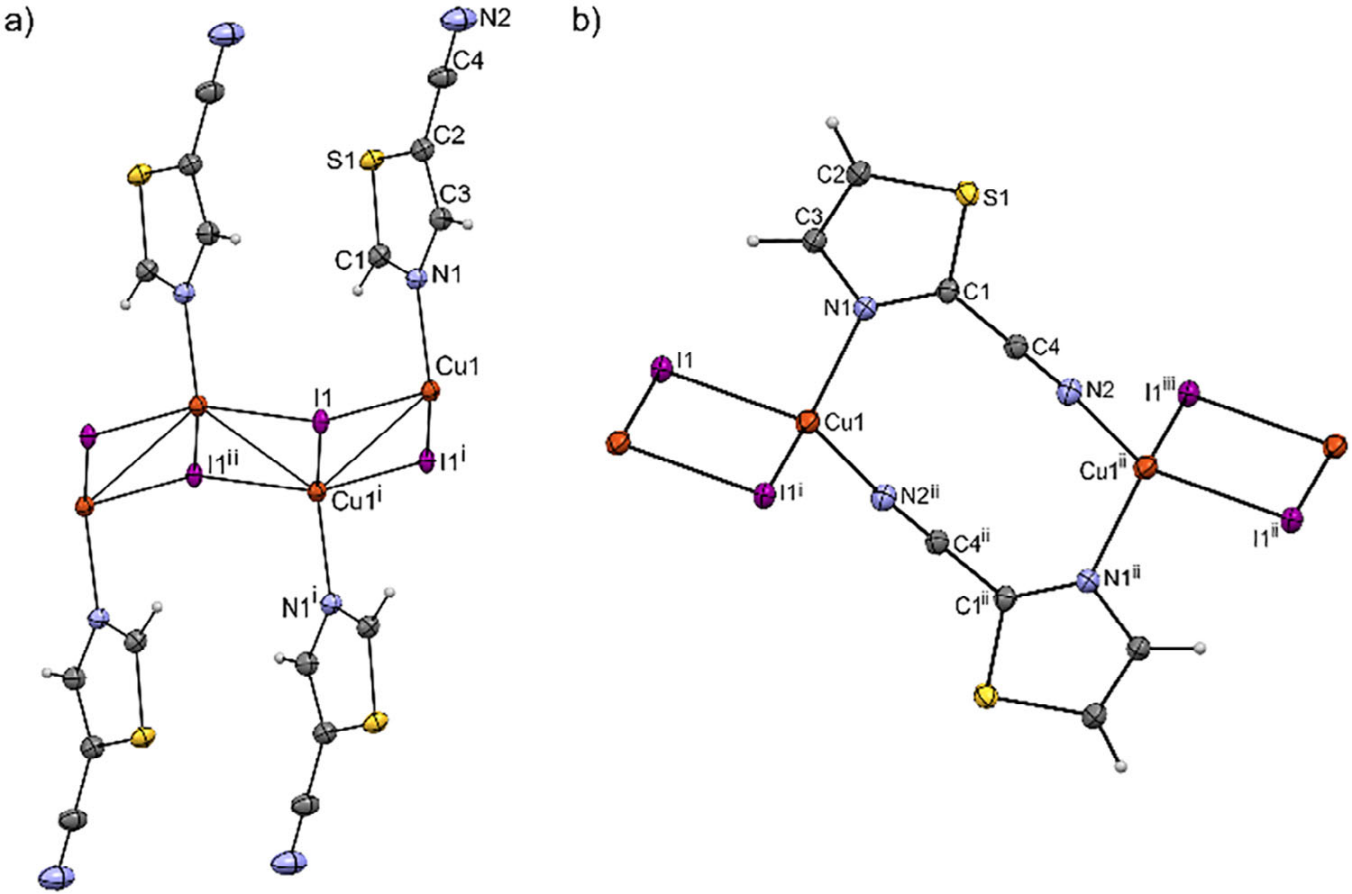
Molecular structures of CuI complexes with cyanothiazole isomers **5CNtz** and **2CNtz**. The fragments of the polymeric ribbons are depicted: a) complex **1**, symmetry operations: ^i^: 1‐x, 1‐y, ‐z; ^ii^:‐x, 1‐y, ‐z; b) complex **2**; symmetry operations: ^i^:2‐x, 2‐y, 2‐z; ^ii^:2‐x, 1‐y, 1‐z; ^iii^: x, ‐1+y, ‐1+z. Thermal ellipsoids at 50% probability level.

The crystal and refinement data for **1** – **3** are collected in Table S1. The details of the molecular geometry can be found in Tables S2–S5. The symmetrically independent unit of each of the complexes **1** – **3** contains CuI fragment and one molecule of cyanothiazole. The basic synthons form 1D coordination polymers, which crystallize in the triclinic system, space group *P*‐1. Copper(I) cations in each compound have a coordination number equal to 4 (C.N.═4) and exhibit a distorted tetrahedral geometry. This is due to the formation of Cu₂I₂ rings by the bridging iodide anions, which distort the local geometry around the metal atoms. The calculated geometrical parameters τ_4_/τ_4_′ confirm a slightly distorted tetrahedral geometry, with values of −0.89/0.89 for **1**, 0.90/0.98 for **2**, and −0.94/0.90 for **3**, respectively.^[^ ^25^ ^]^ Another common feature of complexes **1**–**3** is the center of symmetry, which is located in the middle of the resulting Cu_2_I_2_ ring. Among the three compounds, however, there are two distinct types of polymeric systems. Compound **1** features Cu₂I₂ rings sharing a common edge, forming a zigzag (Cu₂I₂)_∞_ ladder decorated with monodentate **5CNtz** ligands linked to copper(I) via the nitrogen atom (Figure 1a). In contrast, compounds **2** and **3** consist of discrete Cu₂I₂ rings connected into one‐dimensional polymers through two bridging 2CNtz or 4CNtz ligands, respectively, as illustrated by complex **2** in Figure 1b. Both **2** and **3** crystallize with similar parameters of a unit cell in the same crystal system (Table S1).

**Figure 2:**
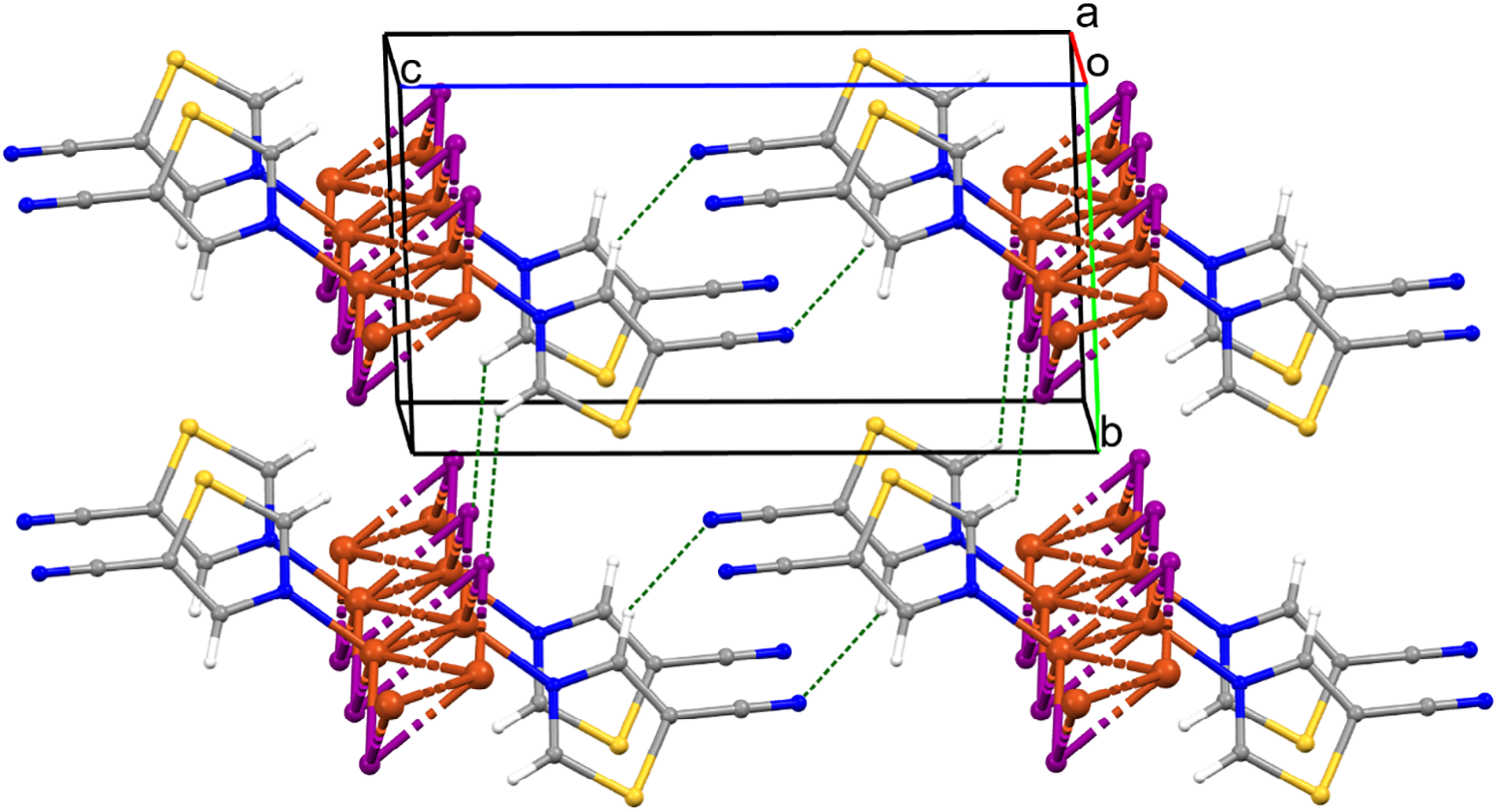
Crystal packing of compound **1** viewed along axis *a*; C1–H1···I1 and C3–H3···N1 interactions are indicated as green dashed lines.

The most distinct compound among **1**–**3** is compound **1**, in which the coordination of copper cations by the nitrile group is not observed. This compound forms a typical copper(I) iodide chain in the “staircase” or “ribbon” form, consisting of copper and iodine atoms with the general formula [Cu_x_I_x_]_n_.^[^ ^26^ ^]^ Within the chain, cuprophilic interactions are observed, with a distance of approximately 2.75 Å. These Cu–‐Cu interactions cause the chain to deform, adopting a staircase shape. The **5CNtz** molecules coordinate to copper atoms through the nitrogen atom of the thiazole ring, positioning themselves perpendicularly to the [Cu_x_I_x_]_n_ chain at a distance of Cu1–‐N1 2.050(4) Å (Figure 1a). The interchain contacts in compound **1** are illustrated in Figure 2. The stabilization of the crystal structure is based on several types of weak interactions. We indicate the presence of weak hydrogen bonds; the contacts that do not exceed the sum of van der Waals radii are C3–H3···N2_(1‐x,1‐y,1‐z)_ with the length of 3.413(7) Å, and C1–H1···I1_(1+x, ‐1+y,z)_ with the length of 3.749(4) Å (see also Table 5S). Up to 0.2 Å exceeding the sum of the van der Waals radii, one can also find chalcogen···halogen contacts between sulfur and iodine atoms: S1···I1_(1+x,‐1+y,z)_ of 3.968(1) Å and S1···I1_(x,‐1+y,z)_ of 3.979(1) Å as well as chalcogen···pnictogen proximity N2···S1_(2‐x,‐y,1‐z)_ of 3.541(6) Å. Interestingly, no interchain π···π stacking interactions were observed in this system, which typically dominate the stabilization of thiazole‐based molecules.^[^ ^4b^ ^]^ The bond lengths, angles, and short intermolecular contacts for complexes **1**–**3** are collected in Tables S2–S5.

In compounds **2** and **3,** a characteristic element is the presence of the dimeric unit Cu_2_I_2_, which is present in about 57% of all described and synthesized copper(I) complexes.^[^ ^26a^ ^]^ In both compounds, the Cu1 atom is coordinated by an N1 atom derived from the thiazole ring, an N2 atom derived from the nitrile group, and two iodine ions (Figure 1b). The second characteristic feature is the formation of a deformed ring including Cu atoms and CNtz isomers, which is practically identical for **2** and **3** (see Figure S1 for the overlay of the molecular structures of **2** and **3**). The Cu···Cu distances within these two rings: Cu_2_I_2_/Cu_2_(CNtz)_2_ are 3.1733(5)/5.3415(7) Å in compound **2** and 3.083(3)/5.356(3) Å in compound **3**. Due to the relatively high electron density at the iodine atoms, this ring is aligned perpendicularly to the Cu_2_I_2_ cluster. In addition, in both compound **2** and **3** the Cu1–N2_(C≡N)_ bond with the nitrile group (1.943(2)Å in **2**/1.949 Å in **3**) is shorter than for Cu–N1_(Tz ring)_ (2.064(2) Å in **2**/2.072(8) Å in **3**), which suggests large delocalization of the charge in cyanothiazoles and their complexes with copper(I). The phenomenon will be further discussed in the paragraph devoted to DFT calculations.

**Figure 3:**
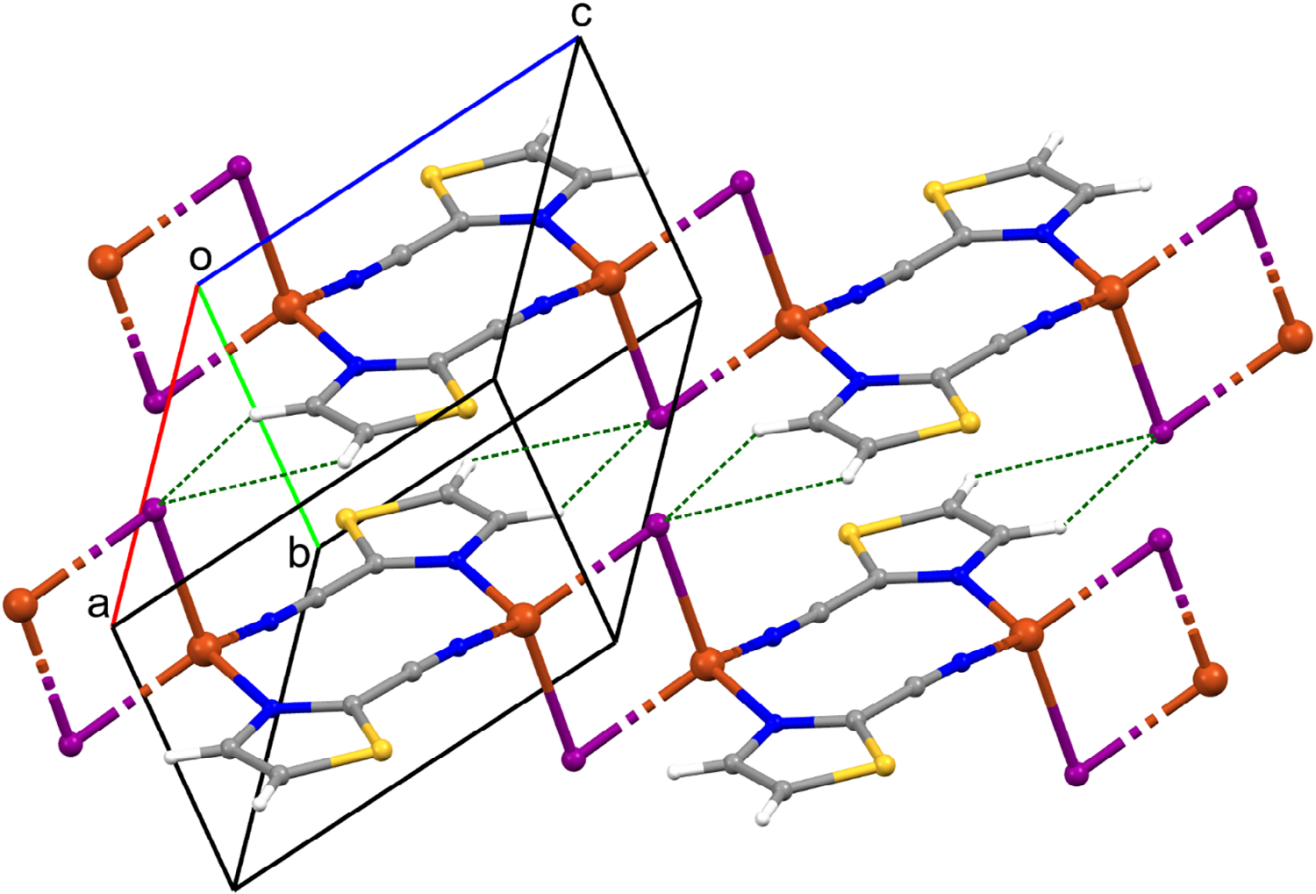
Crystal packing of compound **2** indicating C─H···I interactions as green dashed lines.

The chains of compounds **2** and **3** adopt similar mutual orientations with the set of analogous intermolecular interactions illustrated in Figure 3 for complex **2**. Important stabilizing interactions between the chains in **2** and **3** are face‐to‐face π···π stacking interactions between the thiazole rings. The Cg_(Tz ring)_···Cg_(Tz ring)_ distances are 3.783 Å for compound **2** and 3.882 Å for **3**, where Cg is the centroid of the thiazole ring (Table S4). In Figure 3 we indicate two types of C─H···I in compound **2**: C2–H2···I1_(‐1+x,y,z)_ of 3.776(2) Å and C3–H3···I1_(‐1+x,y,z)_ of 3.793(2) Å. In compound **3** the hydrogen bond lengths are C1–H1···I1_(1‐x,1‐y,2‐z)_ of 3.71(2) Å and C3–H3···I1_(1‐x,1‐y,1‐z)_ of 3.78(2) Å (Table S5).

Similar to **1** we have found relatively short chalcogen···halogen contacts between sulfur and iodine atoms in **2** and **3**. Halogen–chalcogen I···S interactions are rather uncommon and are not widely known and described in the literature.^[^ ^27^ ^]^ These I···S distances that do not exceed the sum of van der Waals radii are: S1···I1_(‐1+x,‐1+y,‐1+z)_ 3.7143(7) Å for compound **2**, and S1···I1_(‐1+x,‐1+y,‐1+z)_/S1···I1_(1‐x,2‐y,1‐z)_ 3.632(2) Å/3.665(4) Å for compound **3**.

**Figure 4:**
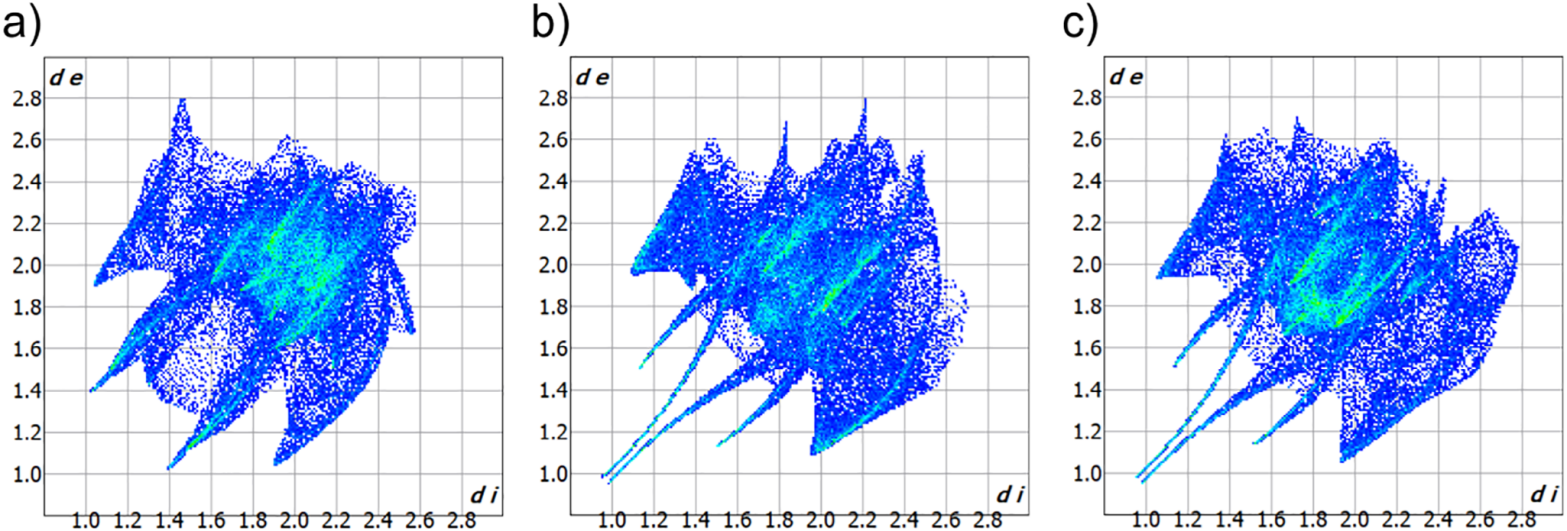
The all‐interactions fingerprint plot for compound: a) **1**, b) **2** and c) **3**. Analysis performed with the use of CrystalExplorer.^[^ ^28^ ^].^

**Figure 5:**
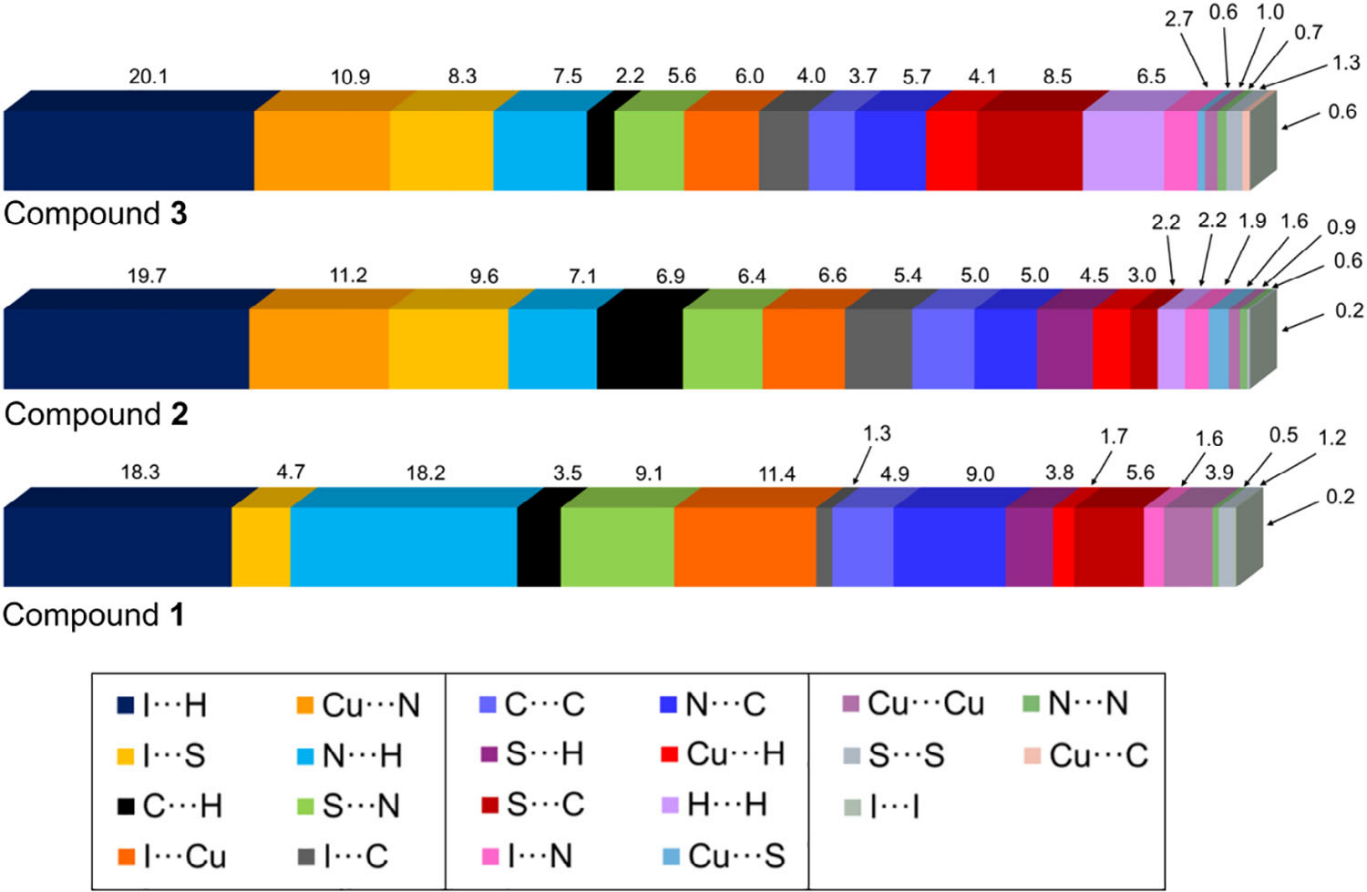
The contributions of various types of contacts on the Hirshfeld surfaces of the complexes **1** – **3**. Analysis performed with the use of CrystalExplorer.^[^ ^28^ ^].^

In order to confirm and clarify the nature of the bonds and interactions in the obtained coordination compounds, a Hirshfeld surface (HS) analysis was performed, and fingerprint plots were generated.^[^ ^28^ ^]^ These plots provide a two‐dimensional mapping of the point distribution on the surface as a function of the distance parameters d_i_ and d_e_. All fingerprint plots for the presented compounds, which illustrate all interactions within the polymer, are characterized by a spread of points covering the ranges of d_i_ and d_e_ from 0.9 to 2.8 Å, respectively. The results of the Hirshfeld analysis are presented in Figures 4 and 5. Hirshfeld surfaces calculated for selected fragments of the polymeric chains are presented in Figure S2 while Table S6 collects the detailed fingerplots for the specific contacts on these HS.

The dominant interactions with the greatest impact on structural stabilization are C‐H⋅⋅⋅I which constitute approximately 20% of all interactions (percentage of the HS). The fingerprint plots exhibit a subtle shape that is shifted towards longer distances, with the d_i_ and d_e_ values averaging between 1.8 and 2.8 Å. This suggests that long‐range interactions play a crucial role in the studied compounds. For compound **1**, C─H···N interactions are also significant, occurring at a comparable proportion of 18.2%. These interactions are characterized by sharp, distinct shapes, contrasting with the more diffuse and subtle shapes observed in compounds **2** and **3**. The previously discussed atypical I···S interactions are marked by a sharp, narrow, and slender peak in the upper region of the fingerprint plot. These interactions are most prevalent in compounds **2** (9.6%) and **3** (8.3%), while they are less pronounced in compound **1** (4.7%). Additionally, C─H···π (C···H contacts) and π···π (C···C, N···C, S···C contacts) interactions significantly contribute to the stabilization of the structures, collectively accounting for approximately 20% of the total interactions in each compound. Here, some differences between **2** and **3** are encountered (Figure 5 and Table S6).

### 2.2 Fourier‐transform infrared attenuated total reflectance FTIR ATR Spectroscopy

The analysis of FTIR ATR spectra of the obtained compounds enabled the identification of characteristic band shifts, allowing for a detailed examination of copper(I) coordination polymers with cyanothiazoles. Due to their structural similarity, the three polymers will be discussed collectively, while the FTIR spectra are presented in Figures S3–S20. In all FTIR spectra, a band shift of approximately +20 cm⁻¹ relative to the substrate was observed, attributed to symmetric and asymmetric stretching vibrations of C─H bonds within the thiazole ring.^[^ ^29^ ^]^ Noticeable differences in band positions arise from the simplicity of the organic molecule, where even minor modifications within the thiazole ring result in shifts or splitting of these bands in the spectra (Figures S4, S5, S10, S11, S16, and S17). For the nitrile group, the observed shift is minimal and insufficient to unequivocally confirm copper(I) coordination via the ‐C≡N group. In contrast, in complexes with Ag⁺ ions, shifts in the nitrile group bands are significantly more pronounced.^[^ ^30^ ^]^ The greater shifts in the FTIR bands for the nitrile group (‐C≡N) coordinating to Ag⁺ ions compared to Cu⁺ ions arise from fundamental differences in their chemical and physical properties. Ag⁺ ions are characterized by a larger ionic radius and higher polarizability, resulting in more diffuse and pronounced interactions with the ligand, which significantly alter the electronic distribution within the nitrile group. In the case of Cu⁺, the smaller ionic radius and a stronger covalent component in the bond with the nitrile group limit its influence on the ligand's electronic structure. Additionally, Cu⁺ exhibits the ability for back‐donation–‐partial electron transfer from the nitrile group to the metal's d orbitals–‐which stabilizes the complex and reduces the electron deficiency in the nitrile group. This results in less significant band shifts compared to Ag⁺, for which back‐donation effects are negligible.^[^ ^31^ ^]^ Further analysis of FTIR spectra of Cu(I) compounds with nitriles revealed that in complexes **2** and **3** the intensity of the nitrile stretching mode is suppressed by coordination to the Cu⁺ cations. This phenomenon can be attributed to the changes in the charge distribution on the ligand due to coordination to the metal ion.

Differences between the compounds become more apparent in the region around 1500 cm⁻¹. For compound **1**, a shift from 1493 cm⁻¹ to 1505 cm⁻¹ of the band attributed to stretching vibrations of the C═N group within the thiazole ring was observed.^[^ ^32^ ^]^ In compounds **2** and **3**, such a shift does not occur, which can be explained by differences in molecular structure between the complexes. For compounds **2** and **3**, only minor shifts in bands corresponding to C═C stretching vibrations are observed in the range of 1464–1473 cm⁻¹, characteristic of skeletal vibrations of the thiazole ring.^[^ ^33^ ^]^ Aromatic C–N bond vibrations appear in the range of 1300–1350 cm⁻¹.^[^ ^34^ ^]^ In compound **1**, the shifts are minimal, whereas for compounds **2** and **3**, they are more pronounced, with values of 1317–1309 cm^−1^.

In spectroscopic studies, bands attributed to C─H bending vibrations were observed in the range of 1130–1113 cm⁻¹.^[^ ^35^ ^]^ The C─S stretching vibrations typically appear in lower frequency regions, below 700 cm⁻¹,^[^ ^34^, ^36^ ^]^ and for the analyzed compounds these bands occur in the range of 536–507 cm⁻¹.

The analysis of bands in the fingerprint region allows for the identification of common features among the studied compounds. This is evident in the shifts of bands characteristic of the deformational vibrations of the thiazole ring and the N═C─H group in the 900–700 cm⁻¹ region. Significant shifts in these bands are a direct effect of copper coordination to the aromatic nitrogen atom.

### 2.3 Optical and Electronic Properties—Experimental and Theoretical Studies

Diffuse reflectance spectra of the studied complexes dispersed in barium sulfate matrix have been analysed according to Tauc approach. Diffuse reflectance spectra were converted to the Kubelka‐Munk function, defined as follows (Equation 1):

(1) $$F\left(R\right)=\frac{{\left(1-R\right)}^{2}}{2R}\;$$

where *R* is the reflectance. For powder samples dispersed in scattering media, it is commonly assumed that F(*R*) is proportional to the absorption coefficient α.^[^ ^37^ ^]^ Then the Tauc function has been applied to fit the linear fragment of the spectrum (Equation 2):^[^ ^38^ ^]^

(2) $${\left(\alpha \cdot hv\right)}^{\frac{1}{r}}=A\left(hv-E_{g}\right)$$

where *A* is the proportionality constant independent of the photon energy, *h* is the Planck constant, *v* is the photon frequency, *E_g_* is the bandgap, *α* is the absorption coefficient, and *r* is the exponent describing the nature of the bandgap: *r* = ½ for direct and *r* = 2 for indirect transitions, respectively.^[^ ^38^ ^]^ According to DFT models, the bandgap of Complex **2** is direct, and the bandgaps of complexes **1** (**5CNtz**‐CuI) and **3** (**4CNtz**‐CuI) are indirect, however, the energy dispersion of band edges is very low, indicating low mobility resulting from insufficient electron delocalization in the solid. Therefore, as in the case of amorphous (or almost amorphous) materials or molecular crystals, it can be safely assumed that *r* = 1.^[^ ^39^ ^]^ The same approximation is commonly used for molecular crystalline materials as well as for ionic crystals with only week covalent interaction between ionic species.^[^ ^40^ ^]^ Despite the fact that DFT models predict direct and indirect bandgaps, the *r* = 1 case provides the best fit for the spectra, which is consistent with relatively weak intermolecular interactions in covalent crystals. Therefore, the final equation that allows the determination of the bandgap is derived as follows (Equation 3):

(3) $$F\left(R\right)\cdot hv=A\left(hv-E_{g}\right)$$

**Figure 6:**
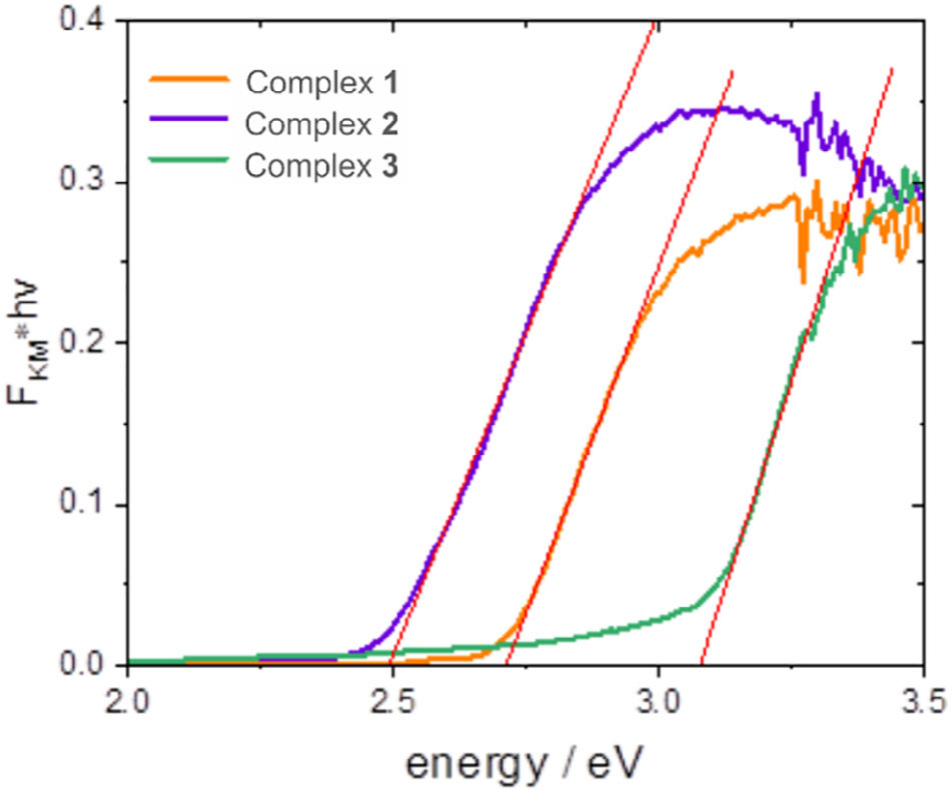
Diffuse reflectance spectra of three cyanothiazole‐copper iodide complexes in the form of Kubelka–Munk function.

Spectra, along with corresponding linear fits, are shown in Figure 6. Thus, evaluated bandgap energies amount to 2.49, 3.06 and 2.71 eV for 2‐, 4‐, and 5‐cyanothiazole complexes **2**, **3** and **1**, respectively. These data consistently indicate a decrease of bandgap energy as compared to the parent CuI semiconductor. It is justified by changes in the electronic composition bands, mainly the conduction band. The electron acceptor character of the cyanothiazole ligand makes the energy of the conduction band significantly lower than in the parent material. Moreover, both 2‐ and 5‐cyanothiazole complexes show distinct absorption maxima at 3.10 and 3.25 eV, respectively, whereas 4‐cyanothiazole complex shows well‐pronounced Urbach tail extending down to 2 eV. This spectral feature is responsible for a distinct yellow colour of the complex despite relatively high bandgap energy. The absorption maxima have Gaussian envelopes—one dominating Gaussian band and a low‐energy sub‐band), consistent with the partially localized charge transfer character of the transition (Figure S21).^[^ ^41^ ^]^ Bandgap energy for 4‐cyanothiazole derivative **3** is the highest among the studied complexes. Whereas complex 5‐cyanothiazole cannot be directly compared due to different coordination mode, the comparison with 2‐cyanothiazole shows the difference of over 0.5 eV. This huge effect can be attributed to differences in ground‐state charge distribution in the free ligand. It seems that nitrile group in 4‐cyanothiazole is 0.13 *e* more negative than in 2‐cyanothiazole (*cf*. Table 1). This may be a reason for much less efficient back donation, which results in higher energy of antibonding orbitals in the complex.

**Table 1:** Frontier orbitals, Mulliken point charges, and the distribution of electrostatic potential for various isomers of cyanothiazole ligand as calculated using the DFT technique at the BVP86/DGDZVP level of theory.

	2CNtz	4CNtz	5CNtz
HOMO	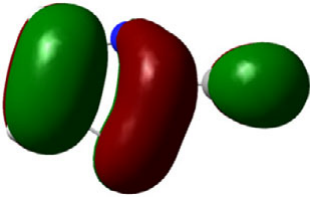	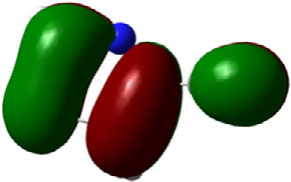	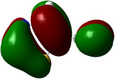
LUMO	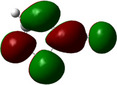	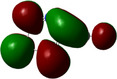	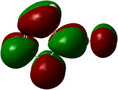
ESP	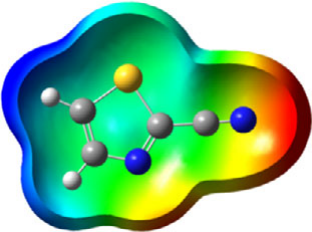	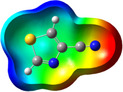	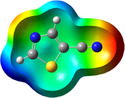
Dipole moment/D	5.17	5.26	3.39
C≡N distance/ Å	1.153	1.153	1.155
Atom	Mulliken charges in a free ligand		
C	0.136	0.012	0.014
N	−0.067	−0.075	−0.073
	Mulliken charges in a complex		
C	−0.030	0.012	0.010
N	0.112	−0.085	−0.129

**Figure 7:**
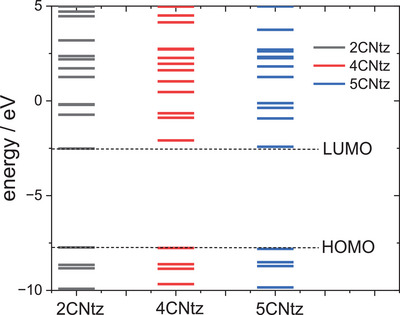
Electronic structure diagrams of cyanothiazoles as calculated using the DFT technique at the B3LYP/TZVP level of theory.

All three studies cyanothizole isomers have very similar electronic structure (Table 1, Figure 7) with both HOMO and LUMO frontier orbitals fully delocalized over whole molecules. Both HOMO and LUMO have significant antibonding character with respect to C≡N bond in nitrile substituent. This has further consequences for interaction with Cu(I) centres. All HOMOs have almost the same energy, the LUMO of 4‐cyanothiazole has slightly higher energy than other derivatives, but it seems not to have significant role. The largest negative charge can be observed in the vicinity of the nitrile group, in the case of 5‐cyanothiazole also the lone electron pair at ring nitrogen significantly contributes to negative charge areas (Table 1). These results suggest that the nitrile group should be the primary binding site for metal ions, for the 5‐cyano isomer, the ring nitrogen should also be taken into account. Therefore, two isomers, namely 2‐cyanothiazole and 4‐cyanothiazole, form very similar complexes with copper iodide, based on Cu_2_I_2_ structural motifs with only small differences in their geometry. Surprisingly, the 5‐cyano derivative forms a complex based on infinite CuI chains. This may be a consequence of the slightly different geometry of the ligand (the nitrile group and ring nitrogen atom are relatively more distant), as well as the dipole moment of 5‐cyanothiazole is also two times smaller than other ligands.

**Figure 8:**
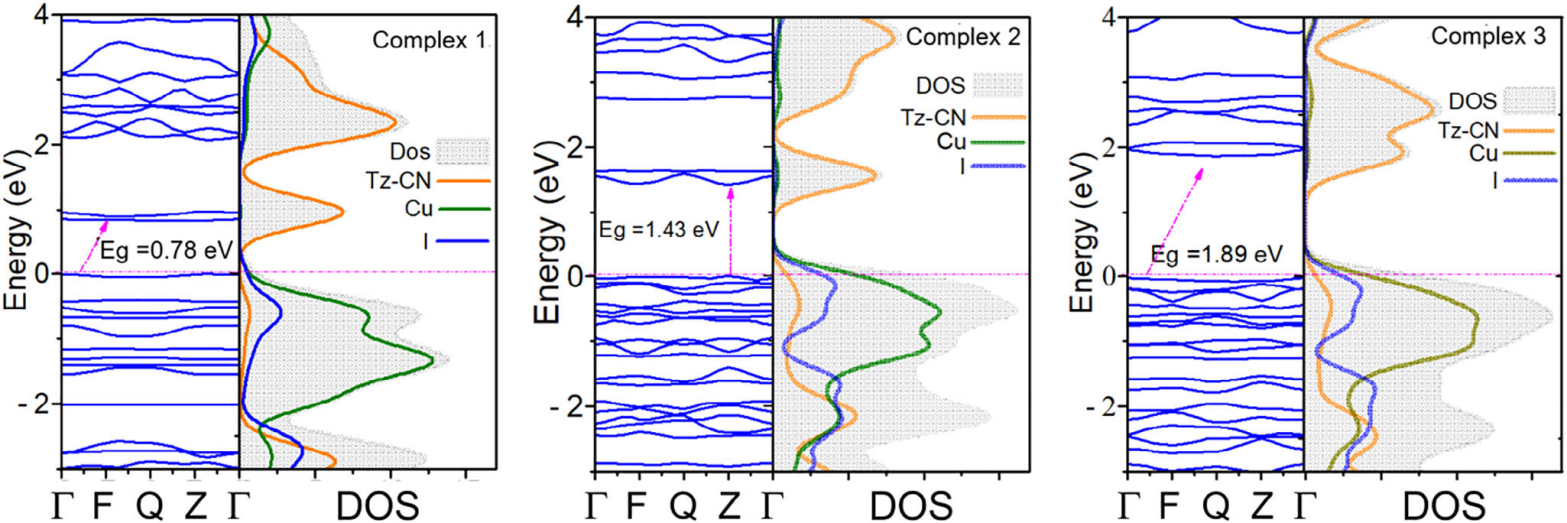
Band structure and density of states for complex **1** (**5CNtz**‐CuI), complex **2** (**2CNtz**‐CuI), and complex **3** (**4CNtz**‐CuI) at DFT‐MBD level of theory.

Despite very similar geometry and electronic structure of the three cyanothiazole ligands, the electronic structure of the corresponding Cu(I) complexes with iodide ligands is significantly different. The fully relaxed, geometry‐optimized structures of the CNtz‐CuI crystals were used to calculate their electronic structures. As shown in Figure 8, the Valence Band Maximum (VBM) and the Conduction Band Minimum (CBM) are separated near the Fermi level, confirming that all the crystals exhibit semiconducting behaviour. A closer examination of the band structure reveals that the VBM is close to the Fermi level and located at the Γ‐point, while the CBM is at the Q‐point, indicating a *p*‐type indirect bandgap (which is fully justified by the presence of copper(I) centres, which can be easily oxidized, thus yielding hoped as majority charge carriers). For the metal atoms with significant magnetic interactions, it is vital to apply spin‐orbit coupling (SOC) to accurately capture their electronic properties as it modifies the electronic structure by splitting degenerate energy levels. In our examination, we observed the bandgap is effectively reduced compared to non‐SOC calculations. This is due to the crystal field effects splitting the d‐orbitals into different energy levels, its further split with SOC. Also, SOC is creating hybridized orbitals by mixing the spin‐up and spin‐down states, which reduces the energy difference between the VBM and CBM, significantly narrowing the bandgap (Figure S22). Calculating accurate bandgaps in materials using the GGA‐PBE‐MBD method is challenging due to inherent errors. Hybrid functionals like HSE06 and PBE0, while more accurate, are computationally expensive. To address this, the DFT+U method has emerged as a promising alternative. It improves upon standard DFT functionals by introducing a correction term (the “Hubbard U”) that accounts for the energy cost of having electrons in specific localized orbitals. This approach often achieves bandgap predictions comparable to hybrid functionals, close to the experimental value, while being significantly less computationally demanding. In our study to obtain a bandgap value closely matching with the experimental data, we employed three different U values (3.5, 5.5, and 7.5 eV). The bandgap predicted by the U7.5 functional closely approximates the experimental value. All calculated band structures are shown in the supporting information (Figures S23‐S25). The bandgap values are listed in Table 2. Further to analyze the electronic nature of the studied crystals, we executed the atom‐projected DOS calculations (Figure S26).

**Table 2:** Theoretical and experimental bandgap data for cyanothiazole‐copper iodide complexes **1** – **3**.

	Theoretical bandgap (GGA‐PBE‐D)/eV						
Compound	MB	SOC	U3.5	U5.5	U7.5	Type	Experimental bandgap/eV
**1**	0.78	0.56	0.83	1.34	1.39	Indirect	2.71
**2**	1.42	1.30	1.60	1.83	1.95	Direct	2.49
**3**	1.89	1.77	2.25	2.41	2.53	Indirect	3.07

The valence band is composed mainly of 3d‐copper orbitals hybridized with 5p orbitals of iodine, with a significant contribution of organic ligand at the bottom of the band, whereas lower part of the conduction band also has a significant contribution of occupied π orbitals of ligands. 2‐ and 4‐cyano derivatives also show a small contribution of ligand π‐orbitals to the top of the valence band. The bottom of the conduction band is built mainly from π‐orbitals of the ligand, with a minor admixture of copper‐ and iodine‐based orbitals. This contribution becomes slightly more significant in the upper part of the conduction band for the 5‐cyano derivative. Such arrangement of energy levels indicates that organic ligands play the role of electron acceptors, thus decreasing the bandgap of these materials, as compared to the parent CuI material (E_g_ = 3.1 eV).^[^ ^42^ ^]^

**Figure 9:**
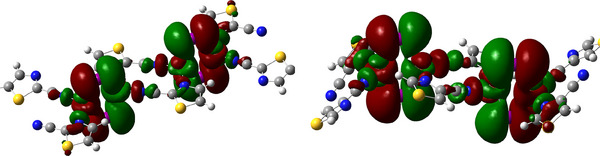
Contours of HOMO orbitals calculated for a relaxed‐geometry fragment of polymeric chains of complex **2** (**2CNtz**‐CuI, left) and **3** (**4CNtz**‐CuI, right) complexes as calculated using DFT approach at the BVP86/DGDZVP level of theory.

From the molecular orbital point of view (Figure 9), interactions between copper(I) ions and cyanothiazole ligands (2‐, and 4‐cyano isomers) are threefold: (i) coordination via lone electron pair at thiazole ring, (ii) coordination via lone pair at nitrile nitrogen and (iii) back donation from occupied $3d_{z^{2}}$ of copper, hybridized with 5*p_x_* orbitals of iodine, to the antibonding π‐orbital of the nitrile group. The latter bonding mode can be observed directly as a significant elongation of the C≡N bond on ligation with the copper centre (∼1.15 Å in the ligand, ∼1.18 Å in complexes). Surprisingly, the same effect is observed for the 5‐cyano derivative, in which there is no direct interaction between Cu(I) centres and nitrile groups. In this particular case, this effect may be attributed to electron flow from the Cu‐I ribbon to the nitrile group via the ring. Analysis of Mulliken point charges confirms this interpretation: on ligation, the total charge of the C≡N moiety increases from +0.041 to −0.119 e (Table 1).

It should be anoted that the energy dispersion of the top of the valence and the bottom of the conduction band is rather small, which should result in relatively high effective masses of charge carriers, i.e., low electron and hole mobilities. It is fully justified, as any of the structures under study does not provide significant π···π stacking interactions between neighbouring aromatic ligands, and the Cu‐I polymeric ribbons in 5‐cyano complex **1** extend only in one dimension. Therefore, as low mobility materials, copper iodide complexes with cyanothiazole ligands can find potential application as luminescent materials, catalysts, or memristive materials; however, they are not well‐tailored for applications that require high mobility, e.g., field effect transistors.

### 2.4 X‐ray absorption spectroscopy

**Figure 10:**
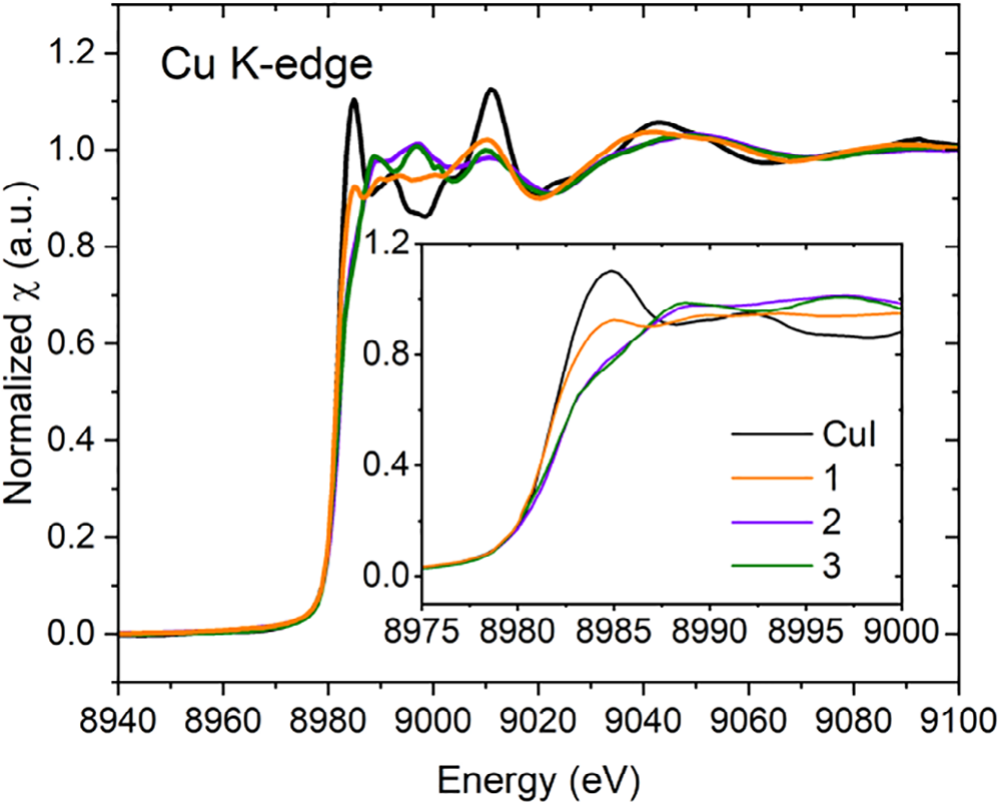
Experimental XAS Cu K‐edge spectra for the CuI reference and its complexes with cyanothiazole isomers.

**Figure 11:**
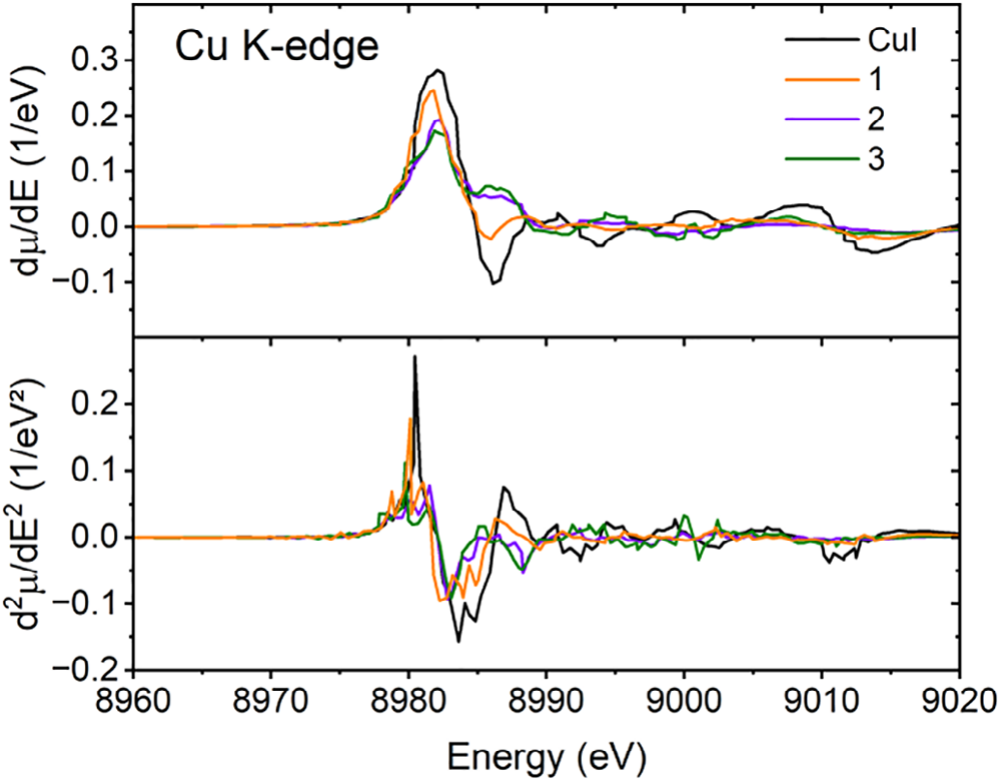
First (top) and second (bottom) derivatives with respect to energy of the experimental Cu K‐edge XAS spectra for the CuI reference and the studied complexes with cyanothiazoles.

X‐ray absorption spectroscopy (XAS) is an experimental technique that directly probes the electronic and structural nature of elements. In this work, XAS helped us to understand the nature of copper sites in the studied CuI complexes with cyanothiazoles compared to the reference CuI. We measured Cu K‐edge spectra (Figure 10), where the sharp increase in the absorption–the edge–defines the threshold ionization energy. The value of the absorption edge usually increases with the oxidation state of the absorber element due to an increase in the core binding energy. The formal oxidation state of copper in all the studied materials is +1. Hence, the edge values are very close to each other and are 8982.05 eV for CuI reference, 8981.71 eV for **1**, 8982.14 eV for **2**, and 8981.95 eV for **3**. They were determined as the maximum of the first derivative of the spectrum with respect to energy (Figure 11).

The X‐ray absorption near‐edge structure (XANES) region typically spans from about 20 eV below to approximately 50–100 eV above the absorption edge. The extended X‐ray absorption fine structure (EXAFS) region usually begins at around 50–150 eV above the edge and can extend to much higher energies, often up to 1000 eV above the edge. XANES oscillations or their absence reflect the electronic and geometric characteristics of the molecular environment of the absorber. The basic electron configuration of copper is [Ar]3d^10^4s^1^, while the studied Cu(I) complexes with formal +1 oxidation state should reveal [Ar]3d^10^. The main features in XANES result from the electronic transitions from 1s orbital, primarily to dipole‐allowed 4p orbitals. Pre‐edge features arise from quadrupole‐allowed 1s → 3d transitions, which gain intensity via d‐p orbital hybridization. Depending on the element, the pre‐edge may also contain features related to transitions to higher bound states within the discrete spectrum.

Figure 10 shows Cu K‐edge XANES for the studied complexes and CuI reference. Only for the reference CuI, a distinct white line is observed, while spectra for the cyanothiazole complexes exhibit rather a step‐like course. This is related to the higher density of unoccupied states available for electronic transitions in CuI and, therefore, more localized absorption. The same spectral feature, but of much lower intensity, can be observed for the 5‐CNtz derivative. It is fully justified, as the CuI ribbons offer a much higher density of hybridized Cu and I states than other complexes. On the other hand, the iodide ions may be highly polarizable, leading to significant electron redistribution. Such localized interaction may enhance the metal‐ligand hybridization and create a more pronounced density of unoccupied states, resulting in a high white line intensity. Salomon et al. studied XANES of several dozen Cu(I) and Cu(II) model complexes.^[^ ^43^ ^]^ They also investigated the edge features of Cu(I) with ligand field theory. Since the 3d orbital in Cu(I) is fully occupied for Cu(I), the pre‐edge peak was assigned to the dipole‐allowed transitions, where the threefold degeneracy of 4p_x,y,z_ orbitals in free Cu(I) is split in the ligand field. Then, for linear 2‐coordinate D_∞h_ complexes, this would result in intense 1s → 4p_x,z_ pre‐edge at lower energies and 1s → 4p_z_. For T‐shaped 3‐coordinate compounds (C_2v_), this ends up in further degeneracy of 4p_x,y_, to obtain separate lower energy 1s → 4p_x_ pre‐edge and low‐intensity, higher energy 1s → 4p_z_ feature. In the 3‐coordinate trigonal planar structure (C_3_ _h_), the 4p_y_ and 4p_z_ will be degenerated and shifted to a higher energy than 4p_x_, which is responsible for the pre‐edge. Finally, in 4‐coordinate tetrahedral (T_d_) complexes, the 4p orbitals should be close to degenerate, but shifted to higher energy compared to free Cu(I), where the intensity of each 1s → 4p_i_ is reduced. It is worth noting that the Cu(I) complexes discussed here were characterised by N, O, and S ligation (or their mixtures), while in this work, we study, the electron donors are nitrogen and iodide. Yet, the data on XAS for CuI or its complexes is scarce.^[^ ^44^ ^]^

Similarly to the tetragonal 4‐coordinated complexes reported by Salomon et al.,^[^ ^43^ ^]^ we do not observe distinguishable separate pre‐edge peak at 8984 eV, as for Cu_2_O (I).^[^ ^45^ ^]^

In Figures S27–S29, we show the DFT‐calculated XAS spectra and density of states compared with experimental data for the studied CuI complexes. The calculated spectra are consistent with the experimental data. The main edge results from the dipole‐allowed 1s → 4p transition. After deconvolution into separate molecular orbitals, the calculated PDOS splits into the lower‐energy (< 8980 eV) and the higher‐energy (> 8980 eV) edge features from different p‐type orbitals. On the other hand, d‐type orbitals contribute to higher‐energy features (>8985 eV) and also would be responsible for the pre‐edge, which is not present in the experimental spectra.

### 2.5 Photoluminescence Studies of CNtz‐CuI Complexes

All three complexes excited with UV light (375 nm, 3.59 eV), both at room and low temperature, emit yellow‐greenish to orange, naked‐eye visible photoluminescence, which is a typical behaviour of copper iodide derivatives (Table 3, Figure S30).

**Table 3:** CIE 1931 colour coordinates and photoluminescence quantum yields determined for compounds copper iodide cyanothiazole derivatives.

Compound	Photoluminescence quantum yield/%	CIE1931 coordinates
**1**	11.9	(x,y) = (0.346, 0.597)
**2**	9.89	(x,y) = (0.518, 0.471)
**3**	8.64	(x,y) = (0.389, 0.569)

**Figure 12:**
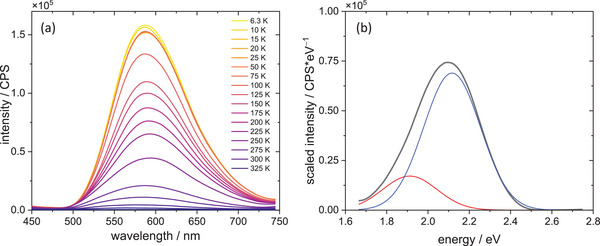
Emission spectra of complex **2** (a) along with deconvolution of the spectrum recorded at 6 K into Gaussian components (b).

**Figure 13:**
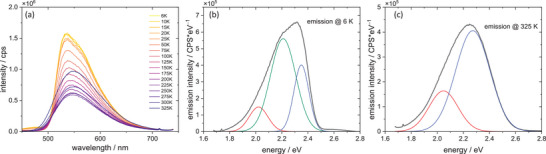
Emission spectra of complex **3** (a) along with deconvolution into Gaussian components of the spectra recorded at 6 K (b) and 325 K (c).

**Figure 14:**
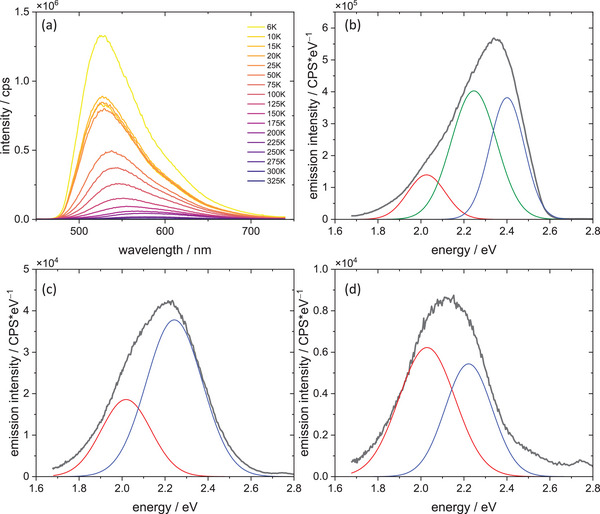
Emission spectra of complex **1** (a) along with deconvolution into Gaussian components of the spectra recorded at 6 K (b), 150 K (c), and 225 K (d).

**Figure 15:**
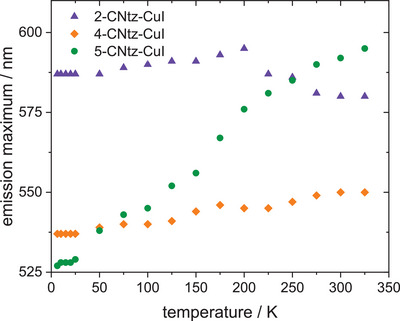
The changes in PL max peak for all complexes at different temperatures.

Fluorescence spectra were measured for all compounds in a wide temperature range (6–325K). All studied compounds show dual emission—at low temperatures, even three different emission components can be isolated – as shown in Figures 12, 13, 14. The emission maxima gradually shift to lower energies for **1** and **3,** the latter one shows the bathochromic shift of ca. 13 nm during 6 K–325 K temperature scan, whereas **1** shows the bathochromic shift of 58 nm. (Figure 15) At the same time, emission intensity decreases with temperature, and the full width at half maximum increases with increasing temperature. Compounds **1** and **3** have very similar CIE1931 coordinates, yellow‐green hues. The emission colour of compound **2** is notably different from the first two. The higher x value and lower y value shift it away from the green region and toward the warmer part of the diagram, possibly in the orange or reddish range, which is consistent with the visual perception of luminescence of these species.

**Figure 16:**
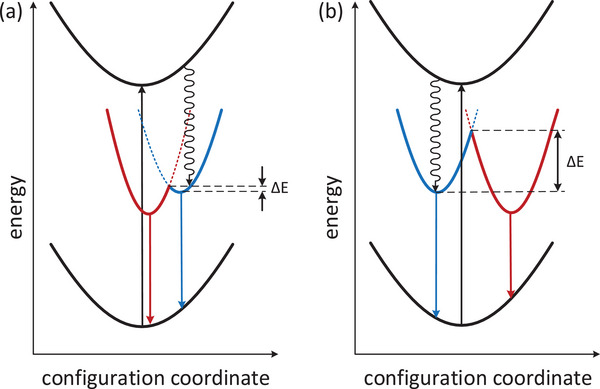
Schematic diagram of photophysical processes with two self‐trapped emitting states (red and blue lines) with a) small and b) large potential barriers. Please note that energies of low‐energy emissive excited states are identical in (a) and (b); the only difference is the configurational coordinate (molecular displacement in the Frank‐Condon state).

The spectral data indicate the presence of at least two different emissive excited states. Analysis of electronic structure (density of states in particular) suggests three possibilities of low‐lying excited states: MLCT (copper → cyanothiazole), MLCT (copper→iodide) (which can also be regarded as a local excited state (LE) involving hybridized orbitals of iodide and copper centres), and LLCT (iodide→cyanothiazole). The same set of transitions has been suggested for other copper iodide adducts with organic ligands.^[^ ^46^ ^]^ The spectral data suggests that excitation to a higher excited state is followed by thermalization to low‐energy excited states of different charge transfer character and different internal reorganization (lower for LE state and much higher for MLCT state. The interchange between these states is possible via the thermally activated intersystem crossing. On this basis a tentative energy diagram can be constructed (Figure 16). In the case of a low interconversion energy barrier (Figure 16a) dual emission is not observed (or just a minor change of emission maximum can be observed). This is the consequence of an interplay between the ground and excited state potential energy curve, and also depends on the reorganization energy. If the molecular displacement is small, then we should observe almost the same emission energy from both excited states, as shown in Figure 16a. This is the situation observed in the case of **2CNtz**‐CuI, complex **2**. Despite that detailed shape analysis shows two distinct emission bands (Figure 12b) at 1.91 and 2.11 eV, which may correspond to two different excited states, which, due to a low energy barrier, are in thermal equilibrium at any temperature. Similar situation, however, with slightly larger special separation of relaxed excited states is observed in the case of **3**. Slightly different spatial arrangement of the organic ligand may result in a slightly higher activation barrier, therefore weak bathochromic shift is observed with increasing temperature.

More spectacular spectral changes can be observed for the **5CNtz**‐CuI complex **1**. A large bathochromic shift of the emission peak corresponds to the systems with high activation energy between two emissive states. Furthermore, at low temperatures, a third peak is clearly visible. Broadening of bands as well as a decrease in intensity with increasing temperature is also clearly visible.

Broadening of bands may be used to evaluate the Huang‐Rhys parameter, which is the measure of photon‐exciton coupling, as given by Equation 4:

(4) $$FWHM=2.36\sqrt{s}\hbar \omega_{photon}\sqrt{\coth\frac{\hbar \omega_{photon}}{2k_{B}T}}$$

where FWHM is the full width at half maximum (derived from deconvolution of emission spectra into Gaussian components), *s* is the Huang–Rhys factor, and *ω_phonon_* is the phonon energy. Fitting of Equation 4 to experimental data yields Huang–Rhys factors of 15.21, 12.52, and 39.8 for **2CNtz**‐CuI, **4CNtz**‐CuI, and **5CNtz**‐CuI, respectively. Photon energies amount 34.7, 19.8, and 13.0 meV, respectively. The latter, much higher value is justified by the specific structure of the complex–ribbon‐like CuI core decorated by the 5‐cyanothiazole ligands. Stokes shifts for emission has been evaluated on the basis of optical bandgap (as the solid state absorption spectra do not show any well‐pronounced maximum) and the maximum of emission (irrespectively on the contributing Gaussian components) recorded at ca. 6 K. Values calculated for **1**, **2**, and **3** are 0.77, 0.44, and 0.82 eV, respectively. Stokes shifts for **1** and **3** are comparable, which is in line with a complex photophysical behaviour related to the interconversion of different excited states. The significantly smaller value calculated for complex **2** is a result of a small energy barrier between different excited states, which corresponds to less efficient energy dissipation upon excitation.

**Figure 17:**
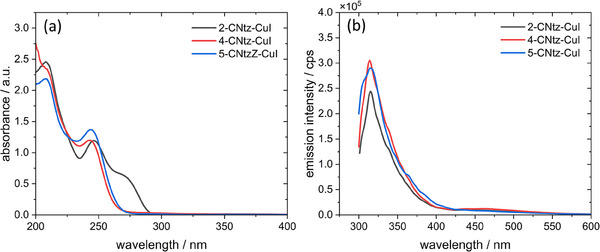
Absorption (a) and emission (b) of copper iodide complexes with different cyanothiazole isomers in acetonitrile. The excitation wavelength was set to 275 nm.

Interestingly, acetonitrile solutions of the studied complexes are significantly different – they show features of the organic ligand with some increased absorption in the deep UV region (Figure 17a). A well‐pronounced absorption band at ca. 250 nm is observed for all three complexes, whereas 2‐cyanothiazole derivative (**1**) shows an additional shoulder at ca. 275 nm. These spectra closely resemble those of pure organic ligands (Figure S31a), the only difference is strong absorption in the deep UV range (200‐230 nm). Emission spectra of solutions (Figure 17b) are almost identical for all studied compounds with a single emission peak at ca. 315 nm (observed with 275 nm excitation). The same emission is observed for pure ligands (Figure S31b). This leads to conclusion that all three complexes dissociate in acetonitrile solutions due to the relatively weak interaction of copper(I) centres with nitrile ligands, moreover thiazole system is only a weak electron donor.^[^ ^47^ ^]^ This justifies the stability of these complexes in solid phase, however acetonitrile itself is a nucleophile strong enough to coordinate to copper(I) centres and replace cyanothiazole ligand. The same conclusion can be drawn on the basis of ^1^H and ^13^C NMR spectra (cf. Figures S32‐S44).

In conclusion, all cyanothiazole adducts with copper(I) iodide are luminescent materials with dual‐type emission, however, **2CNtz** and **4CNtz** isomers yield polymers with periodically arranged Cu_2_I_2_ fragments separated by organic ligands. Therefore, internal reorganization energy is low, which is reflected in a low activation barrier between two emissive excited states, and dual emission features are barely visible. The **5CNTz** in turn yields polymeric ribbons decorated with organic ligands. Therefore, the metal‐to‐ligand charge transfer transition is accompanied by much larger geometry displacement as compared to other polymers. As a consequence, the activation energy barrier between two emissive states is much higher due to increased internal reorganization energy and temperature‐dependent dual emission is a dominating spectral feature of the **5CNtz**‐CuI complex **1**.

### 2.6 Electrical Measurements

**Figure 18:**
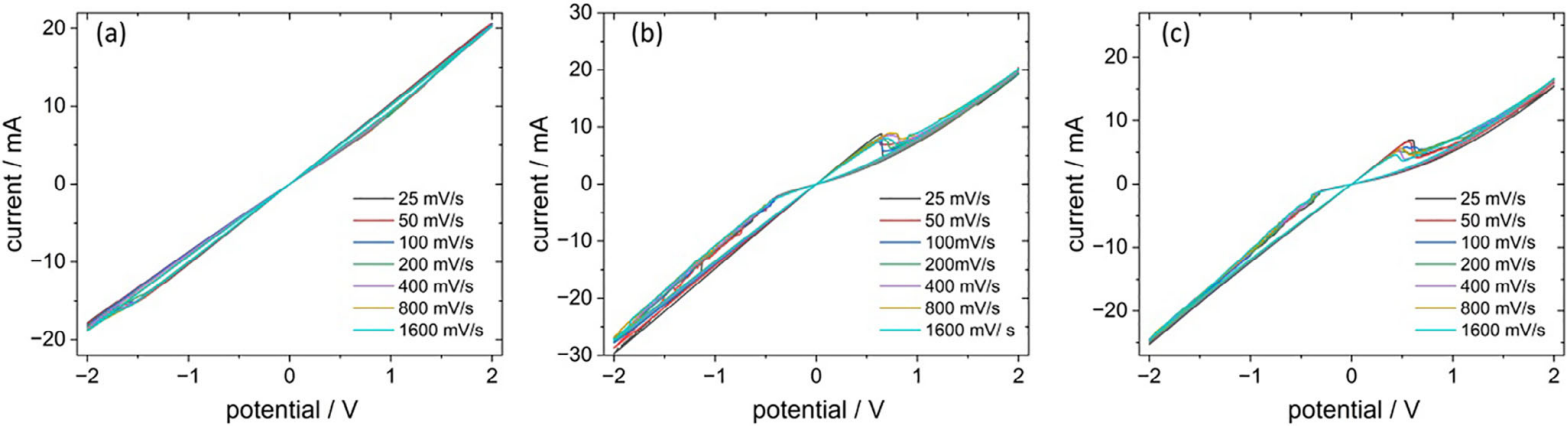
Hysteresis curves recorded for complexes **1** (a), **2** (b), and **3** (c) for various scan rates.

For electrical measurements for information processing, layered devices were prepared, and their current—voltage (I‐V) characteristics were measured at room temperature. Each of the compounds presented a hysteresis curve; however, with respect to behavior in the 1^st^ quadrant, complex **2** and **3** were characterized by the clockwise switching, and complex **1** by the counter‐clockwise switching (see Figures 18a‐c).

**Figure 19:**
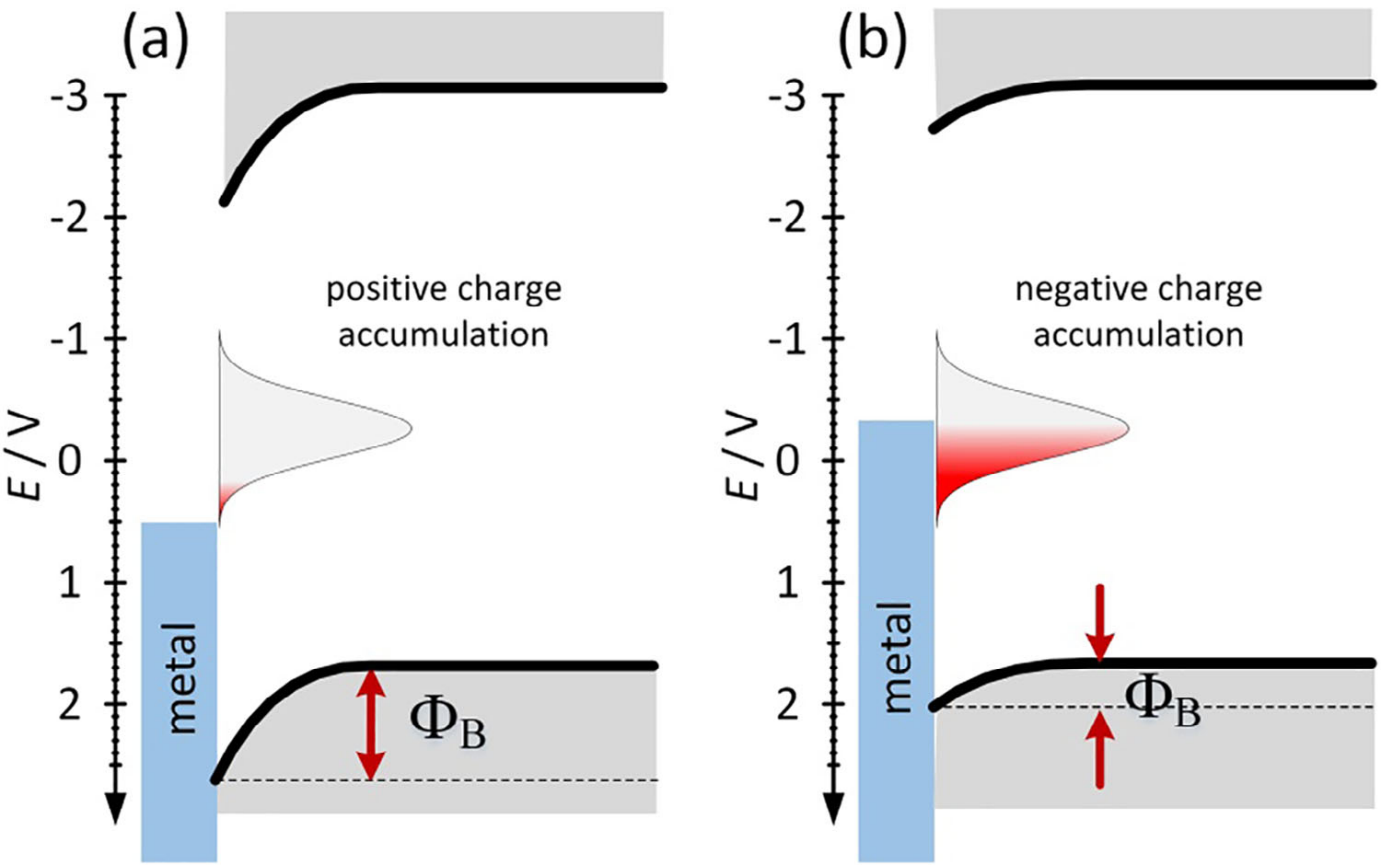
A tentative mechanism of resistive switching (Schottky barrier modulation) in studied devices based on **2** and **3**: electronic structure diagram for the high‐resistive state (a) and the low‐resistive state (b).

Scan rate‐independent hysteresis observed for all compounds indicates the lack of conductive filaments and suggests an interfacial mechanism based of electron trapping at metal‐induced gap states (MIGS).^[^ ^48^ ^]^ Other words, the resistive switching involves modulation of a Schottky barrier formed between semiconducting metal complexes and metallic electrodes due to electrostatic repulsion between band electrons and electrons trapped at MIGS. The clockwise direction of the hysteresis loop observed for **2** and **3** indicates *p*‐type conductivity of these materials, whereas the anticlockwise loop observed for **1** infers *n*‐type conductivity of this material.^[^ ^19a, b^ ^]^ This may be justified by the very different bonding patterns of **2** and **3** as compared to **1**. All complexes, irrespective of their conductivity type, with copper top electrodes form leaky Schottky junctions with rectification factors (ratio forward to reverse current) of 1.10, 1.41, and 1.56 for **1**, **2**, and **3**, respectively (Figure 18). The resistive switching in **1** is rather smooth and barely visible with a very low ON/OFF ratio. The ON/OFF ratios for **2** and **3** are significantly larger (ca. 2 at 0.5 V) and are accompanied by abrupt conductivity changes at 0.65 V (low‐to‐high resistive state transition) and ‐0.39 V (high‐to‐low resistive state transition) for **2**. For complex **3**, these processes occur at 0.54 and −0.32 V. The negative differential resistance observed at ca. 0.5–0.7 V is related to the gradual detrapping of electrons from MIGS, which reduces current intensity with increasing bias voltage. These potential values correspond to pseudo‐redox processes associated with depopulation (positive bias) and population (negative bias) of MIGS, which directly controls the Schottky barrier height, hence is responsible for modulation of electrical conductivity (Figure 19).

**Figure 20:**
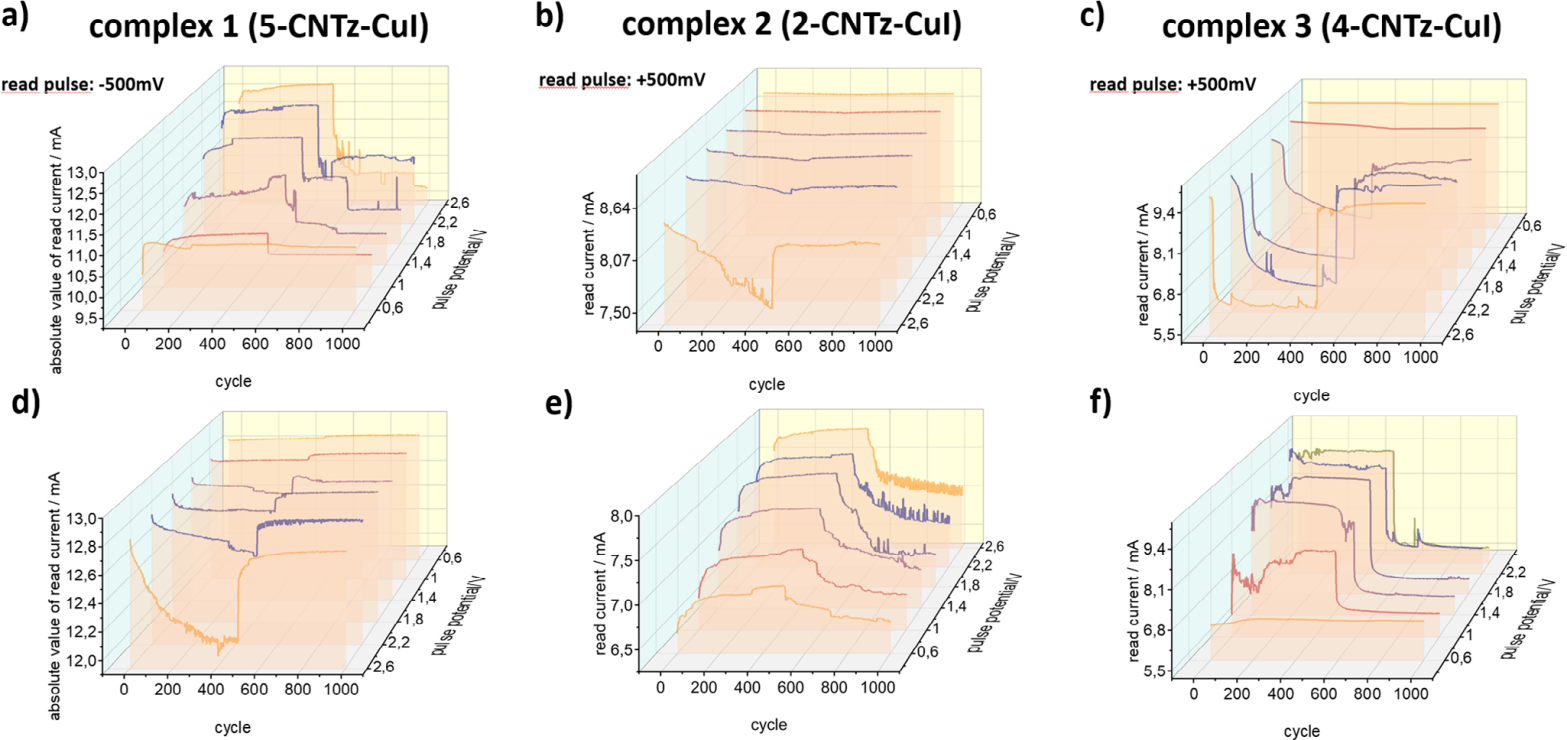
Basic electrical and plasticity behavior characterization. I–V characteristic for the a) complex **1** (**5CNtz**‐CuI), b) complex **2** (**2CNtz**‐CuI), and c) complex **3** (**4CNtz**‐CuI). Potentiation‐depression experiments: row d)–f) after conditioning samples in −3 V, followed by a positive and negative set of switching pulses. Row g)–i) after conditioning samples in +3 V, followed by a negative and positive set of switching pulses. In case of negative readout (complex **1**) values, the current is presented as an absolute value, allowing easy comparison of the different behaviour types for similar conditions.

These characteristics directly influence the pulse behavior. For the first two compounds positive pulses decrease the conductivity, whereas negative pulses increase conductivity. Sample complex **1** (Figure 20a,d) can be potentiated with pulses over +1 V (signal intensity varies throughout the sequence), and depression can be achieved for pulses of ‐1.4 V or higher. In all cases, gradual switching is reversible. The incremental switching behavior patterns—either in a form of potentiation or depression are shown for complex **2** in Figure 20b,e. Depression behavior is registered above threshold of +2.2 V for switching pulses, whereas potentiation phenomena are available with only low values of switching pulse potentials—as low as −0.6 V. Figure 20c,f show results for complex **3**, for which sample the depression is possible with switching pulse potentials of +1.4 V, potentiation starts for −1 V pulses.

These characteristics directly influence the pulse behavior. For the first two compounds positive pulses decrease the conductivity, whereas negative pulses increase the conductivity. Sample **5CNtz**‐CuI complex **1** (Figure 20a,d) can be potentiated with pulses over +1 V (signal intensity varies throughout the sequence), and depression can be achieved for pulses of ‐1.4 V or higher. The incremental switching behavior patterns—either in a form of potentiation or depression are shown for 2CNtz complex **2** in Figure 20b,e. Depression behavior is registered above the threshold of +2.2 V for switching pulses, whereas potentiation phenomena are available with only low values of switching pulse potentials—as low as ‐0 .6V. Figure 18c,f show results for 4CNtz complex **3**, for which sample the depression is possible with switching pulse potentials of +1.4 V, and potentiation starts for ‐1 V pulses. In all cases, gradual switching is reversible.

**Figure 21:**
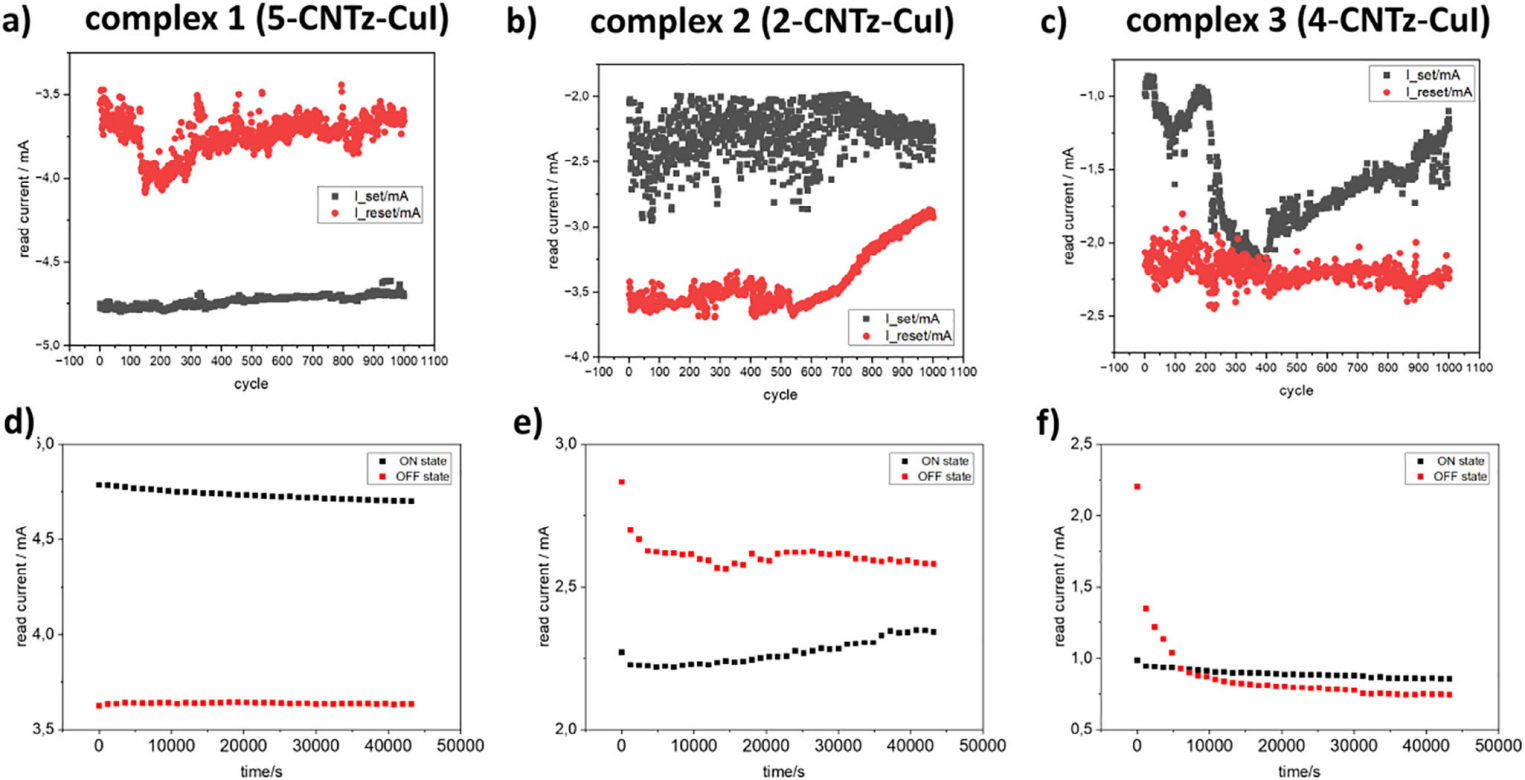
Device endurance and state retention tests. Results of the on‐off switching (endurance) tests for: a) complex **1** (**5CNtz**‐CuI), b) complex **2** (**2CNtz**‐CuI), and c) complex **3** (**4CNtz**‐CuI). Results of the retention tests for the HRS and LRS states for the same complexes: d) **1**, e) **2**, and f) **3**.

Regarding electrical endurance (on‐off test) and stability of the switching (retention tests) for the devices, complexes **1** (Figure 21a,d) and **2** (Figure 21b,e) can be switched in repeatable and predictable manner, with both LRS and HRS states stable for the period of 12h. Complex **3** (Figure 21c,f), however, switches randomly over 1000 cycles and is characterized by instability of one of the states (over 1.5 h).

## 3 Conclusion

In this work, we synthesized previously unreported CuI complexes with cyanothiazole ligands and showed both experimentally and theoretically that the CN group's position on the thiazole affects the complexes’ structure, and thus their optical and electrical properties. The copper(I) iodide adducts form coordination polymers: the 5CNtz derivative (complex **1**) forms polymeric ribbons with decorated organic ligands, while the 2CNtz and 4CNtz derivatives **2** and **3** feature Cu₂I₂ units periodically separated by organic ligands. All compounds are luminescent with dual emission. Complexes **2** and **3** have low internal reorganization energy, yielding a small activation barrier between emissive states and barely perceptible dual emission. In contrast, the MLCT transition in complex **1** involves a larger geometric displacement and higher activation energy, resulting in a temperature‐dependent dual emission. Cu K‐edge XAS confirmed the +1 oxidation state of copper in all complexes and revealed that complexes **2** and **3** exhibit slightly lower Cu–I *p*‐orbital hybridization compared to complex **1**, both being less hybridized than a standard CuI. DFT further confirmed their *p*‐type semiconductor characteristics, suggesting potential for hole‐based electronic applications. All three complexes form leaky Schottky junctions with copper electrodes (complex **1** being *n*‐type, while complexes **2** and **3** are *p*‐type), with resistive switching driven by modulation of the Schottky barrier via electron trapping/detrapping at metal‐induced gap states. In information processing, only complexes **1** and **2** reliably switch between high and low resistance states, making complex **1** a candidate for material‐based neural networks due to its gradual switching profile, and complex **2** more suitable for memory applications, despite requiring electrode optimization to improve its low ON/OFF ratio. Meanwhile, complex **3** appears more promising for volatile behavior or random number generation applications.

## 4 Experimental

### 4.1 Syntheses

#### 4.1.1 General Considerations

The following chemicals were used as purchased: copper (I) iodide CuI, 98.0%, WARCHEM; 5‐cyanothiazole (**5CNtz**), 97%, Angene; 2‐cyanothiazole (**2CNtz**), 99.24%, AmBeed; 4‐cyanothiazole (**4CNtz**), 97%, Angene;acetonitrile ACN, 99.9%.

#### 4.1.2 Synthetic Protocols

**Compound 1 [(5CNtz)CuI]_∞_**: A solution of CuI (0.086 g, 0.0452 mmol), was prepared in 3 mL of ACN. Solid **5CNtz** (0.05 g, 0.0452 mmol) was added to the solution, and the reaction mixture was stirred until complete dissolution of the substrate. The resulting reaction mixture was subjected to crystallization by slow evaporation of the solvent under controlled conditions. After approximately 24 hours, well‐defined yellowish crystals were obtained, yield: 86%, decomposition temperature above: 217.3 °C. Elemental analysis: calcd 15.98% C, 0.67% H, 9.32% N; found 15.97%C, 0.69%H, and 9.33%N. FTIR ATR: 3092(m), 3072(versus), 2227(versus), 1505(m), 1375(m), 1308(s), 1231(vs0, 1114(s), 896(s), 820(s), 597(m) cm^−1^. NMR, for the symbols of atoms and the spectra see Figures S32 –S44 of SI: ^1^H NMR (400 MHz, CD_3_CN‐*d*_3_): δ = 8.52 (s, 1 H, **H_b_**), 9.21 (s, 1 H, **H_a_**); ^13^C{^1^H} NMR (100 MHz, CD_3_CN‐*d*_3_): δ = 106.3 (s, **C_d_**), 111.9 (s, **C_b_**), 153.0 (s, **C_c_**), 159.8 (s, **C_a_**).

**Compound 2 [(2CNtz)CuI]_∞_**: **2** was synthesized using the same procedure as described for compound **1**, well‐defined yellow crystals were obtained, yield: 68%, decomposition temperature above: 137.1 °C. Elemental analysis: calcd 15.98% C, 0.67% H, 9.32% N; found 15.96%C, 0.66%H, and 9.34%N. FTIR ATR: 3098(w), 3082(versus), 2231(w), 1464(m), 1351(s), 1309(s), 1202(w), 1136(versus), 1051(s), 909(w), 885(m), 733(w), 770(versus), 702(w), 619(m), 524(m), 492(w) cm^−1^. NMR, for the symbols of atoms and the spectra see Figures S32 –S44 of SI: ^1^H NMR (400 MHz, CD_3_CN‐*d*_3_): δ = 8.00 (d, ^3^J_HH_ = 3.057, 1 H, **H_c_**), 8.12 (d, ^3^J_HH_ = 3.057, 1 H, **H_b_**); ^13^C{^1^H} NMR (100 MHz, CD_3_CN‐*d*_3_): δ = 113.0 (s, **C_d_**), 126.9 (s, **C_c_**), 136.4 (s, **C_b_**), 145.2 (s, **C_a_**).

The **unstable form of compound 2** was synthesized following the same procedure as described for compound **1**, with the modification that the solvent was quickly evaporated to yield a reddish powder. FTIR‐ATR (cm⁻¹): 3103(s), 3112(s), 3084(versus), 2233(s), 1478(m), 1468(m), 1373(s), 1307(w), 1189(w), 1134(s), 1057(m), 883(w), 766(s), 753(s), 617(w), 532(w), 487(w) cm^−1^.

**Compound 3 [(4CNtz)CuI]_∞_**: **3** was synthesized using two methods described below, decomposition temperature above: 155.5 °C. Elemental analysis: 15.98% C, 0.67% H, 9.32% N; found 15.99%C, 0.69%H, and 9.30%N. FTIR‐ATR: 3094(versus), 3073(versus), 1416(s), 1299(m), 1213(m), 1130(m), 986(versus), 8819 m), 843(versus), 784(versus), 504(s) cm⁻¹. NMR, for the symbols of atoms and the spectra see Figures S32 –S44 of SI: ^1^H NMR (400 MHz, CD_3_CN‐*d*_3_): δ = 8.41 (d, ^4^J_HH_ = 1.834, 1 H, **H_c_**), 9.05 (d, ^4^J_HH_ = 1.956, 1 H, **H_a_**); ^13^C{^1^H} NMR (100 MHz, CD_3_CN‐*d*_3_): δ = 114.2 (s, **C_d_**), 126.9 (s, **C_c_**), 132.2 (s, **C_b_**), 156.2 (s, **C_a_**).

Method A: A solution of CuI (0.086 g, 0.0452 mmol) was prepared in 3 mL of ACN. Solid **4CNtz** (0.05 g, 0.0452 mmol) was then added to the solution, followed by the addition of 1 mL of distilled water. The reaction mixture was stirred until the substrate completely dissolved and left for crystallization at 5–8 °C. A white crystalline product was subsequently obtained. Yield: 46%

Method B: The synthesis procedure was identical to that described for compound **1**, except that the solvent was evaporated to 1 mL of the initial volume of the reaction mixture. This resulted in the formation of a crystalline yellowish powder. Yield: 52%

### 4.2 Computational Methods

DFT modeling of single ligand molecules as well as fragments of polymeric chains has been performed using the Gaussian 16 Revision C.01 software package^[^ ^49^ ^]^ and post‐processed and visualized using the GaussView package.^[^ ^50^ ^]^ For organic molecules, the B3LYP hybrid functional^[^ ^51^ ^]^ and the TZVP basis set^[^ ^52^ ^]^ have been used, whereas metal complexes were modelled using the BVP86 hybrid functional^[^ ^53^ ^]^ and the DGDZVP Gaussian‐type double zeta basis set.^[^ ^54^ ^]^

The geometry optimization and the band structure and the density of states were obtained, were calculated using CASTEP (Cambridge Serial Total Energy package) code. The plane‐wave basis set and pseudopotentials were used, implemented with the PBE‐GGA exchange‐correlation functional. The calculations employed a plane‐wave basis set with pseudopotentials to carry out the PBE‐GGA (Perdew‐Burke‐Ernzerhof Generalized Gradient Approximation) exchange‐correlation functional. Ion‐electron interactions were described using the projected augmented wave (PAW) formalism. Non‐covalent interactions were included through the DFT‐MBD (Density Functional Theory with Many‐Body Dispersion) relativistic correction method. To improve the accuracy of the electronic structure, we employed the DFT‐MBD+*U* method with the GGA‐PBE exchange‐correlation functional. The U_eff_ parameters are employed for the localized 3d electrons of the Cu. To ensure exact convergence, the periodic boundary conditions were set with the following parameters: a plane‐wave cut‐off energy of 580 eV, an energy convergence tolerance of 5 × 10⁻⁷ eV/atom, a maximum force tolerance of 0.01 eV/Å, a maximum stress tolerance of 0.02 GPa, and a displacement tolerance of 5 × 10⁻⁴ Å. A Monkhorst‐Pack grid of 5 × 5 × 5 k‐points was applied for all calculations.

XAS spectra were modelled using density functional theory (DFT) calculations performed with FDMNES software^[^ ^55^ ^]^ using local spin density approximation. The structure for the calculations was previously DFT‐optimized using CASTEP. The finite difference method was used for X‐ray absorption fine structure^[^ ^56^ ^]^ with dipole (Δl = ±1) and quadrupole (Δl = 0, ±2) transitions and 9 Å cluster radius. Relativistic and spin‐orbit coupling effects were neglected. Lorentzian convoluted spectra were presented.

### 4.3 Electrical Measurements

Thin film samples were prepared according to the following procedural steps. Firstly, all compounds were dissolved in the mixture of DMSO (typically 30 mg of compound in 1 mL of DMF) and mixed on a magnetic stirrer at elevated temperatures (100 °C). Substrates, ITO glass (Ossila, The Netherlands) were washed (water, isopropyl alcohol), dried, and cleaned with O_2_ plasma, then heated to 100 °C. Thin films were deposited on hot substrates via the spincoating technique, typically 3000 rpm for 30 s, and post‐baked at 100 °C on a hotplate for 25 min. Copper electrodes were thermally deposited afterwards, through shadow mask (Ossila, The Netherlands) with electrode dimensions 1.3×1.5 mm.

All of the I‐V responses, state retention measurements, endurance tests, and potentiation‐depression tests were registered on a SP‐300 potentiostat (BioLogic, France) equipped with an Instec TP102 V Thermoelectric Probe Station. The system was designed as a two‐terminal device, with the working electrode (WE) connected to Cu electrodes, and the counter (CE) and reference (RE) electrodes were connected to the ITO substrate.

Potentiation‐depression tests were conducted two‐way. Firstly, the devices were switched to either high‐resistance state (HRS) or low‐resistance state (LRS). In the next step a set of 500 pulses of one polarity was followed by a set of 500 pulses of reversed polarity. Device state was read after each incremental switching—either by 200 mV or −200mV. The change in reading voltage was to increase S/N ratio and was based on the shape of the hysteresis loop (see Figure 17(a)–(c)).

The endurance test (on‐off switching) was conducted by subjecting the devices to alternating extreme potential values (‐2 V and +2 V) for 100 ms. This switching sequence was repeated 1000 times, with the device state measured at a reading voltage of either ‐200 mV after each cycle.

State switching stability (state retention) was evaluated for both HRS and LRS. After applying a DC bias of either ‐2 V or +2 V for 1 s, the device state was measured at an arbitrary chosen reading voltage of +200 mV every 20 min over a period of 12 h.

### 4.4 Physicochemical Methods

FTIR spectra of the pure crystalline products were recorded using a Nicolet iS50 spectrometer equipped with a Specac Quest diamond ATR accessory. All FTIR spectra were collected and processed using OMNIC software.

Elemental CHNS analyses were performed on a Vario EI Cube Elemental Analyzer.

The melting points of the compounds were determined using a Stuart Scientific SMP3.

^1^H, ^13^C{^1^H}) NMR spectra of cyanothiazoles and their complexes were recorded on a Bruker AV400 MHz spectrometer (external standard TMS for ^1^H and ^13^C) at ambient temperature in acetonitrile‐d_3_.

Solid‐state diffuse reflectance spectra were recorded with a Perkin Elmer Lambda 365+ double‐beam UV/Vis spectrometer, equipped with an integrating sphere. Barium sulfate (BaSO_4_) was used as a reference blank for the reflectance spectra in the 200–1100 nm range, with a slit width of 5 nm and a scan speed of 489 nm/min. The Kubelka–Munk transformation was applied to the reflectance data to estimate the energy bandgap (E_g_). Data evaluation was carried out using the UV WinLab Data Processor and Viewer (Version 10.6.2).

Cu K‐edge X‐ray absorption spectra (XAS) were measured at the bending magnet ASTRA beamline at the SOLARIS National Synchrotron Radiation Centre (Kraków, Poland).^[^ ^57^ ^]^ Powder samples were mixed with microcrystalline cellulose, ground with a mortar and pestle, then pressed into thin pellets and put between Kapton tapes. The measurements were performed in transmission mode using an incident photon beam delivered by a modified Lemonnier‐type double‐crystal monochromator equipped with Ge (220) crystals. For the monochromator energy calibration, we used a Cu foil placed in the reference chamber, with the absorption edge at 8979 eV. Positions of the absorption edges were determined based on the maximum of the first derivative of the spectrum. The final spectra were merged from at least three consecutive scans. All spectra were processed using the Athena program from the Demeter software package.^[^ ^58^ ^]^

Solid state photoluminescence spectra have been recorded on a Fluorolog‐3 (Horiba Jobin Yvon, France) spectrophotometer with high‐pressure xenon lamp. The samples were placed in a quartz soldered capillary. The emission spectra were recorded in the range of 450–740 nm with 375 nm excitation wavelength and a resolution of 1 nm. The spectra were measured in a wide temperature range from 6–325 K with 25 K steps provided by the use of a closed‐cycle helium cryostat (ARS Inc.) equipped with LakeShore 331 Temperature Controller. Photoluminescence quantum yields have been determined on a Fluorolog‐3 (Horiba Jobin Yvon, France) spectrophotometer equipped with the Quanta‐φ integration sphere in fused alumina holders. The system provided absolute values of quantum yields via integration of absorption and emission profiles using a calibrated photodetector. Samples have been dispersed in pure barium sulfate. Calibration of the system has been performed with pure barium sulfate samples in fused alumina crucibles. CIE1931 color coordinates^[^ ^59^ ^]^ have been calculated using the Chromaticity Diagram tool implemented in OriginPro 2025 software.

Solution absorption spectra have been recorded in 1 cm quartz cells (1 cm optical pathway) on Agilent 8453 single beam diode array spectrophotometer in acetonitrile solutions. Photoluminescence of these solutions has been recorded on FS5 spectrofluorimeter (Edinburgh Instruments, UK).

### 4.5 Crystallography

The crystal structure data for compounds **1** – **3** were collected on an IPDS 2T dual‐beam diffractometer (STOE & Cie GmbH, Darmstadt, Germany) at 120.0(2) K using MoK_α_ (complexes **1** and **2**) and CuK_α_ (complex **3**) radiation from a microfocus X‐ray source (GeniX 3D Mo High Flux, Xenocs, Sassenage, France). Crystals were cooled with a Cryostream 800 open‐flow nitrogen cryostat (Oxford Cryosystems). The crystallographic data are summarized in Table S1. Data collection and image processing for compounds **1**–**3** were carried out using X‐Area 1.75. Intensity data were scaled with LANA (part of X‐Area) to minimize differences in the intensities of symmetry‐equivalent reflections (integration method). The structures were solved using the intrinsic phasing procedure implemented in SHELXT, and all non‐hydrogen atoms were refined with anisotropic displacement parameters by the full matrix least squares method based on F^2^, using the SHELX–2014 program package.^[^ ^60^ ^]^ The Olex^[^ ^61^ ^]^ and WingX^[^ ^62^ ^]^ program suites were employed to prepare the final version of the CIF files. Mercury was used to prepare the figures.^[^ ^63^ ^]^

Hydrogen atoms were refined using an isotropic model with U_iso_(H) values fixed to be 1.2 times U_eq_ of the carbon atoms to which they were attached.

## Supporting Information

Crystal information and analysis: Tables S1–S6 and Figures S1–S2, FTIR spectra: Figures S3–S20, analysis of absorption spectra for **1**–**3**: Figure S21, Band structures and DOS for **1**–**3**: Figures S22–S26, XAS spectroscopy: Figures S27–S29, CIE1931 chromaticity diagrams: Figure S30, Absorption and emission spectra of cyanethiazoles: Figure S31, NMR spectra of cyanethiazoles and complexes **1**–**3**: Figures S32–S44. Deposition Number(s) 2417242 (for 1), 2417243 (for 2), 2417244 (for 3) contain(s) the supplementary crystallographic data for this paper. These data are provided free of charge by the joint Cambridge Crystallographic Data Centre and Fachinformationszentrum Karlsruhe Access Structures service.

## Conflict of Interests

The authors declare no conflict of interest.

## Supporting information



Supporting Information

## Data Availability

The data that support the findings of this study are available in the supplementary material of this article.
