# Supplementary material for: From Donor‐Acceptor Ligands to Smart Coordination Polymers: Cyanothiazole‐Cu(I) Complexes for Multifunctional Electronic Devices
Source: Chemistry. 2025 May 2;31(31):e202500215. doi: 10.1002/chem.202500215 (PMC12133625; doi:10.1002/chem.202500215)
Supplement: Supplementary file 1 — Supporting Information [file CHEM-31-e202500215-s001.pdf]

Supporting information

Cyanothiazole Copper(I) Complexes: Uncharted Materials with Exceptional Optical and Conductive Properties

Karolina Gutmańska[a], Agnieszka Podborska[b], Tomasz Mazur[b], Andrzej Sławek[b], Ramesh Sivasamy[b], Alexey Maximenko[c], Łukasz Orzeł[d], Janusz Oszajca[d], Grażyna Stochel[d], Amarjith V. Dev[e], Vijayakumar Chakkooth[e], Konrad Szaciłowski[b,f]* Anna Dołęga[a]*

[a] *Gdansk University of Technology, Chemical Faculty, Department of Inorganic Chemistry, Narutowicza 11/12, 80-233 Gdańsk, Poland*

[b] *AGH University of Krakow, Academic Centre of Materials and Technology, al. Mickiewicza 30, 30-059 Kraków, Poland*

[c] *National Synchrotron Radiation Centre SOLARIS, Jagiellonian University, ul. Czerwone Maki 98, Kraków 30-392, Poland*

[d] *Jagiellonian University in Krakow, Faculty of Chemistry, Gronostajowa 2, Kraków, 30-387 Krakow Poland*

[e] *Chemical Sciences and Technology Division, CSIR-National Institute for Interdisciplinary Science and Technology (NIIST), Thiruvananthapuram 695 019, India*

[f] *University of the West of England, Unconventional Computing Lab, Bristol BS16 1QY, United Kingdom*

Table S1 Crystal and refinement data for **1** - **3**

Compound	Complex 1	Complex 2	Complex 3
**Empirical formula**	$C_4H_2CuIN_2S$	$C_4H_2CuIN_2S$	$C_4H_2CuIN_2S$
**Formula weight (g mol^{-1})**	300.58	300.58	300.58
**Wavelength (Å)**	0.71073	0.71073	1.54186
**Temperature (K)**	120(2)	120(2)	120(2)
**Crystal system**	triclinic	triclinic	triclinic
**Space group**	*P* -1	*P* -1	*P* -1
***a* (Å)**	4.1231(4)	6.6261(6)	6.4835(7)
***b* (Å)**	6.9179(6)	7.6830(7)	7.5760(9)
***c* (Å)**	12.9182(12)	8.1333(8)	8.3822(9)
***α* (°)**	92.993(7)	115.757(7)	114.401(8)
***β* (°)**	93.993(7)	108.542(7)	107.399(8)
***γ* (°)**	95.290(7)	91.328(8)	91.821(9)
**Volume (Å^3)**	365.37(6)	347.19(6)	352.03(7)
Z	2	2	2
**Calculated density (g cm^{-3})**	2.732	2.875	2.836
**Crystal size (mm)**	0.188x 0.087x 0.031	0.178x 0.124x 0.05	0.214x 0.144x 0.069
**Absorption coefficient (mm^{-1})**	7.405	7.793	40.82
***F*(000)**	276	276	276
**θ range (°)**	2.962 to 29.159	2.986 to 29.164	6.172 to 67.131
**Limiting indices**	-5≤h≤5	-9≤h≤9	-7≤h≤7
	-9≤k≤9	-10≤k≤10	-8≤k≤8
	-17≤l≤17	-11≤l≤11	-9≤l≤9
**Reflections collected / unique/unique [*I*>2*σ*(*I*)]**	4363, 1976, 1808	4680, 1864, 1803	2650, 1151, 1143
**R_{int}**	0.0228	0.0192	0.0411
**Completeness to $θ_{max}$ (%)**	99.2	99.6	91.7
**Data / restraints / parameters**	1976 / 0 / 82	1864 / 0 / 82	1151 / 0 / 82
**Goodness-of-fit on F^2**	1.048	1.074	1.122
**Final R indices [*I*>2*σ*(*I*)]**	R_1 = 0.0288	R_1 = 0.0188	R_1 = 0.0599
	wR_2 = 0.0751	wR_2 = 0.0491	wR_2 = 0.1725
**R indices (all data)**	R_1 = 0.0324	R_1 = 0.0195	R_1 = 0.0601
	wR_2 = 0.0774	wR_2 = 0.0496	wR_2 = 0.1727
**Largest diff. peak and hole (e Å^{-3})**	1.001/-1.148	0.966/-0.67	1.729/-1.012,
**CCDC deposition number**	2417242	2417243	2417244

Table S2 Selected bond lengths and short contacts in angstroms [Å] in complexes **1** - **3**

Bonds [Å]/Compound	Complex 1	Complex 2	Complex 3
**Cu1—I1/I1$^{i/ii/iii/vi}$**	2.6177(5)/2.6372(6)/2.6390(6)	2.6709(4)/2.6357(4)	2.6428(18)/ 2.6776(18)^{i}
**Cu1—N1/N2$^{i/vii}$**	2.050(3)	2.0647(18)/1.943(2)	2.072(11)/1.949(10)^{i}
**S1—C1/C3/C2**	1.707(4)/-/1.367(6)	1.717(2) /1.701(2)	1.701(12)/1.698(12)^{i}
**N1—C1/C2/C3**	1.311(5)/ -/1.366(5)	1.324(3)/1.369(3)	1.290(14)/1.372(14)^{i}
**N2—C4**	1.143(6)	1.148(3)	1.151(15)
**C2—C3/C4**	1.367(6)/1.425(6)	1.368(3)	1.389(17)/1.434(15)^{i}
**Cu1—Cu1^{iv}/ Cu1^{v}**	2.7460(10)/2.7503(10)	-	-
**C4—C1**	-	1.423(3)	-
**Cu···Cu$^{ii/vi}$**	-	3.173	3.083
**S···I$^{vii/ix}$**	-	3.714	3.632/3.665

Symmetry operations: ^{i}: 2-x, 1-y, 2-z; ^{ii}: 1+x, y, z; ^{iii}: 1-x,1-y, -z; ^{iv}: 2-x,1-y, -z; ^{v}:1-x, 1-y, -z; ^{vi}:2-x,2-y,2-z; ^{vii}:2-x,1-y,1-z; ^{viii}:-1+x, -1+y, -1+z; ^{ix}: 1-x, 1-y, 2-z

Table S3 Selected angles in degrees [°] in complexes **1** - **3**

**Angles [°]/Compound**	**Complex 1**	**Complex 2**	**Complex 3**
**N1—C1—S1**	115.5(3)	115.1(2)	117(1)
**C3—C2—C4**	126.8(4)	124(1)	124(1)
**C3—C2—S1**	110.7(3)	110.7(2)	109(1)
**C4—C2/C1—S1**	122.4(3)	122.6(2)	-
**C2/C3/C4—C1/C2/C3—N1**	114.1(4)	115.1(2)/122.2(2)	116(1)/119(1)
**C1/C2/C4—C2/C4—N2**	179.5(6)	176.5(3)	178(1)
**I1/I1$^{i/ii/iv}$—Cu1—N1/N1^{vi}/N2^{vii}**	109.0(1)/104.72	101.59(6)/103.52(6)/ 110.82(7)/118.49(7)	102.9(3)/114.8(3)/ 101.7(3)/112.3(3)
**N1—Cu1—Cu1$^{iii/iv}$**	123.8(1)/ 119.3(1)	-	-
**I1/I1$^{iii/v/vii/viii}$—Cu1/Cu1$^{iii/iiiv}$—Cu1/Cu1$^{v/viii}$**	58.89(1)/58.61(1)/58.13(1)/ 131.12(2)/58.55(1)/131.24(2)	-	-
**I1/I1^{ii}—Cu1/Cu1$^{i/iv/v}$—I1/I1$^{i/v/vi}$**	103.37(2)/117.16(2)/117.02(2)	106.55(1)	109.18(7)/109.18(7)
**Cu1^{iv}—Cu1—Cu1^{v}**	97.21(2)	-	-
**Cu1—I1/I1$^{i/ii/iv}$—Cu1$^{i/iv/v/vi/vii}$**	62.84(2)/62.98(2)/103.37(2)	73.45(1)/73.45(1)	70.82(6)/70.82(6)
**C1—N1—C2/C3**	111.1(3)	109.9(2)	109(1)
**C1/C2/C3/C4—N1/N2—Cu1/Cu$^{v/vii}$**	122.4(3)/126.5(3)	129.7(2)/119.7(2)/ 168.3(2)	120.0(9)/130.9(9)/ 170(1)
**C1—S1—C2/C3**	88.6(2)	89.2(1)	89.6(6)
**N1—Cu1—N2**	-	114.22(9)	114.8(4)

Symmetry operations: ^{i}: 2-x, 1-y, 2-z; ^{ii}: 1+x, y, z; ^{iii}: 1-x,1-y, -z; ^{iv}: 2-x,1-y, -z; ^{v}:1-x, 1-y, -z; ^{vi}:2-x,2-y,2-z; ^{vii}:2-x,1-y,1-z; ^{vii}: -1+x,y,z

Table S4 π···π stacking interactions in complexes **1** - **3**

**Compound**	**π···π stacking interactions**	**Distances between Cg-Cg [Å]**	**Cg definition**
1	Cg$_{Tz1}$···Cg$_{Tz2}$	4.123	Cg$_{Tz1}$: N1–C1–S1–C2–C3<br>Cg$_{Tz2}$: N1i–C1i–S1i–C2i–C3i
2	Cg$_{Tz3}$···Cg$_{Tz4}$	3.783	Cg$_{Tz1}$: N1–C3–C2–S1–C1<br>Cg$_{Tz2}$: N1^{ii}–C3^{ii}–C2^{ii}–S1^{ii}–C1^{ii}
3	Cg$_{Tz4}$···Cg$_{Tz5}$	3.882	Cg$_{Tz1}$: N1–C2–C3–S1–C1<br>Cg$_{Tz2}$: N1^{ii}–C2^{ii}–C3^{ii}–S1^{ii}–C1^{ii}

Cg$_{Tz}$ - centroid of the thiazole ring, Symmetry operations: ^{i}:-1+x,y,z; ^{ii}:1-x,1-y,1-z

Table S5 Geometrical parameters of C–H···X hydrogen bonds in complexes **1** – **3**

**Compound**	bond	***D***···***A*** **[Å]**	**H**···***A*** **[Å]**	***D***–**H [Å]**	***ΔDHA*** **[°]**	**Type of interaction**
1	C1–H1···I1^{i}	3.749(4)	3.0588	0.950	130.8	
	C3–H3···N2^{ii}	3.413(7)	2.558	0.950	140.0	
2	C3–H3···I1^{iii}	3.793(2)	3.1721	0.950	124.6	
	C2–H2···I1^{iii}	3.776(2)	3.1677	0.950	123.5	intermolecular
	C2–H2···I1^{iv}	3.753(2)	3.1364	0.950	124.2	
3	C1–H1···I1^{v}	3.71(2)	3.1321	0.95	120.89	
	C3–H3···I1^{vi}	3.81(2)	3.1005	0.95	129.95	

Symmetry operations: ^{i}: 1-x, -1+y, z; ^{ii}: 1-x, 1-y, 1-z; iii:-1+x, y,z; ^{iv}:1-z,2-y, 1-z; ^{v}:1-x,1-y,2-z; ^{vi}:-1+x,y, -1+z

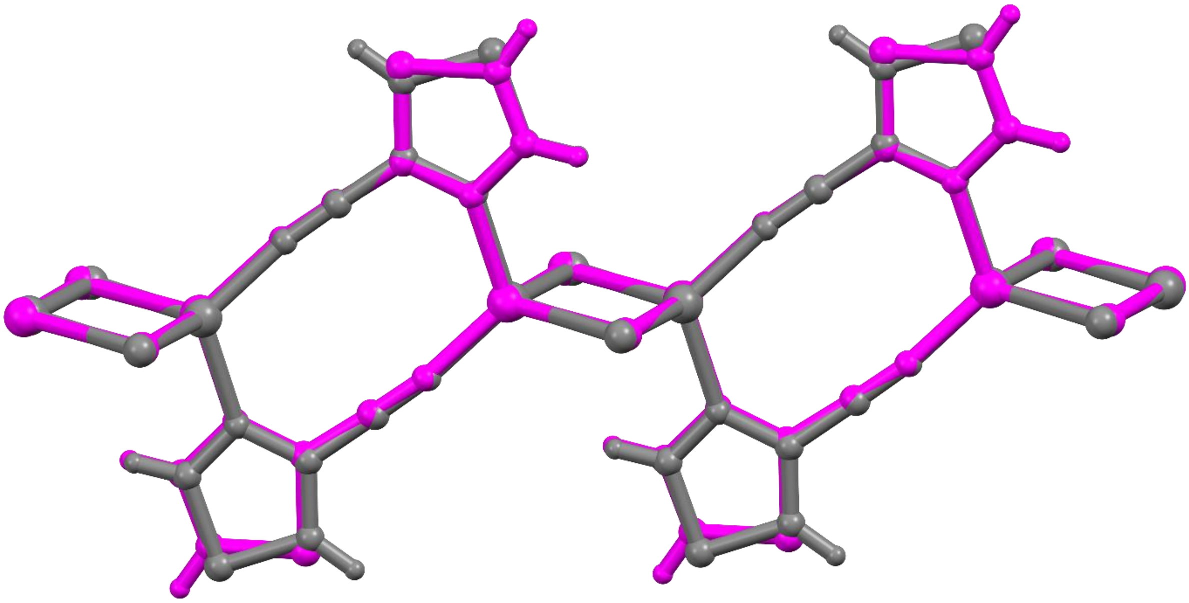

Figure S1. The overlay of molecular structures of **2** (magenta) and **3** (grey) illustrating the similar size of both rings in the studied complexes. The overlay prepared in the program Mercury.

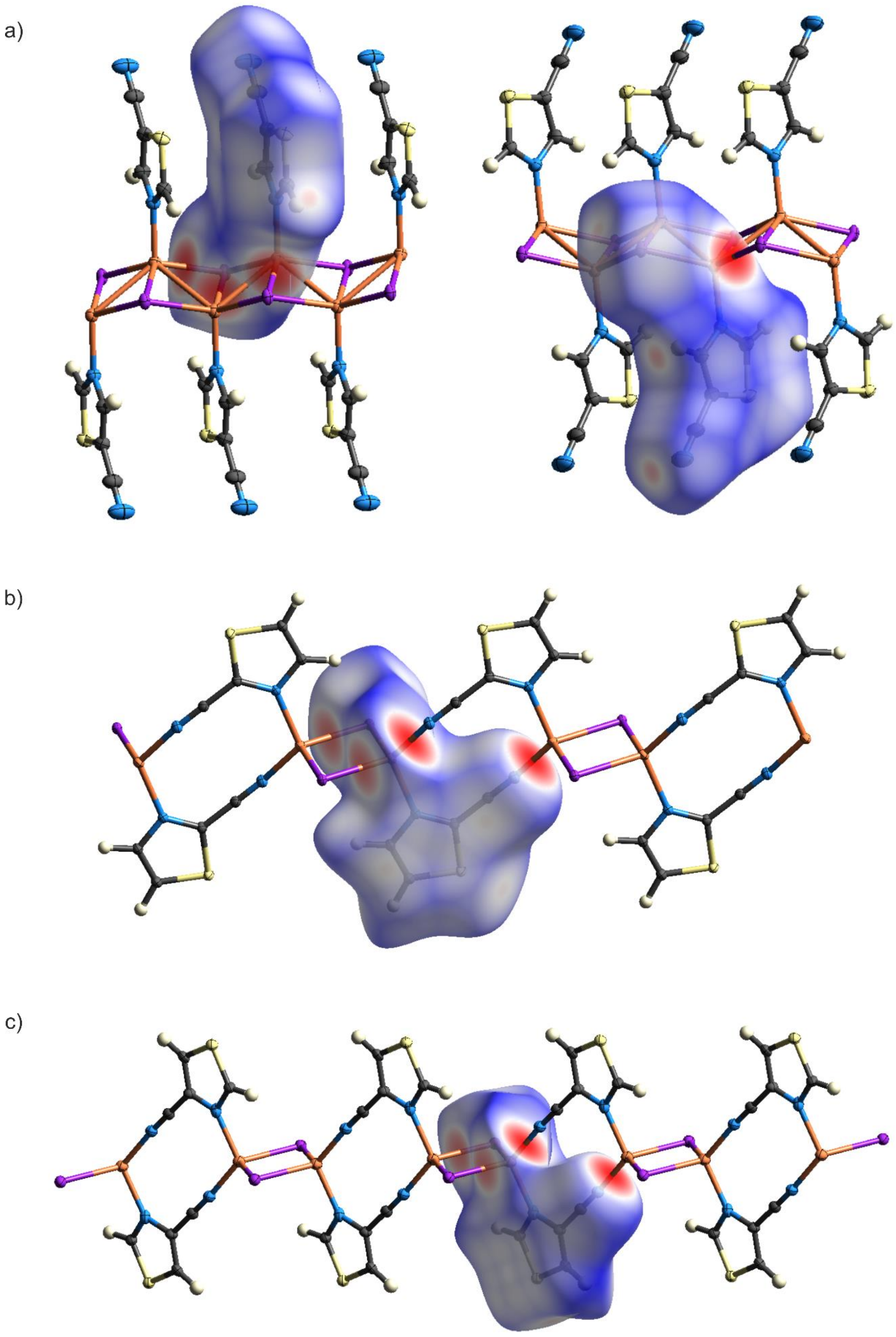

Figure S2. The fragments of **1** - **3** selected for the calculation of Hirshfeld surfaces and HSs: a) two views of the HS of complex **1**; b) HS of complex **2**; c) HS of complex **3**.

Table S6 The fingerprint plots for specific intermolecular interactions in: a) **1**, b) **2** and c) **3**.

**Compound 1**	**Compound 2**	**Compound 3**
**a)** C-H···I(H···I)		
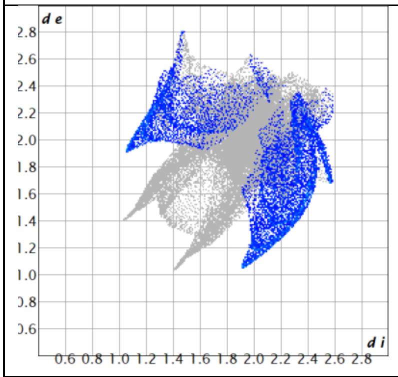	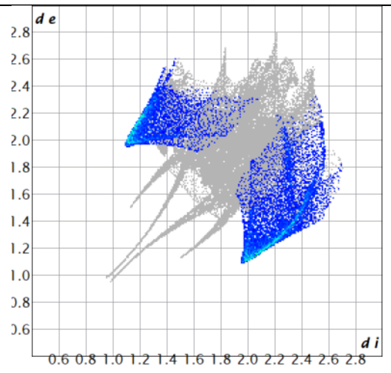	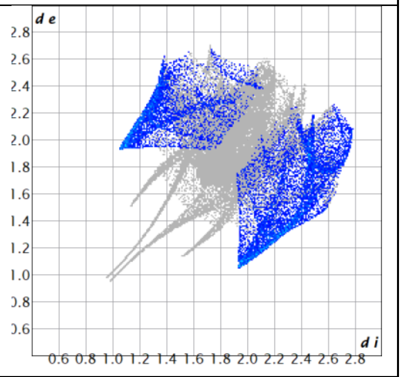
b) C-H···N		
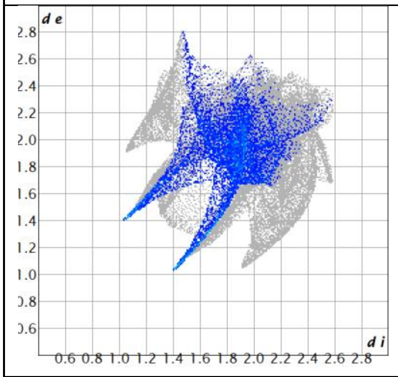	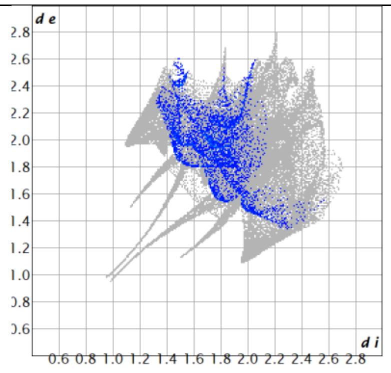	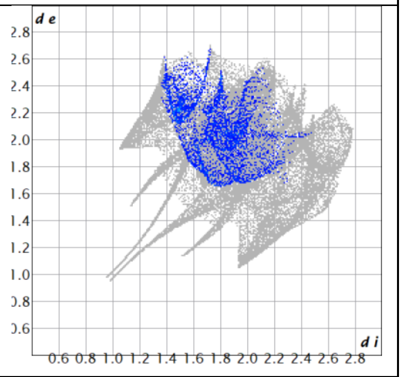
c) I···S		
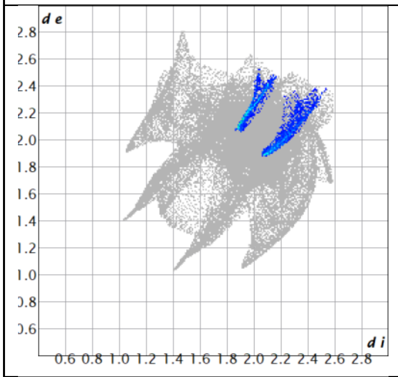	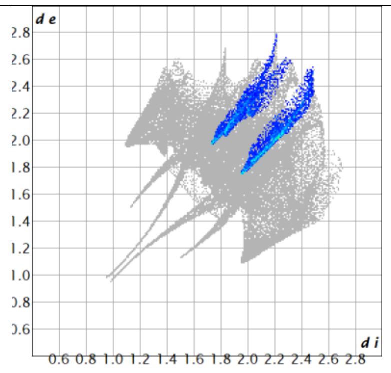	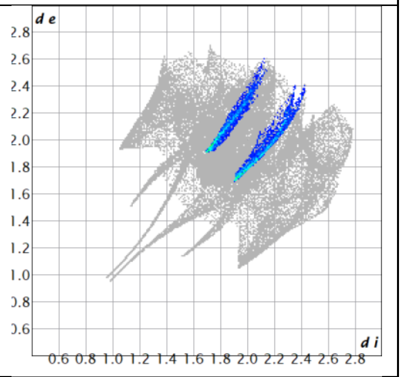
d) C···H		
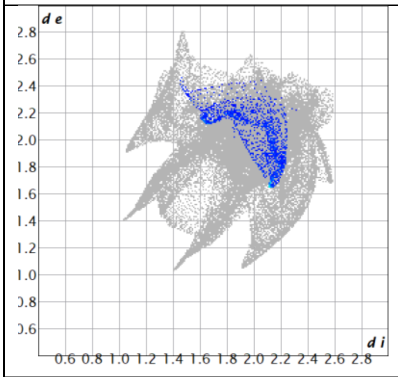	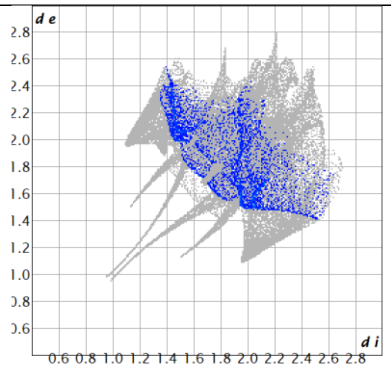	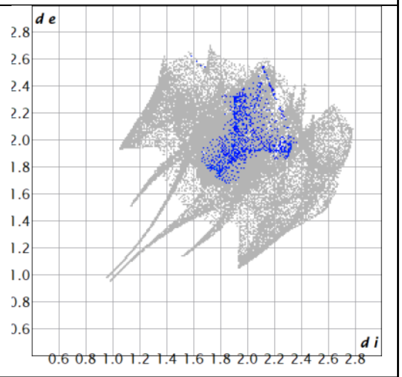
e) C···C		

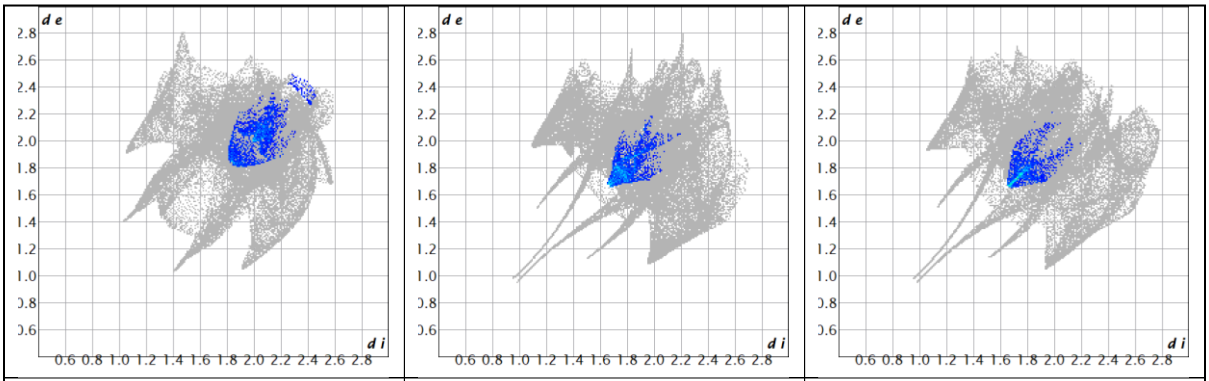
d e
d i
2.8 2.6 2.4 2.2 2.0 1.8 1.6 1.4 1.2 1.0 0.8 0.6
0.6 0.8 1.0 1.2 1.4 1.6 1.8 2.0 2.2 2.4 2.6 2.8

f) N···C

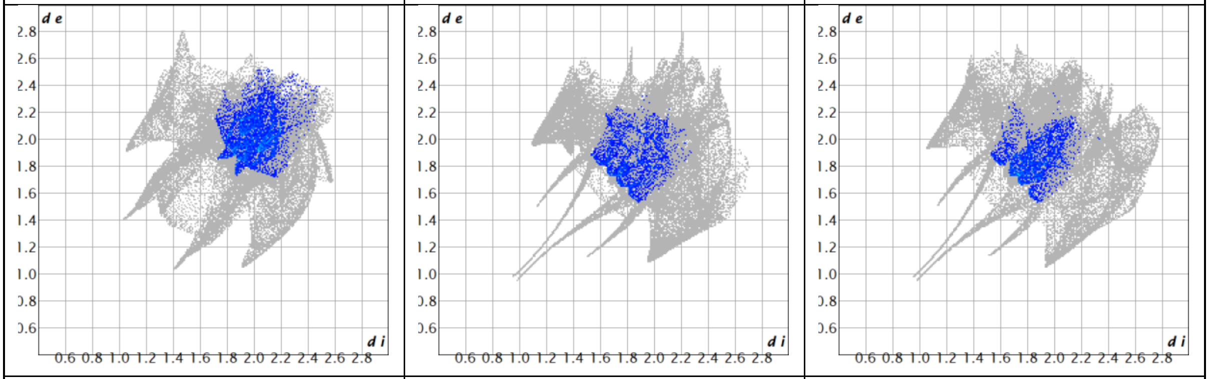
d e
d i
2.8 2.6 2.4 2.2 2.0 1.8 1.6 1.4 1.2 1.0 0.8 0.6
0.6 0.8 1.0 1.2 1.4 1.6 1.8 2.0 2.2 2.4 2.6 2.8

g) S···C

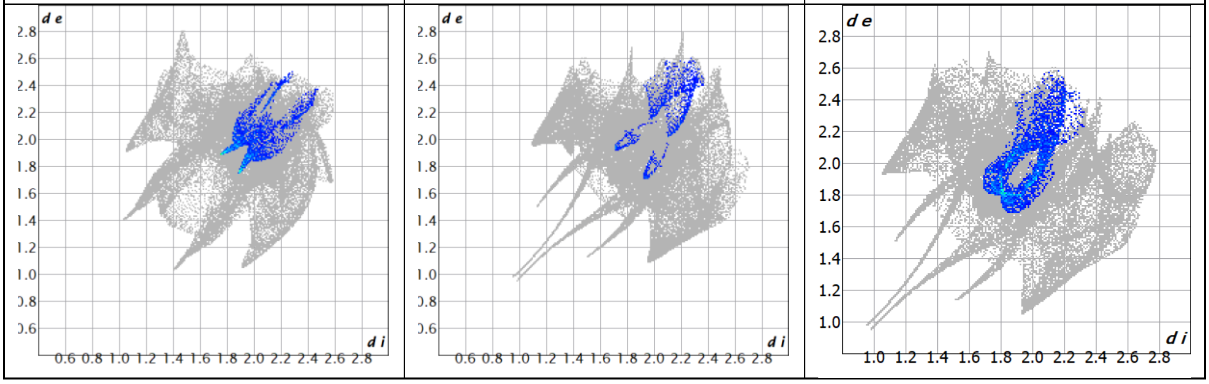
d e
d i
2.8 2.6 2.4 2.2 2.0 1.8 1.6 1.4 1.2 1.0 0.8 0.6
0.6 0.8 1.0 1.2 1.4 1.6 1.8 2.0 2.2 2.4 2.6 2.8
1.0 1.2 1.4 1.6 1.8 2.0 2.2 2.4 2.6 2.8

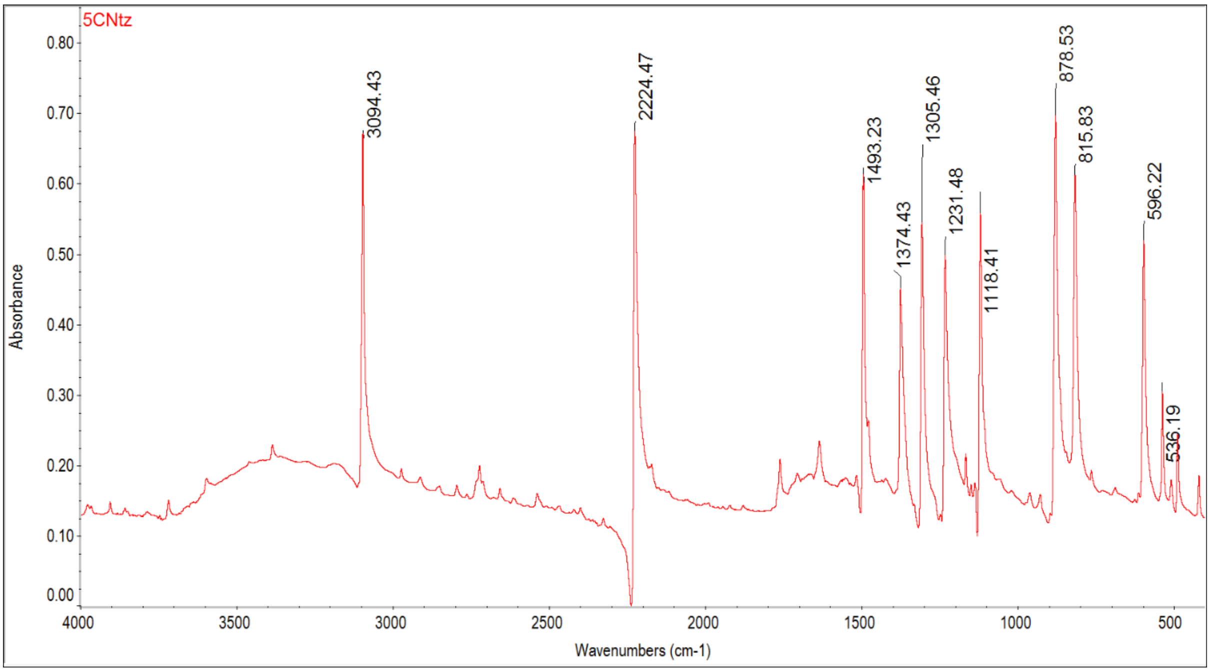

Figure S3. FTIR spectra of 5-cyanothiazole.

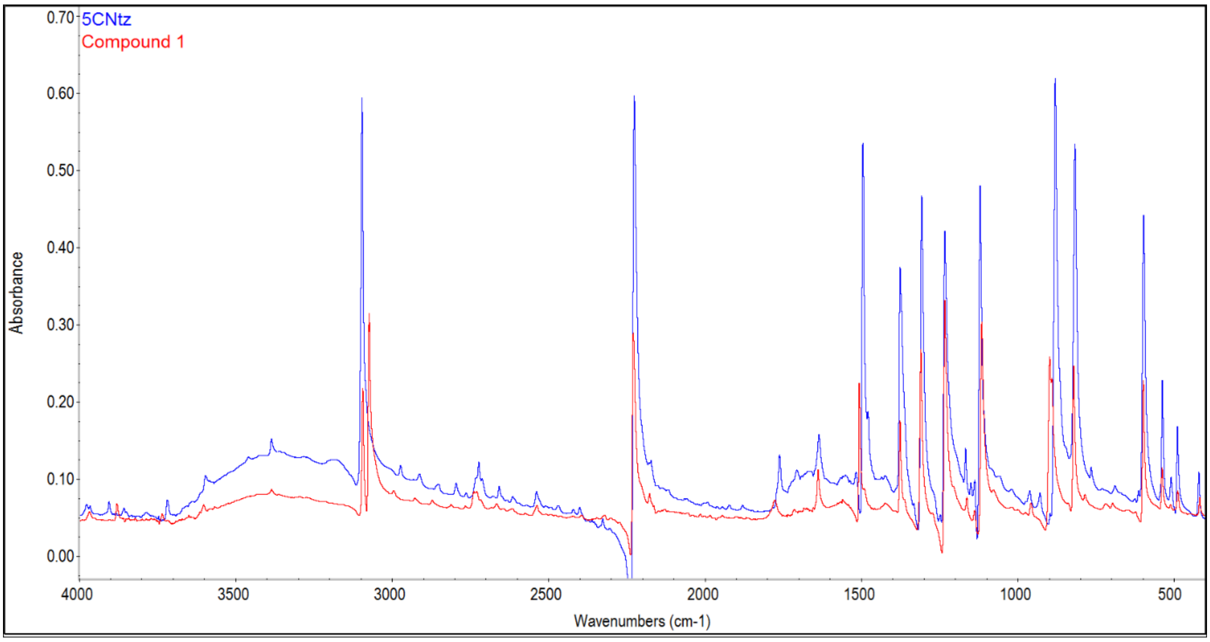

Figure S4. Superimposed FTIR spectra of compound **1** and **5CNtz**.

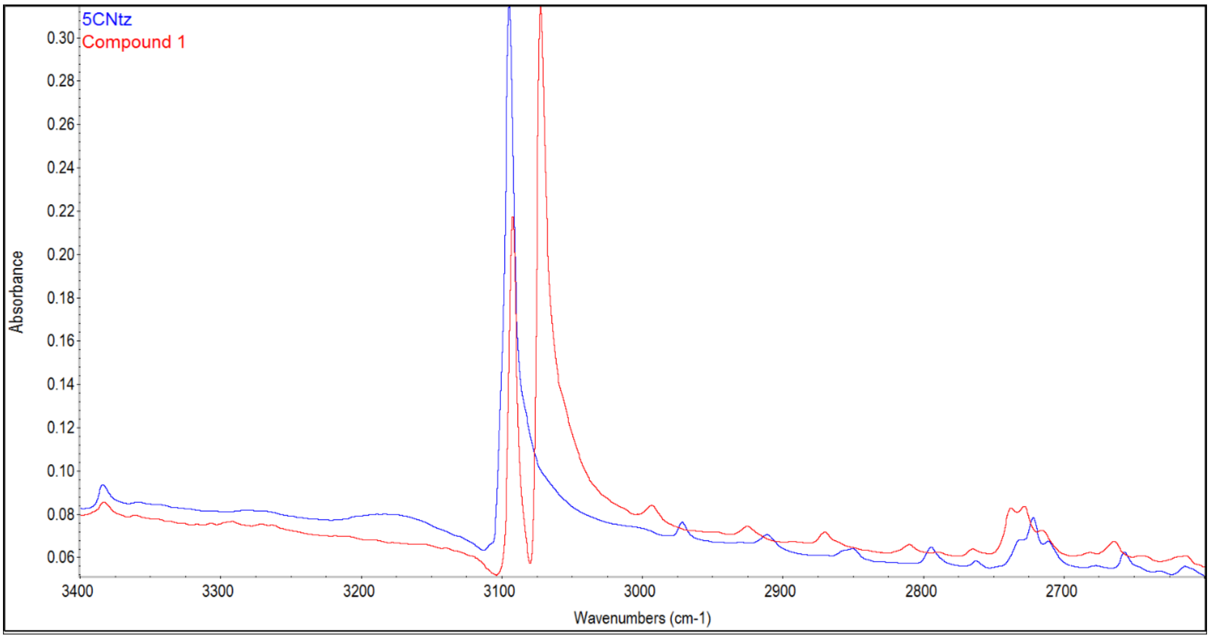

Figure S5 Superimposed FTIR spectra of compound **1** and **5CNtz** in the range of 3400-2600 cm^{-1}.

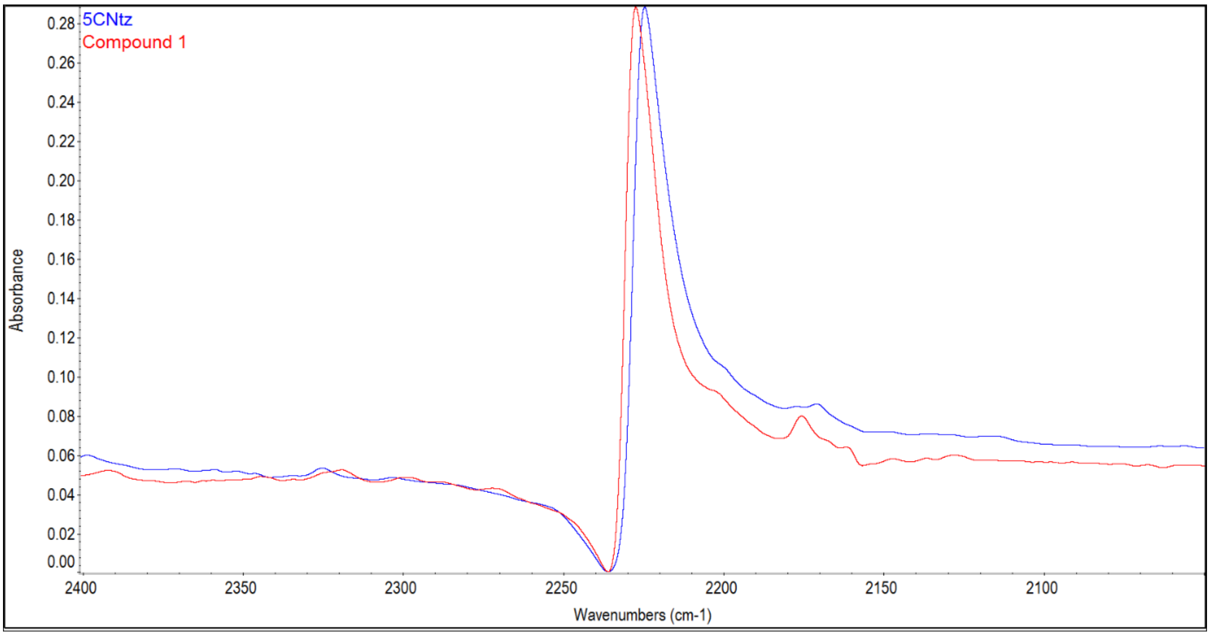

Figure S6 Superimposed FTIR spectra of compound **1** and **5CNtz** at the range of 2400-2050 cm^{-1}.

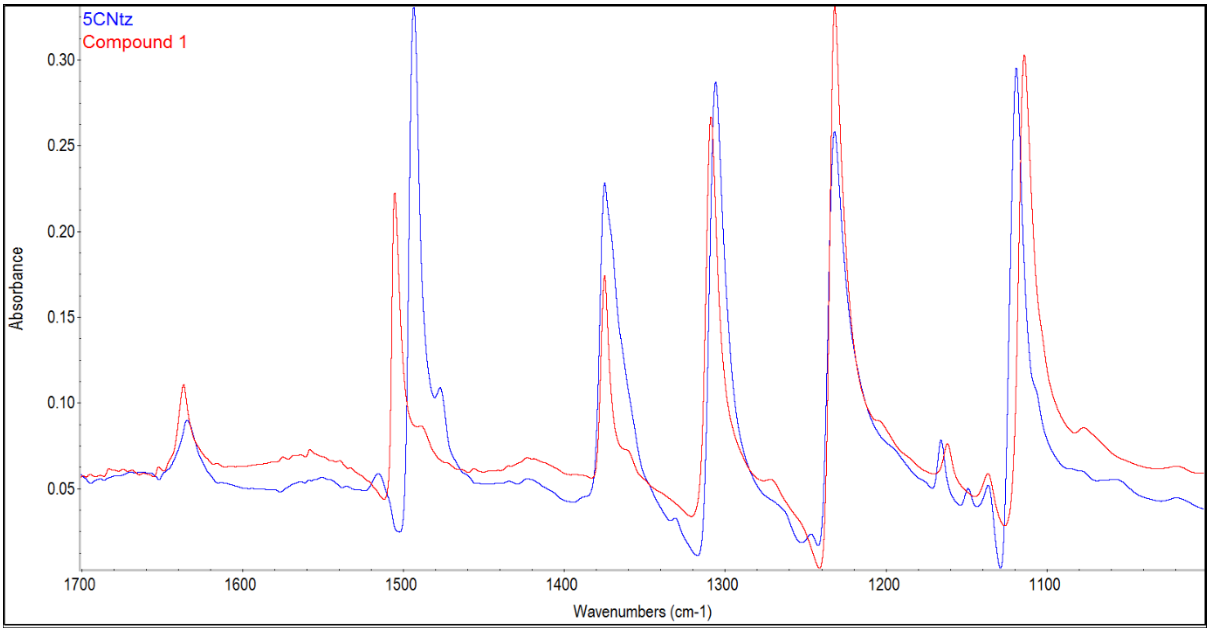

Figure S7 Superimposed FTIR spectra of compound **1** and **5CNtz** in the range of 1700-1000 cm^{-1}.

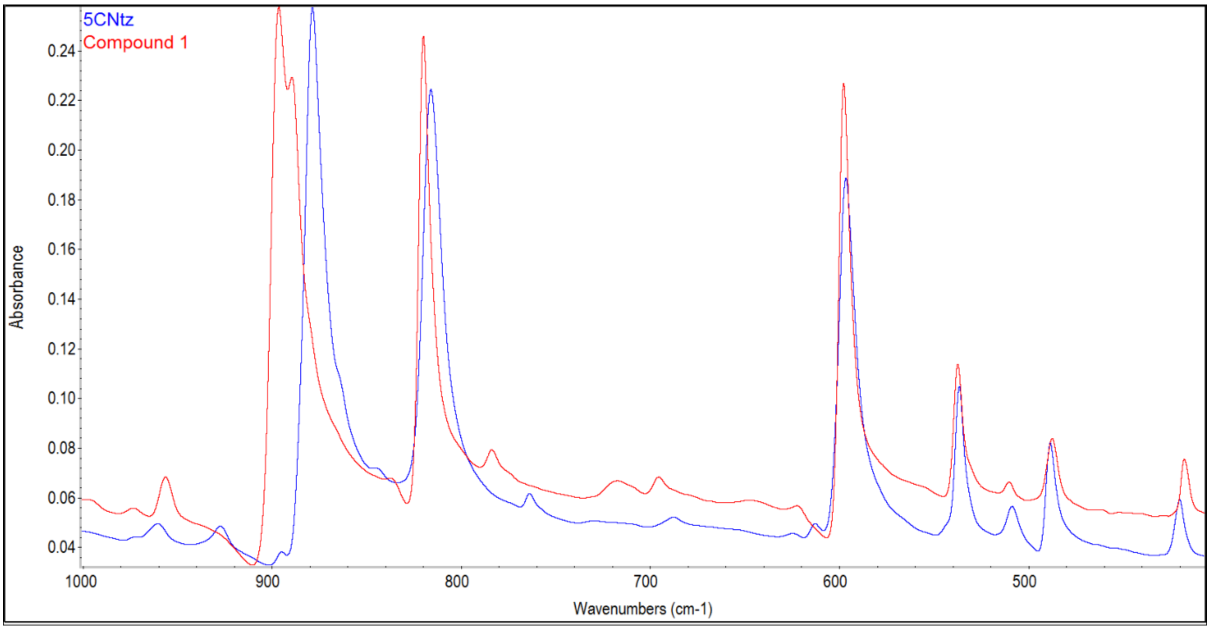

Figure S8 Superimposed FTIR spectra of compound **1** and **5CNtz** in the range of 1000-400 cm^{-1}.

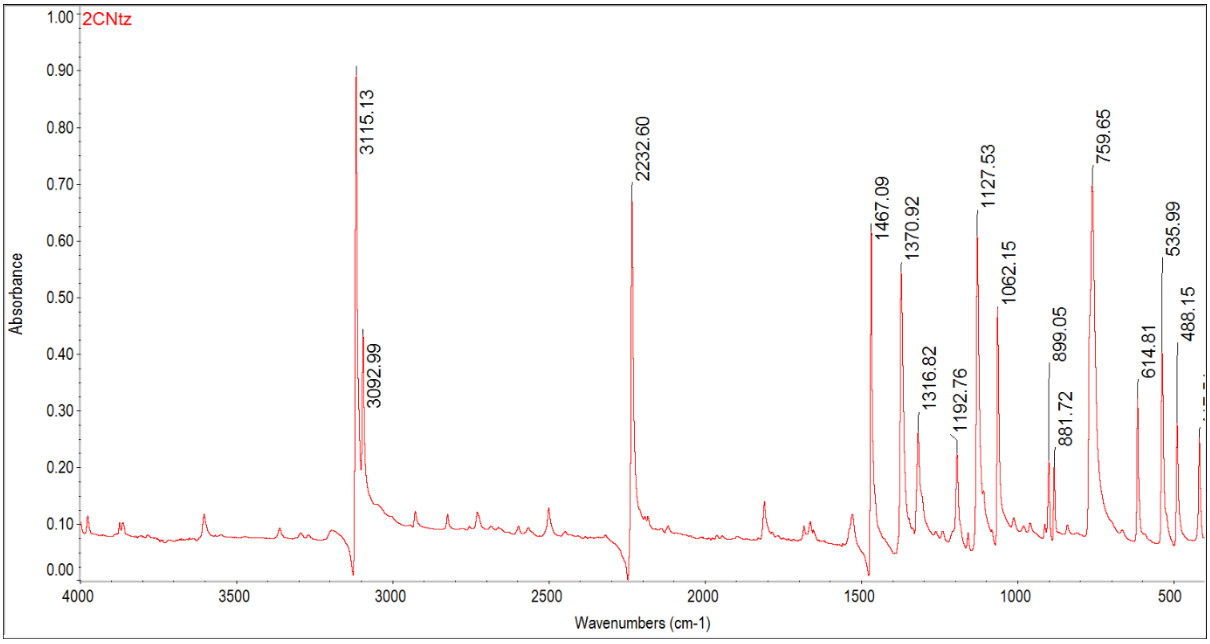

Figure S9. FTIR spectra of 2-cyanothiazole.

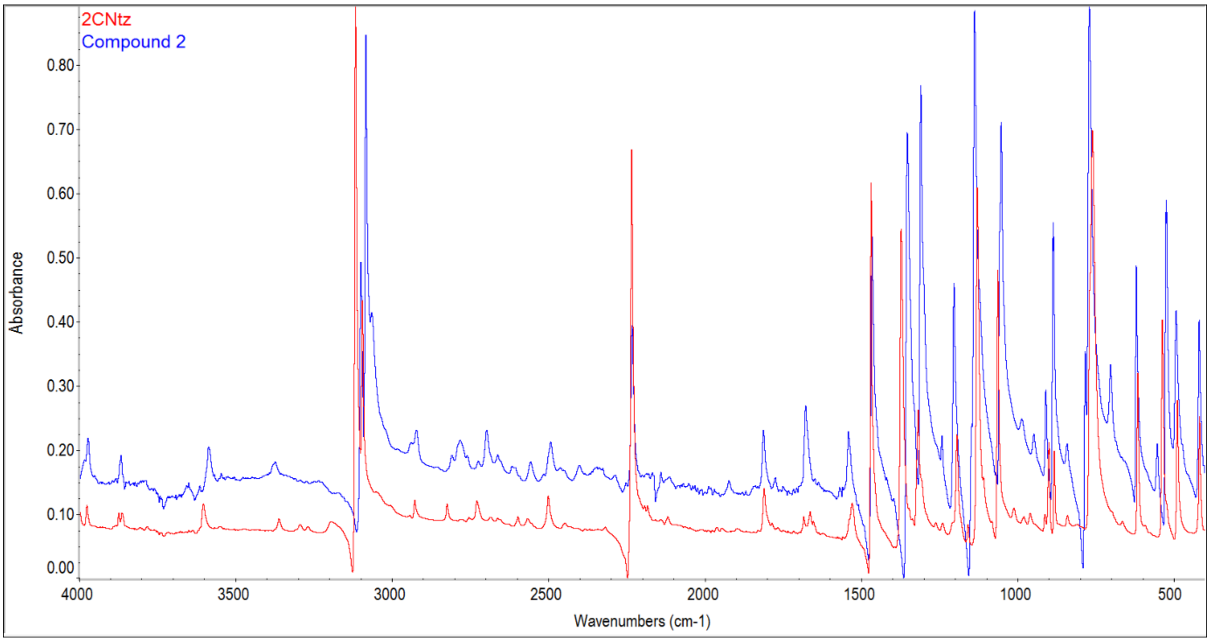

Figure S10. Superimposed FTIR spectra of compound **2** and **2CNtz**.

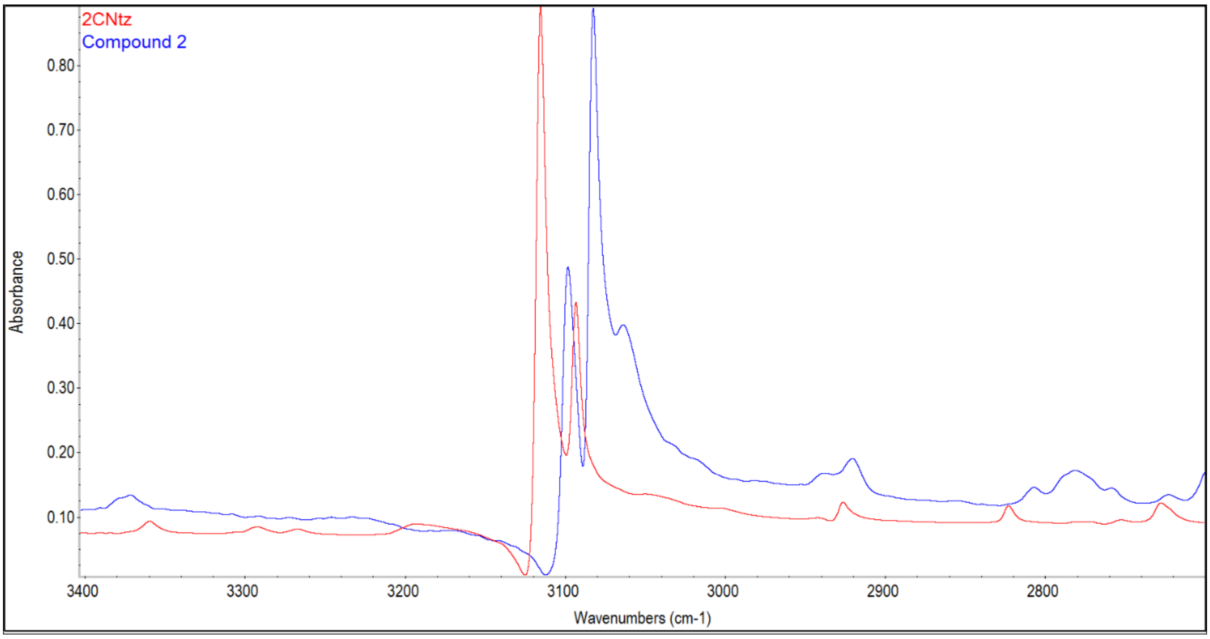

Figure S11. Superimposed FTIR spectra of compound **2** and **2CNtz** in the range of 3400-2700 cm^{-1}.

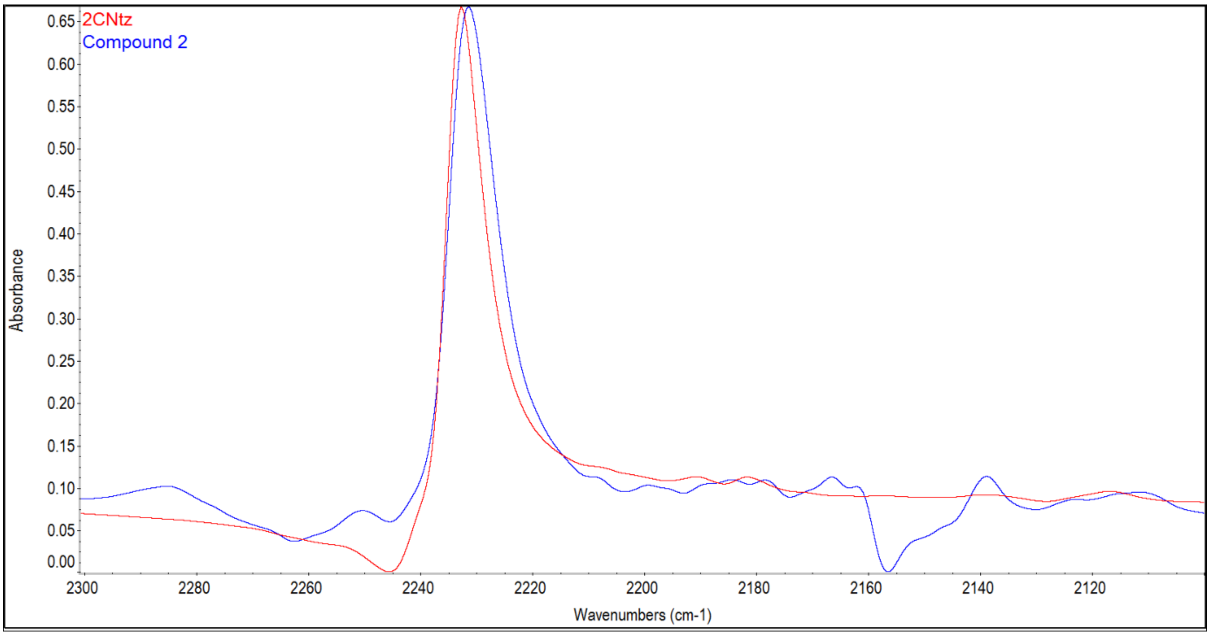

Figure S12. Superimposed FTIR spectra of compound **2** and **2CNtz** in the range of 2300-2100 cm^{-1}.

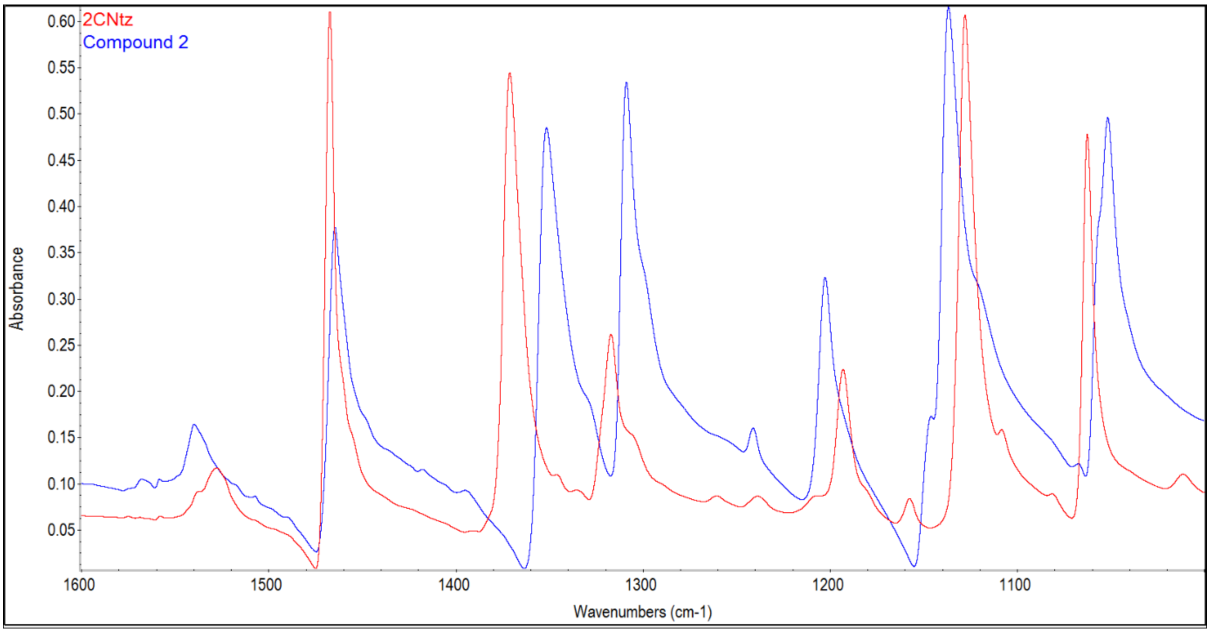

Figure S13. Superimposed FTIR spectra of compound **2** and **2CNtz** in the range of 1600-1000 cm^{-1}.

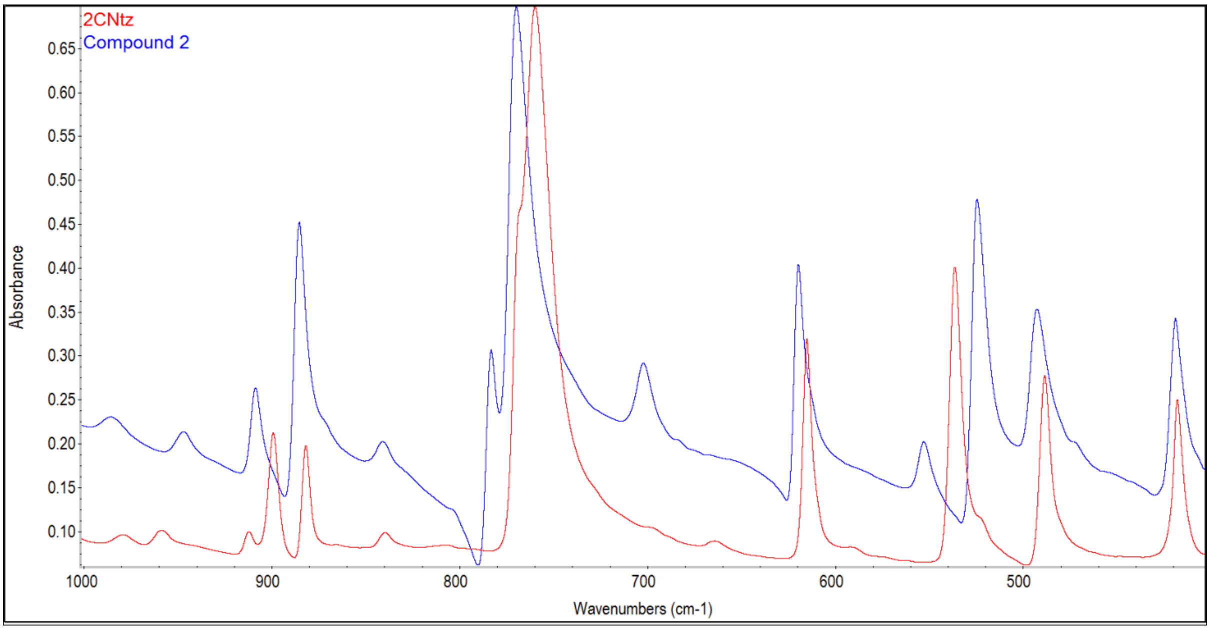

Figure S14. Superimposed FTIR spectra of compound **2** and **2CNtz** in the range of 1000-400 cm^{-1}.

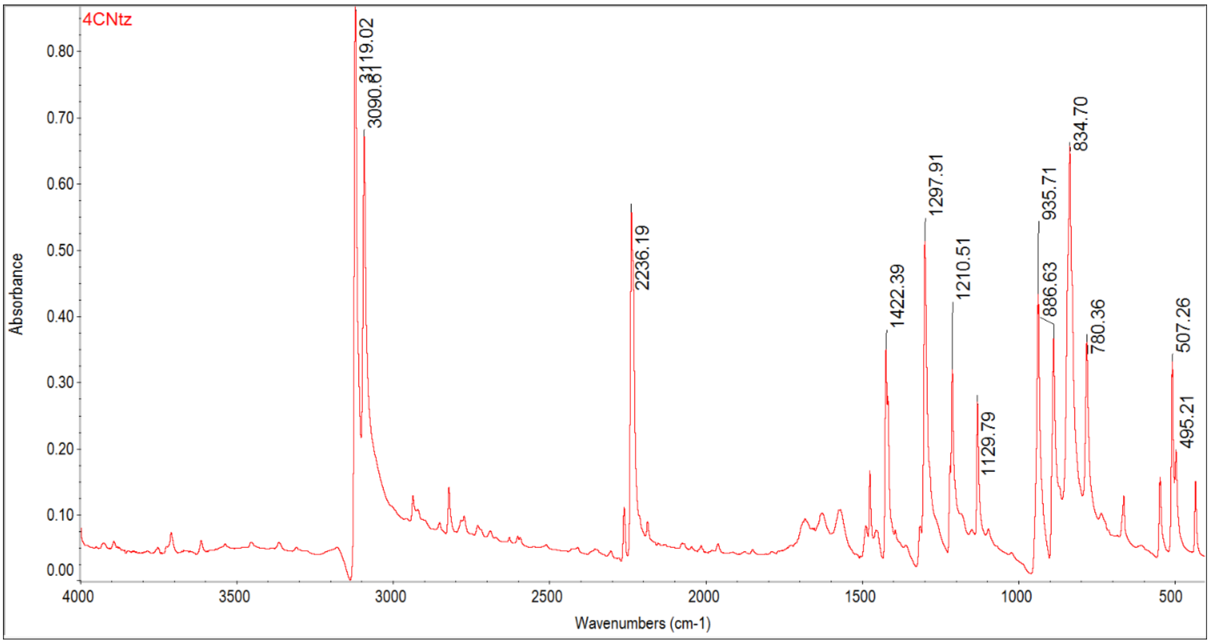

Figure S15. FTIR spectra of 4-cyanothiazole.

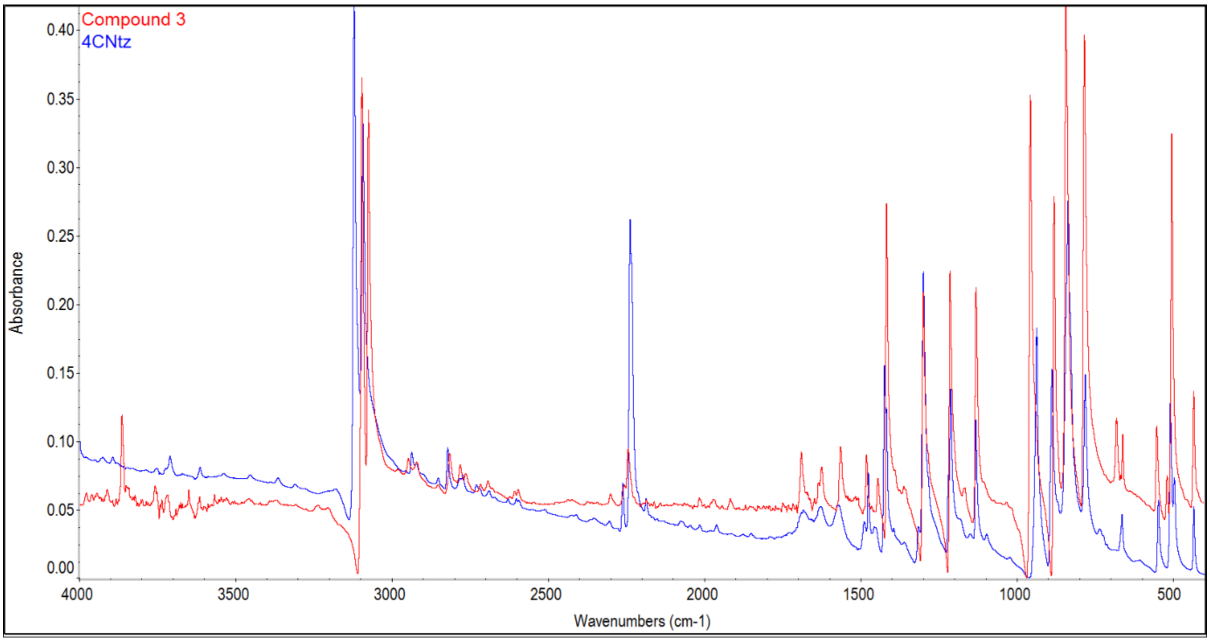

Figure S16. Superimposed FTIR spectra of compound **4** and **4CNtz**.

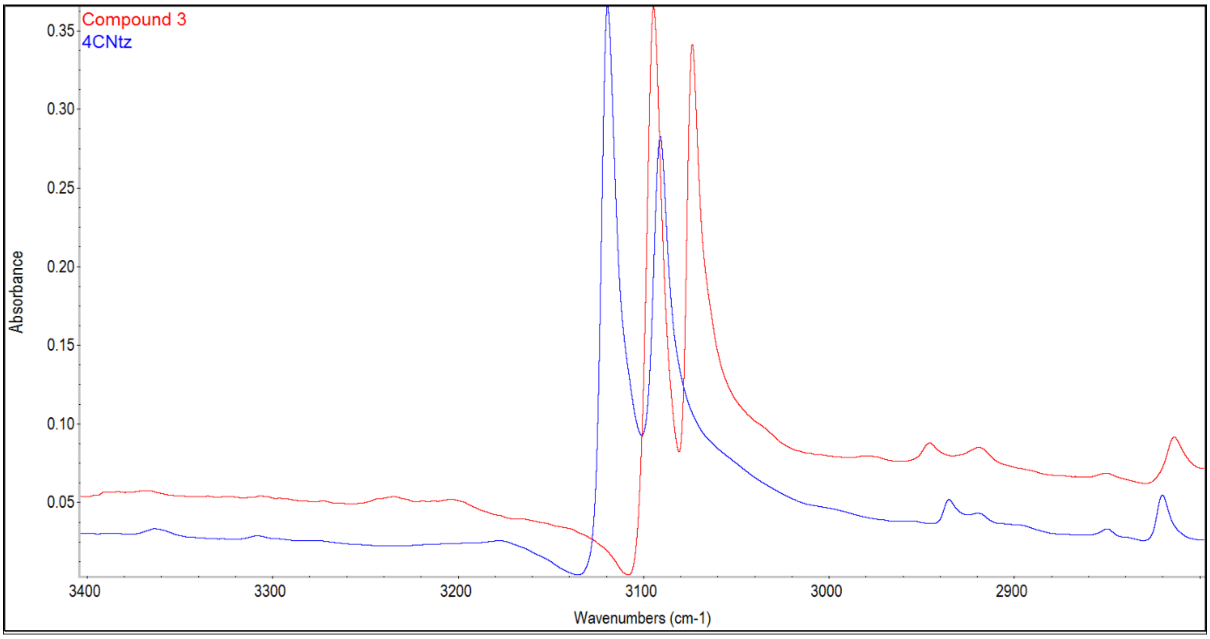

Figure S17. Superimposed FTIR spectra of compound **3** and **4CNtz** in the range of 3400-2800 cm^{-1}.

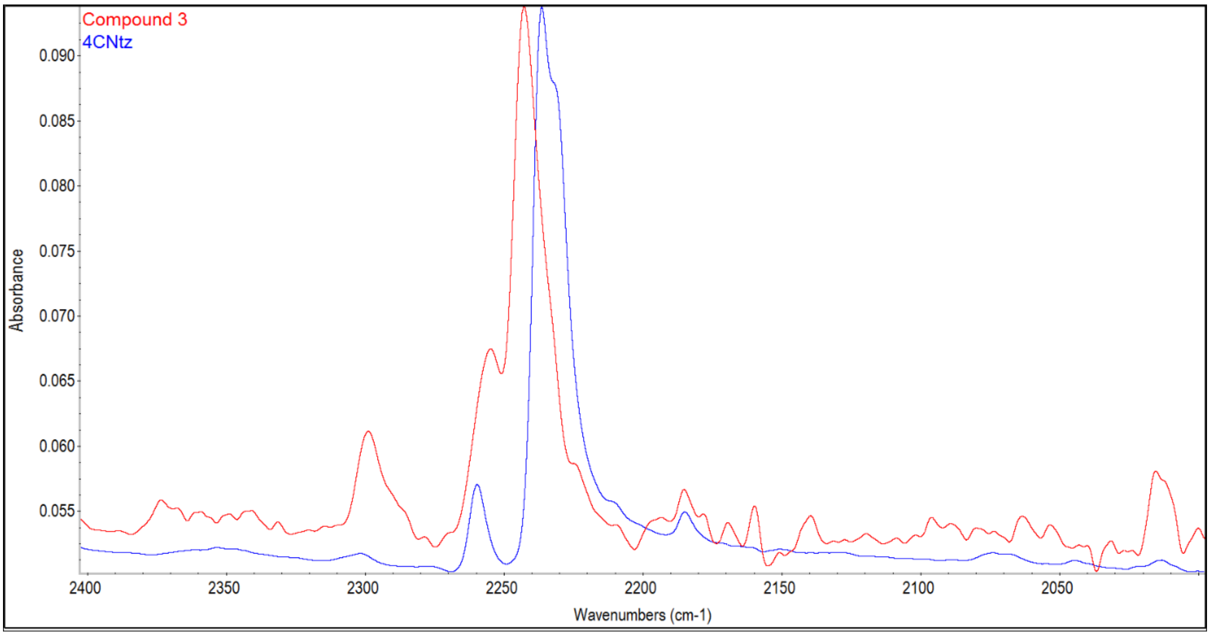

Figure S18. Superimposed FTIR spectra of compound **3** and **4CNtz** at the range of 2400-2000 cm^{-1}.

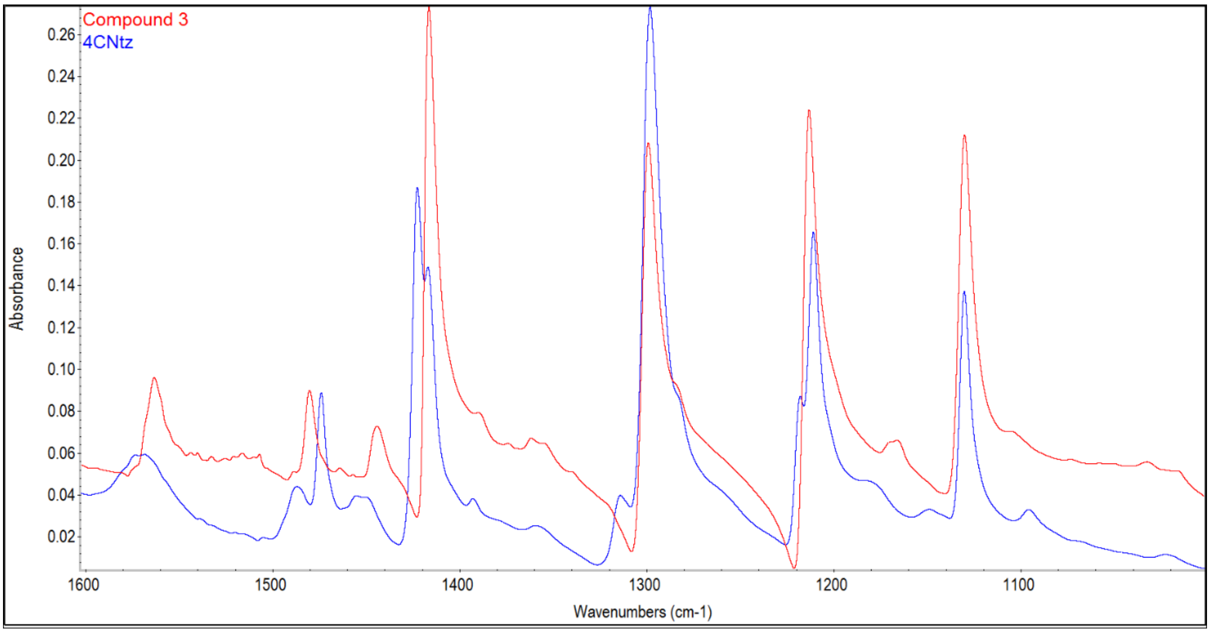

Figure S19. Superimposed FTIR spectra of compound **3** and **4CNtz** in the range of 1600-1000 cm^{-1}.

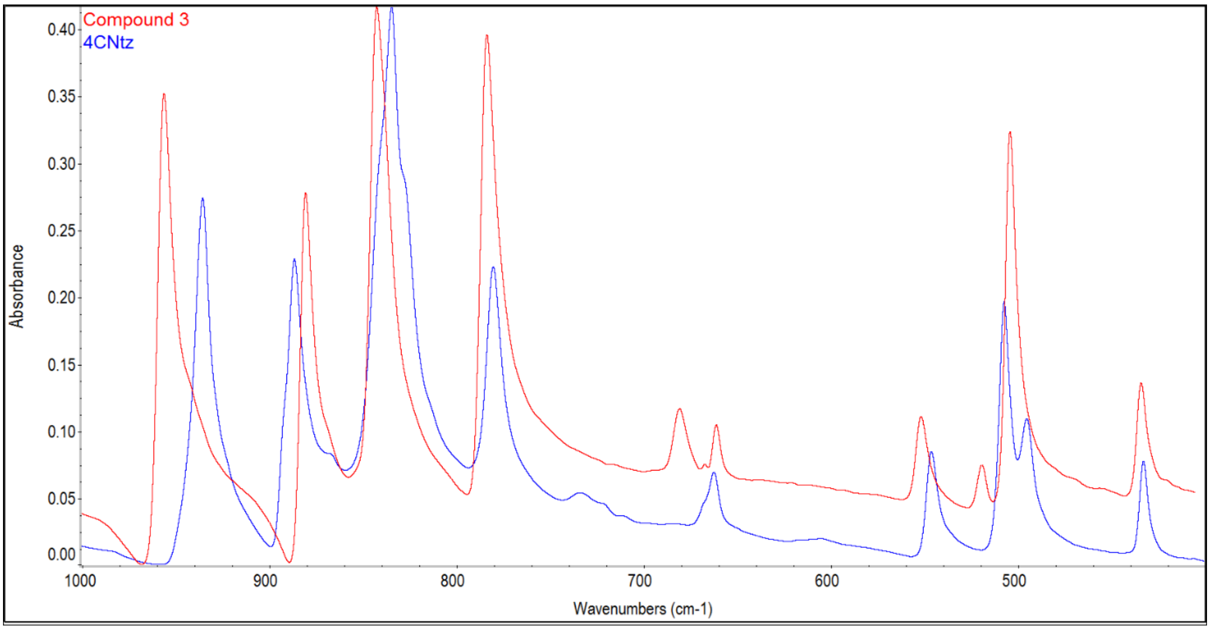

Figure S20. Superimposed FTIR spectra of compound **3** and **4CNtz** in the range of 1000-400 cm^{-1}.

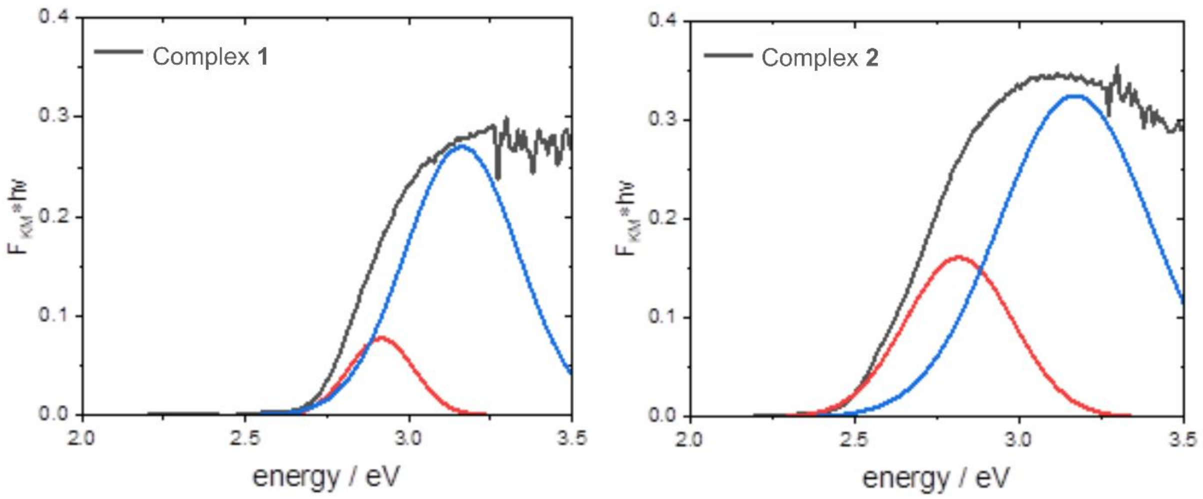

Figure S21. Deconvolution of absorption spectra of complexes **1** and **2** into Gaussian components.

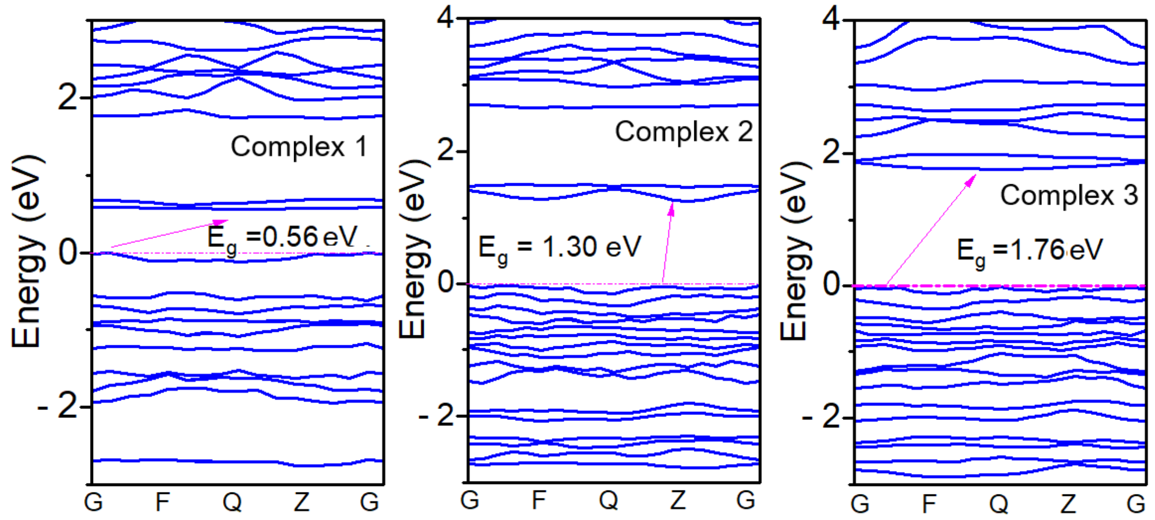

Figure S22. Band structure and density of states for complex **1** (**5CNtz**-CuI), complex **2** (**2CNtz**-CuI) and complex **3** (**4CNtz**CuI). (DFT-MBD+SOC)

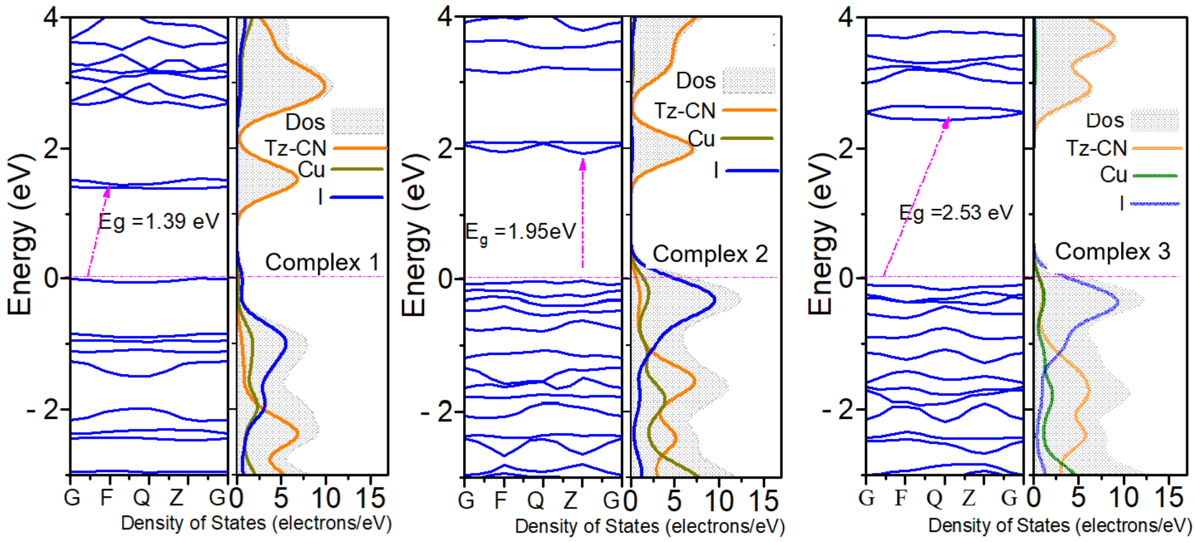

Figure S23. Band structure and density of states for complex **1** (**5CNtz**-CuI), complex **2** (**2CNtz**-CuI) and complex **3** (**4CNtz**-CuI). (DFT-MBD+U7.5)

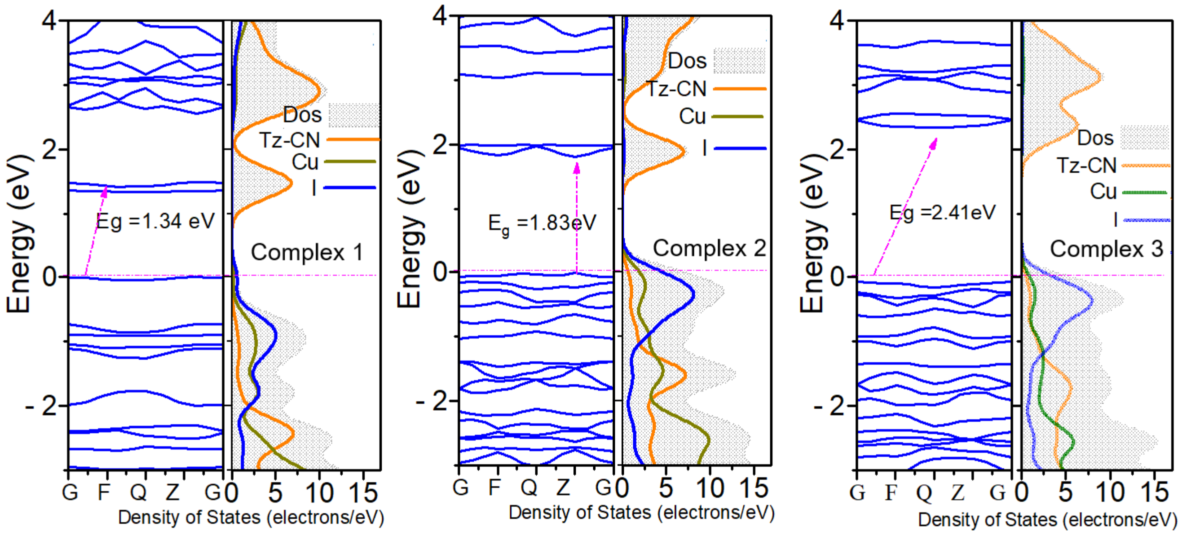

Figure S24. Band structure and density of states for complex **1** (**5CNtz**-CuI), complex **2** (**2CNtz**-CuI) and complex **3** (**4CNtz**-CuI). (DFT-MBD+U5.5)

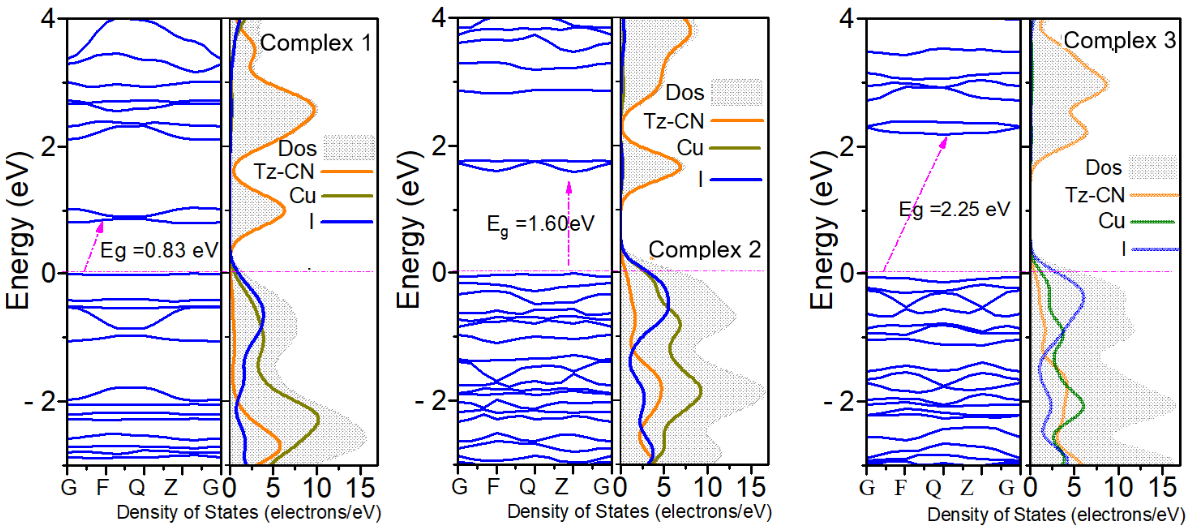

Figure S25. Band structure and density of states for complex **1** (**5CNtz**-CuI), complex **2** (**2CNtz**-CuI) and complex **3** (**4CNtz**-CuI). (DFT-MBD+U3.5)

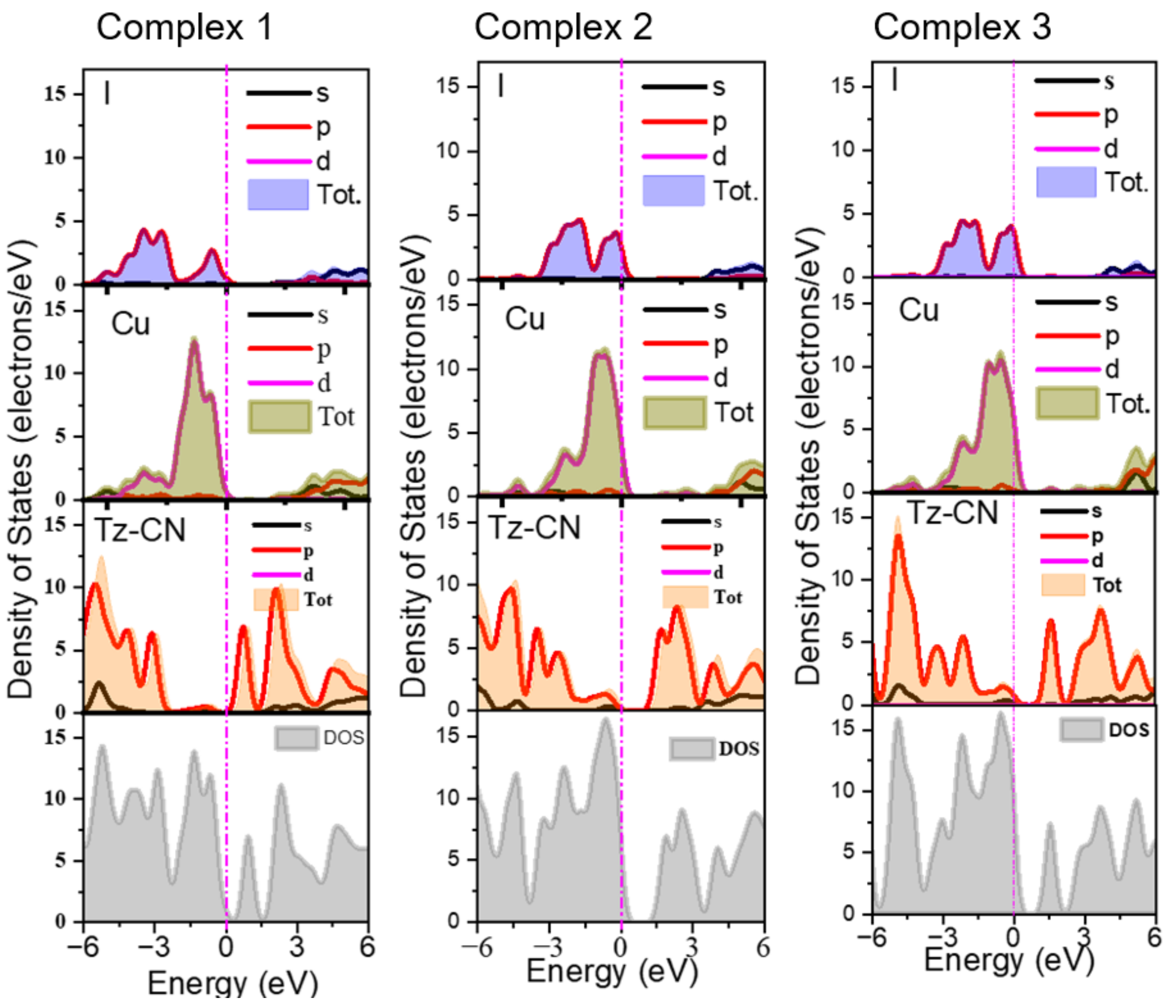

Figure S26. Partial density of states for complex **1** (**5CNtz**-CuI), complex **2** (**2CNtz**-CuI) and complex **3** (**4CNtz**-CuI) at DFT-MBD level of theory.

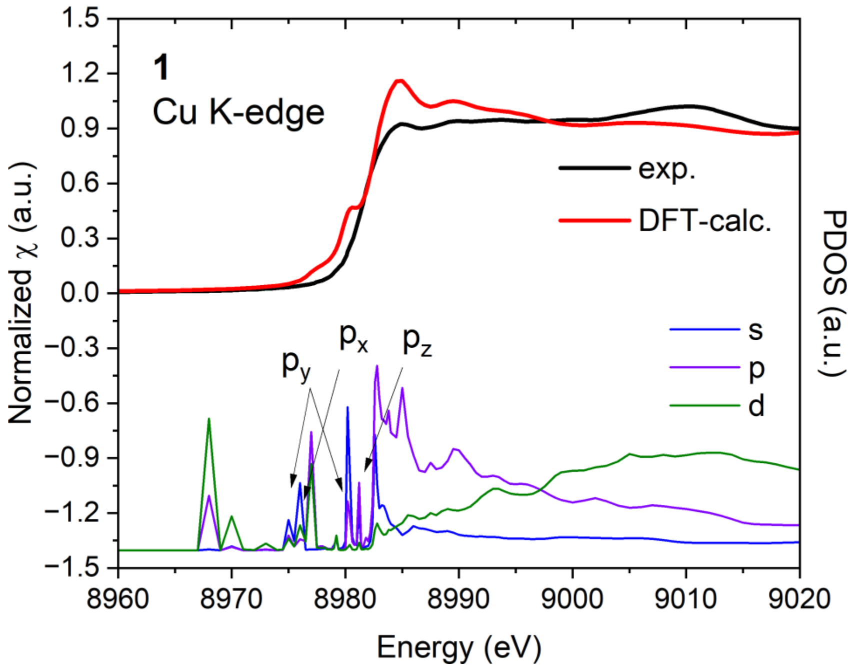

Figure S27. DFT-calculated XAS spectra and density for states compared with experimental data for complex **1**.

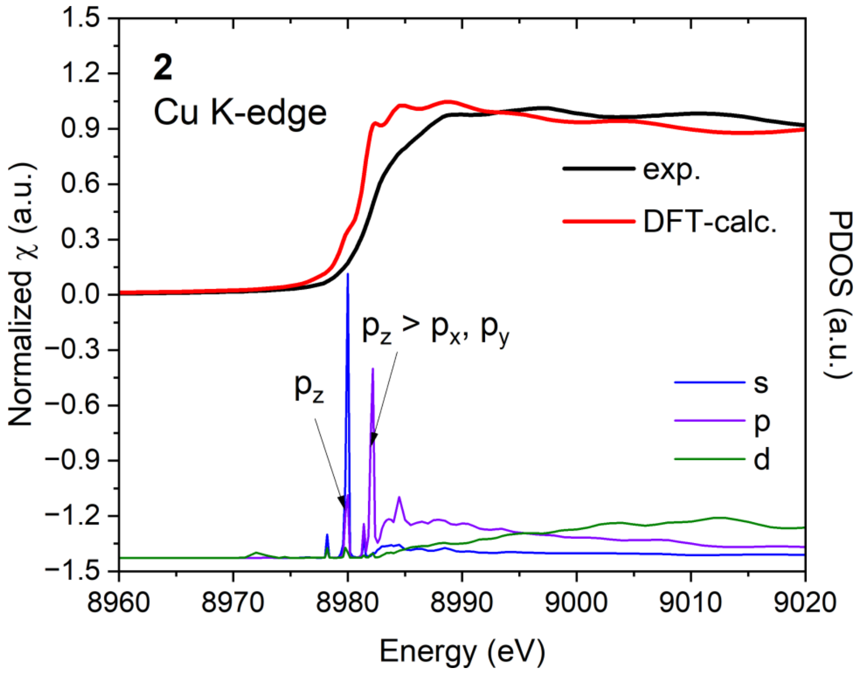

Figure S28. DFT-calculated XAS spectra and density for states compared with experimental data for the complex 2.

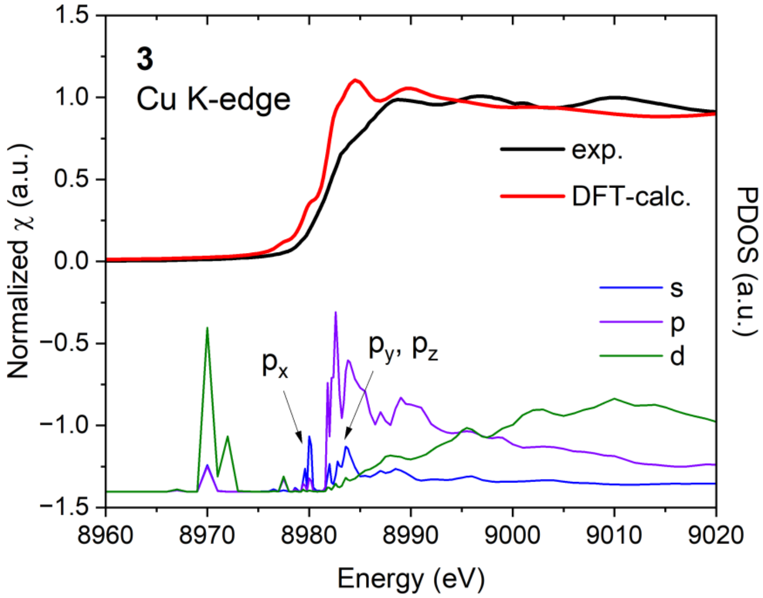

Figure S29. DFT-calculated XAS spectra and density for states compared with experimental data for complex **3**.

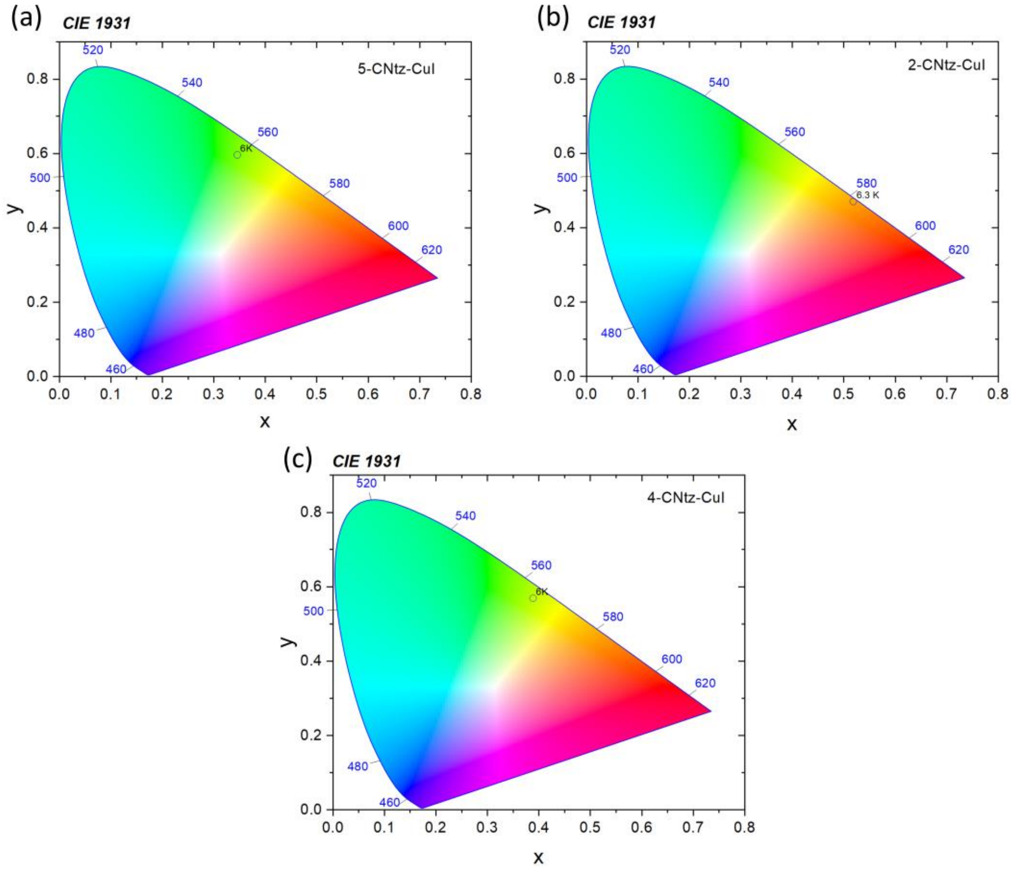

Figure S30. CIE1931 chromaticity diagrams calculated for emission spectra of **1** (a), **2** (b) and **3** (c) recorded at 6 K.

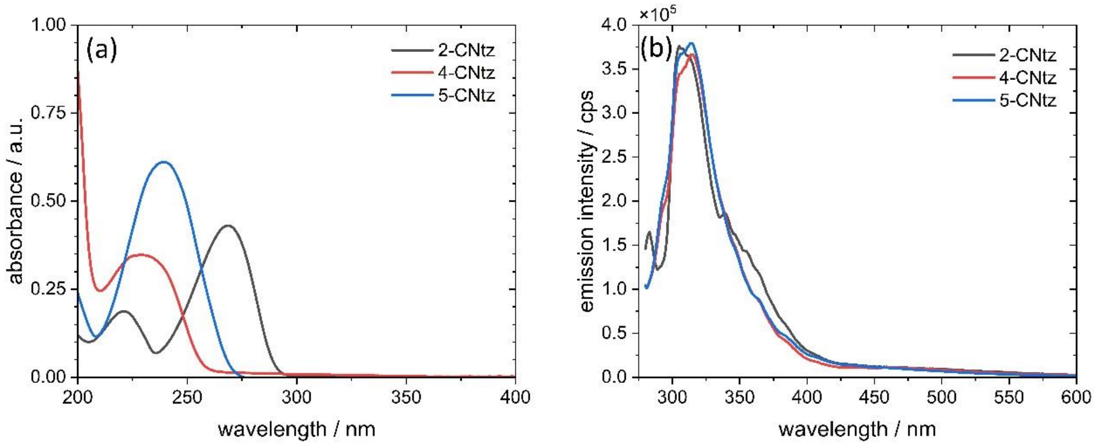

Figure S31. Absorption and emission spectra of cyanothiazole isomers in acetonitrile solutions. Emission was recorded with 275 nm excitation.

NMR Spectroscopy

We have measured the NMR spectra of cyanothiazoles and their CuI complexes in deuterated acetonitrile. Similar to UV-Vis spectroscopy, the spectra indicate dissociation of the ligands in acetonitrile. Only very small shifts in the proton and carbon signals are observed when comparing the spectra of cyanothiazoles and their complexes. Figure S32 illustrates the symbols for C and H atoms used in the NMR assignments. All relevant spectra are shown as figures S33-S44.

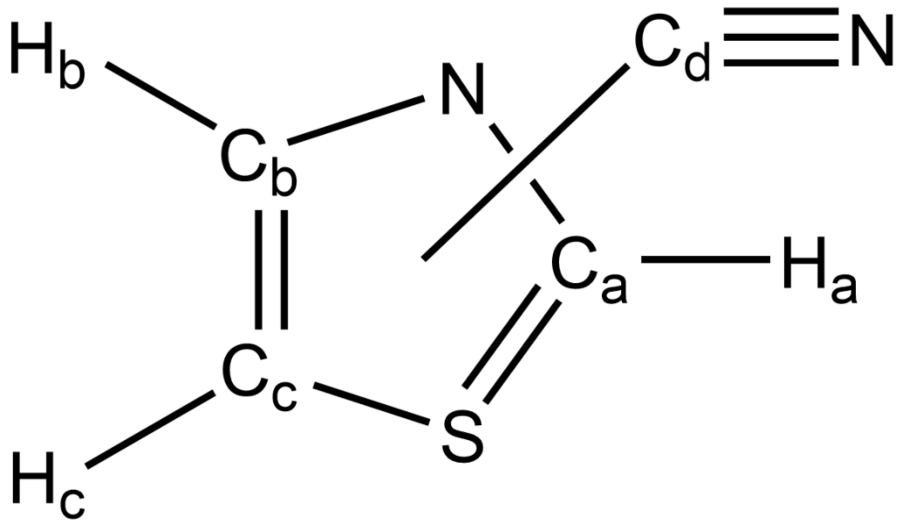

Figure S32. The symbols for C and H atoms used in the NMR assignments.

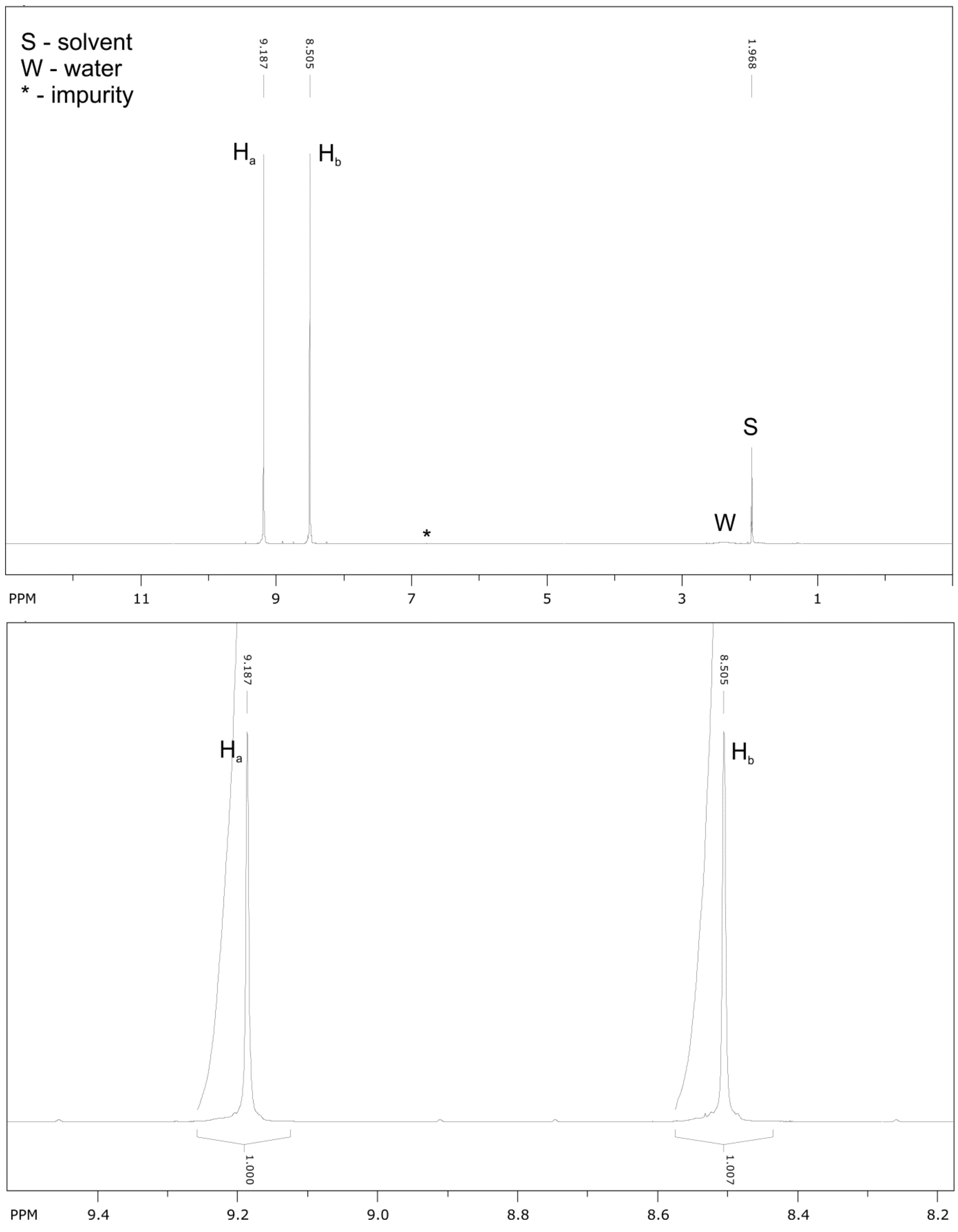

Figure S33 ^1H NMR spectra of **5CNtz** in CD_3CN-*d*$_3$.

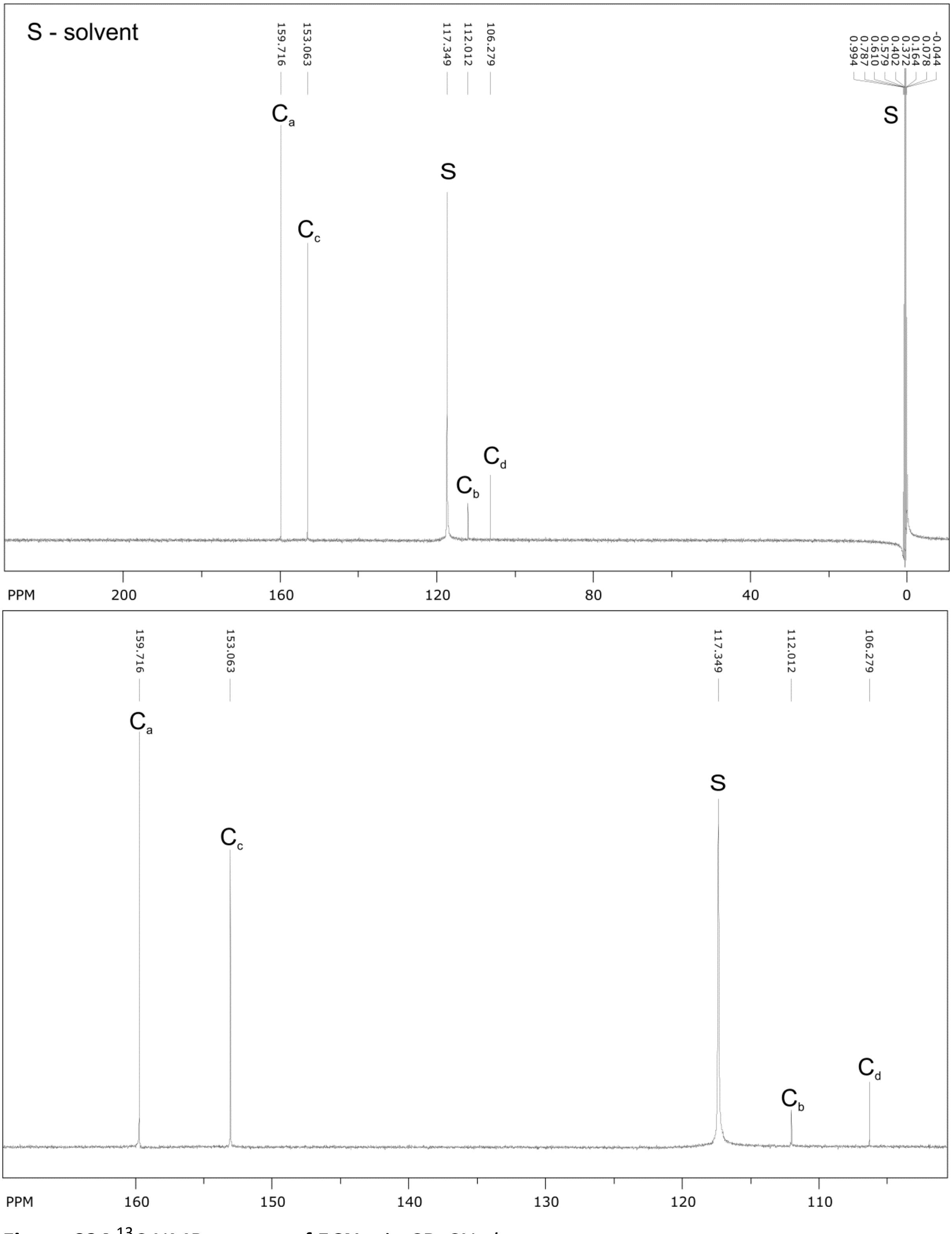

Figure S34 ^{13}C NMR spectra of **5CNtz** in CD_3CN-d_3.

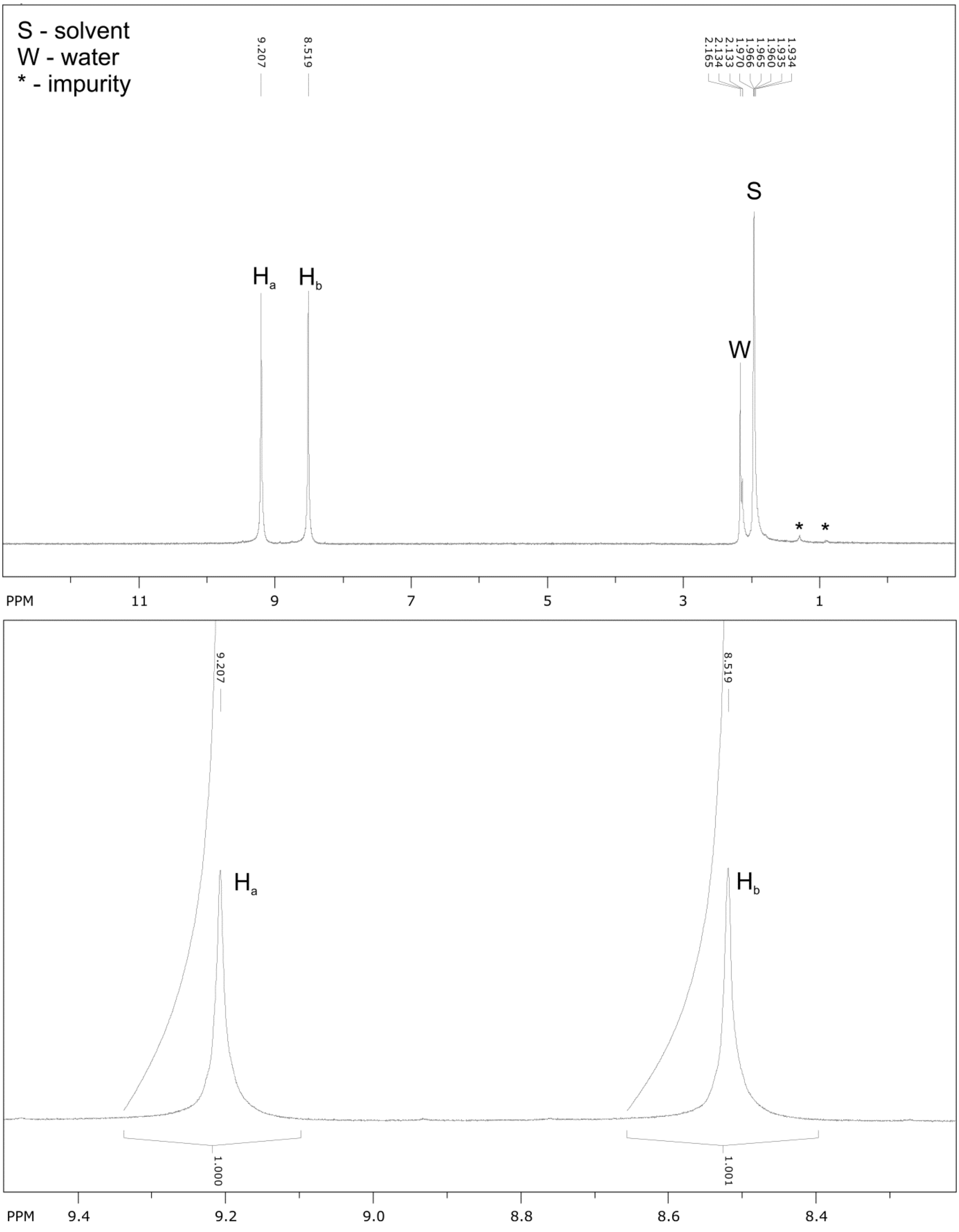

Figure S35 ^{1}H NMR spectra of Complex **1 [(5CNtz)CuI]$_\infty$** in CD_3CN-d_3.

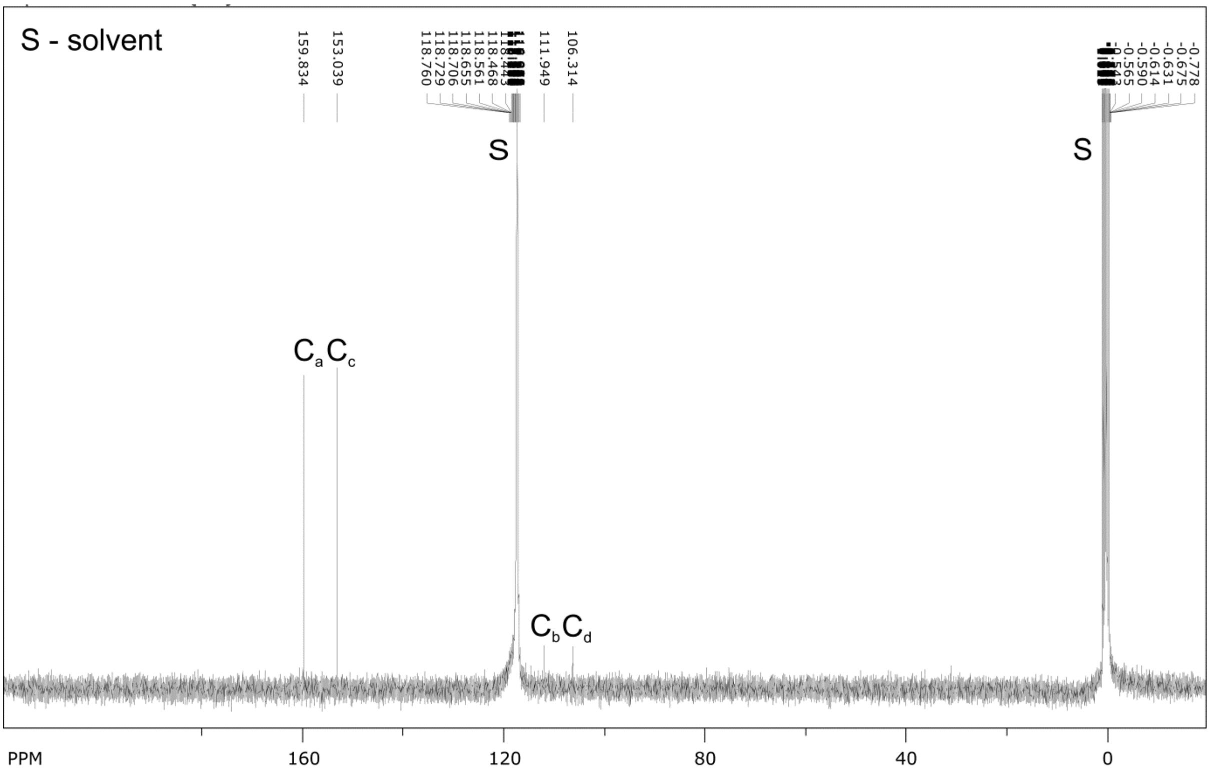

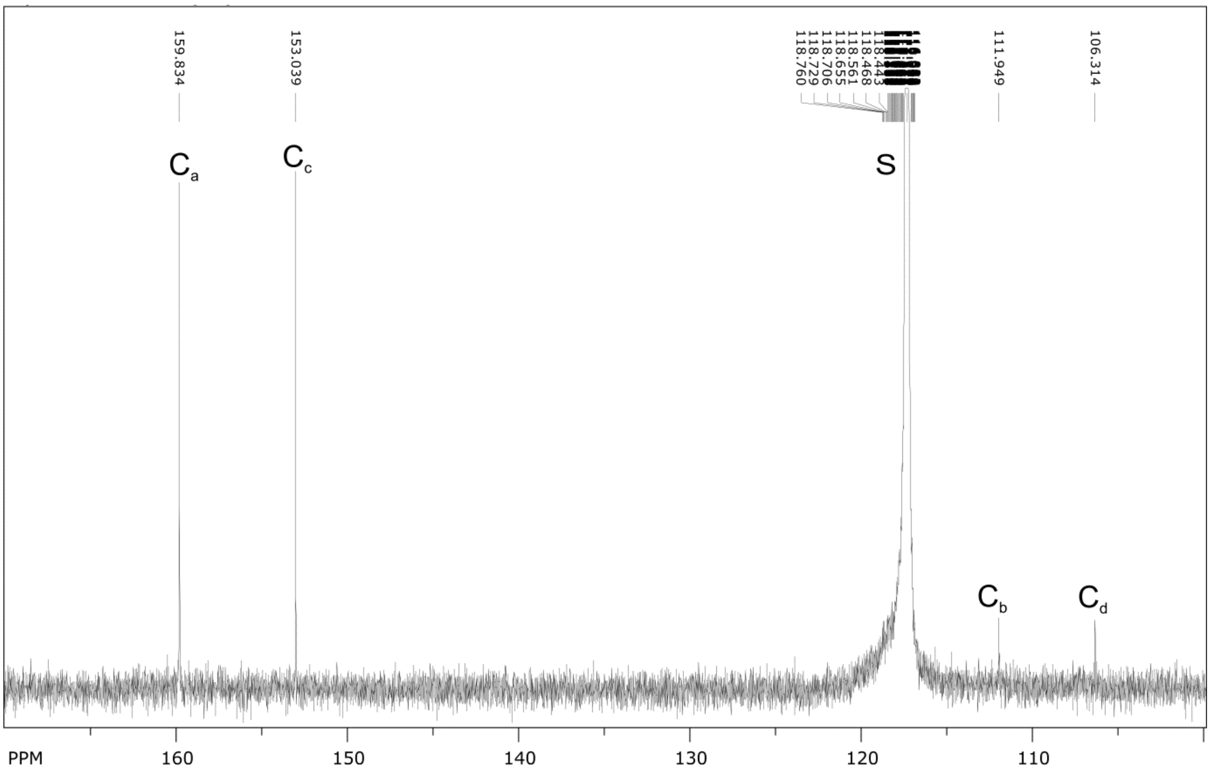

Figure S36 ^{13}C NMR spectra of Complex **1 [(5CNtz)CuI]$_\infty$** in CD_3CN-d_3.

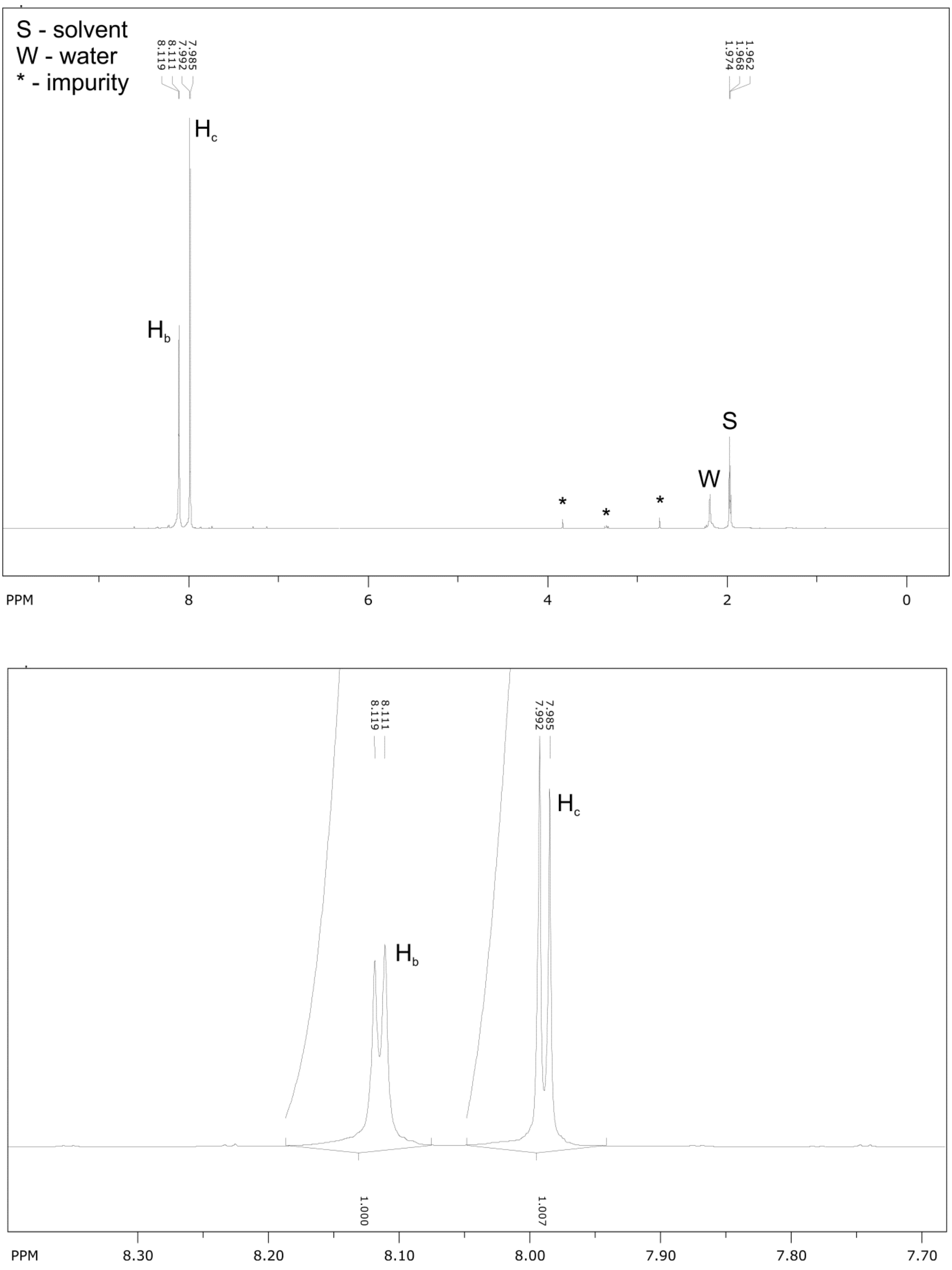

Figure S37 ^{1}H NMR spectra of **2CNtz** in CD_3CN-d_3.

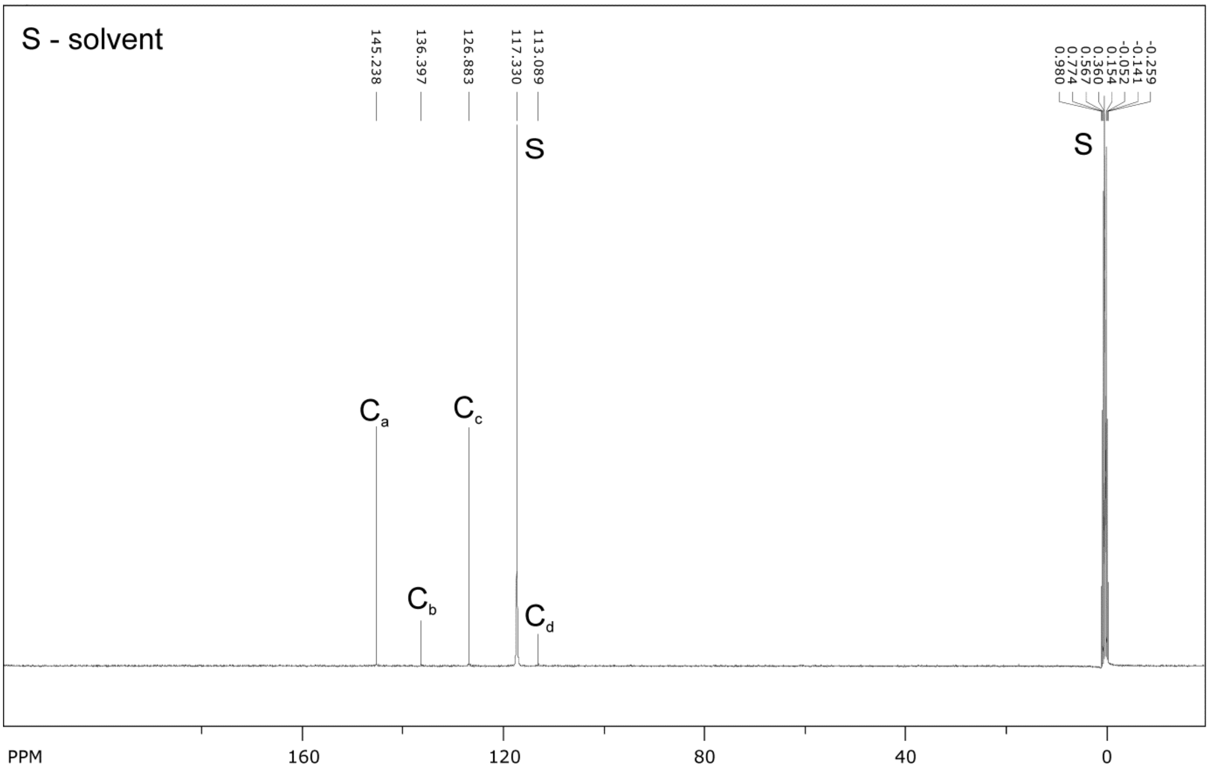

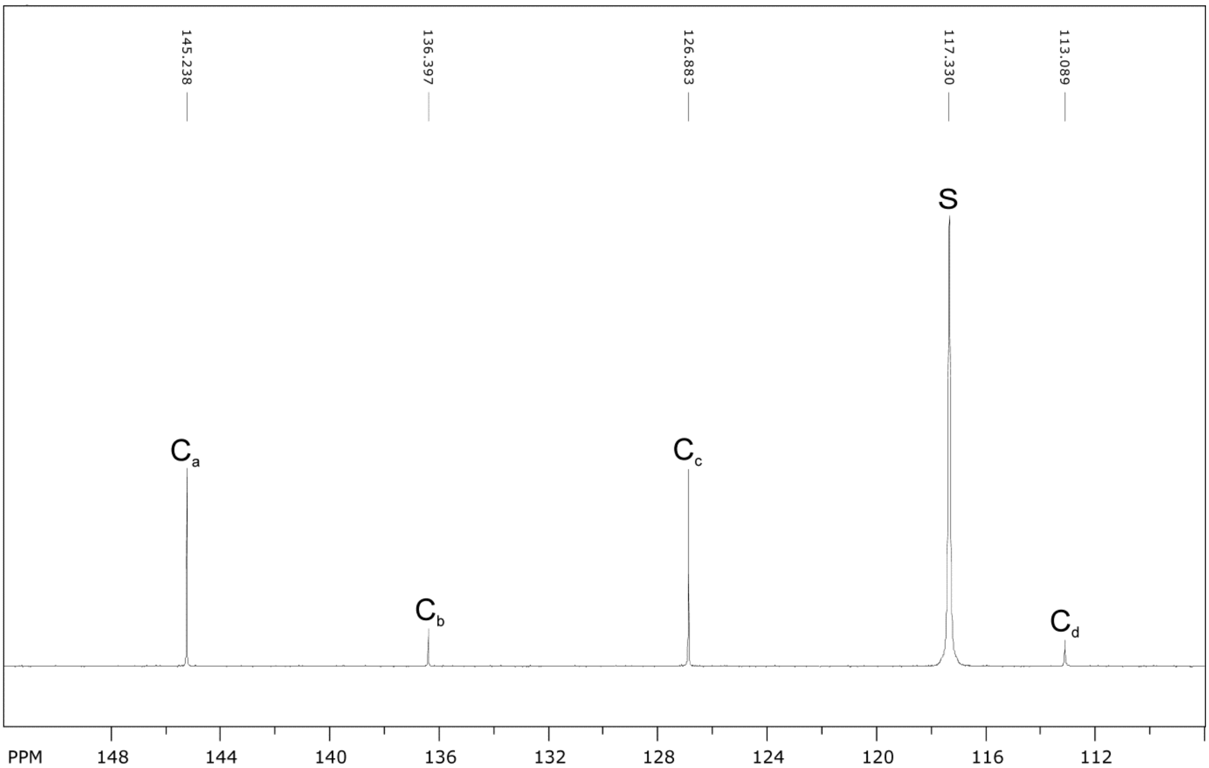

Figure S38 ^{13}C NMR spectra of **2CNtz** in CD_3CN-d_3.

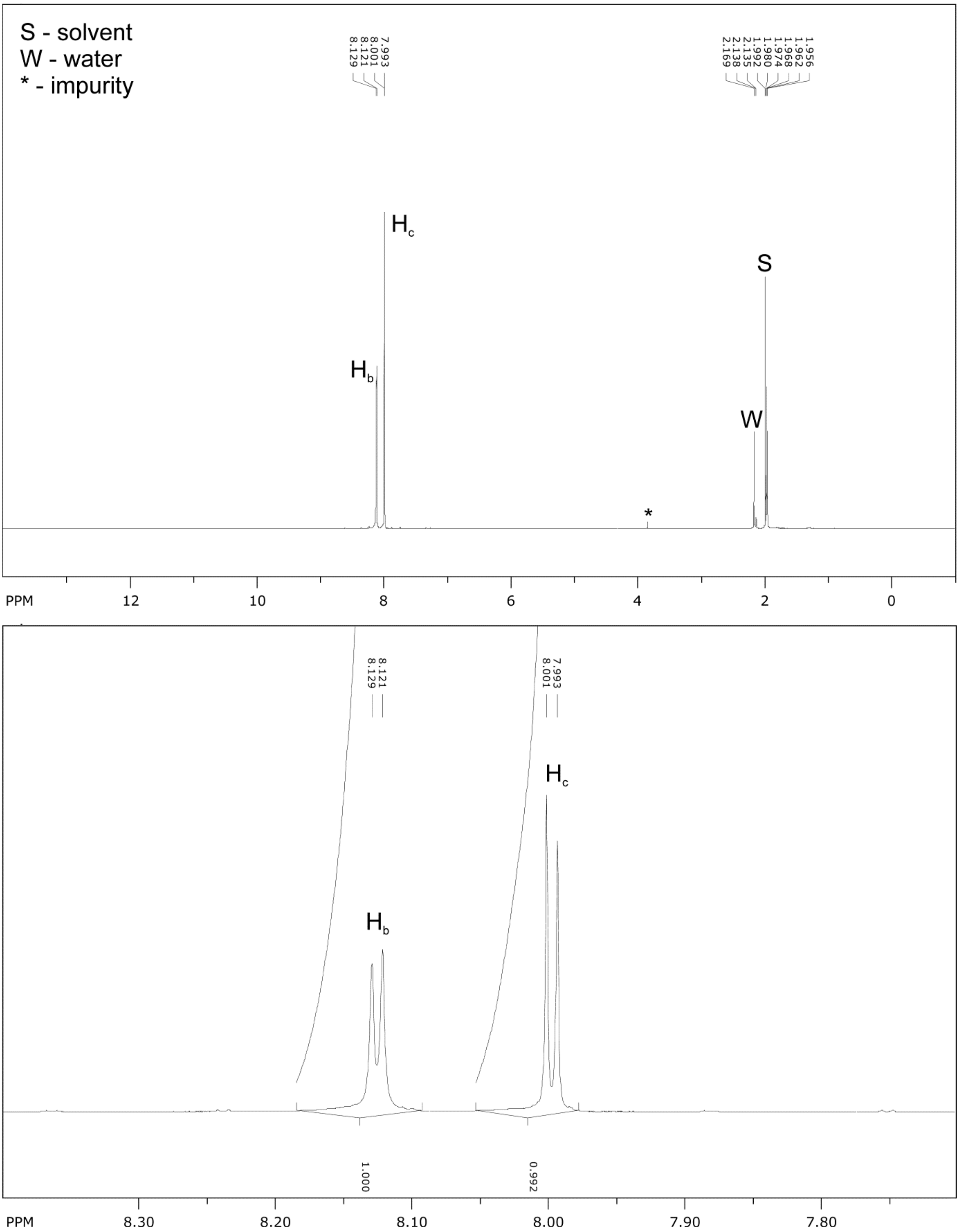

Figure S39 ^{1}H NMR spectra of Complex **2 [(2CNtz)CuI]$_\infty$** in CD_3CN-d_3.

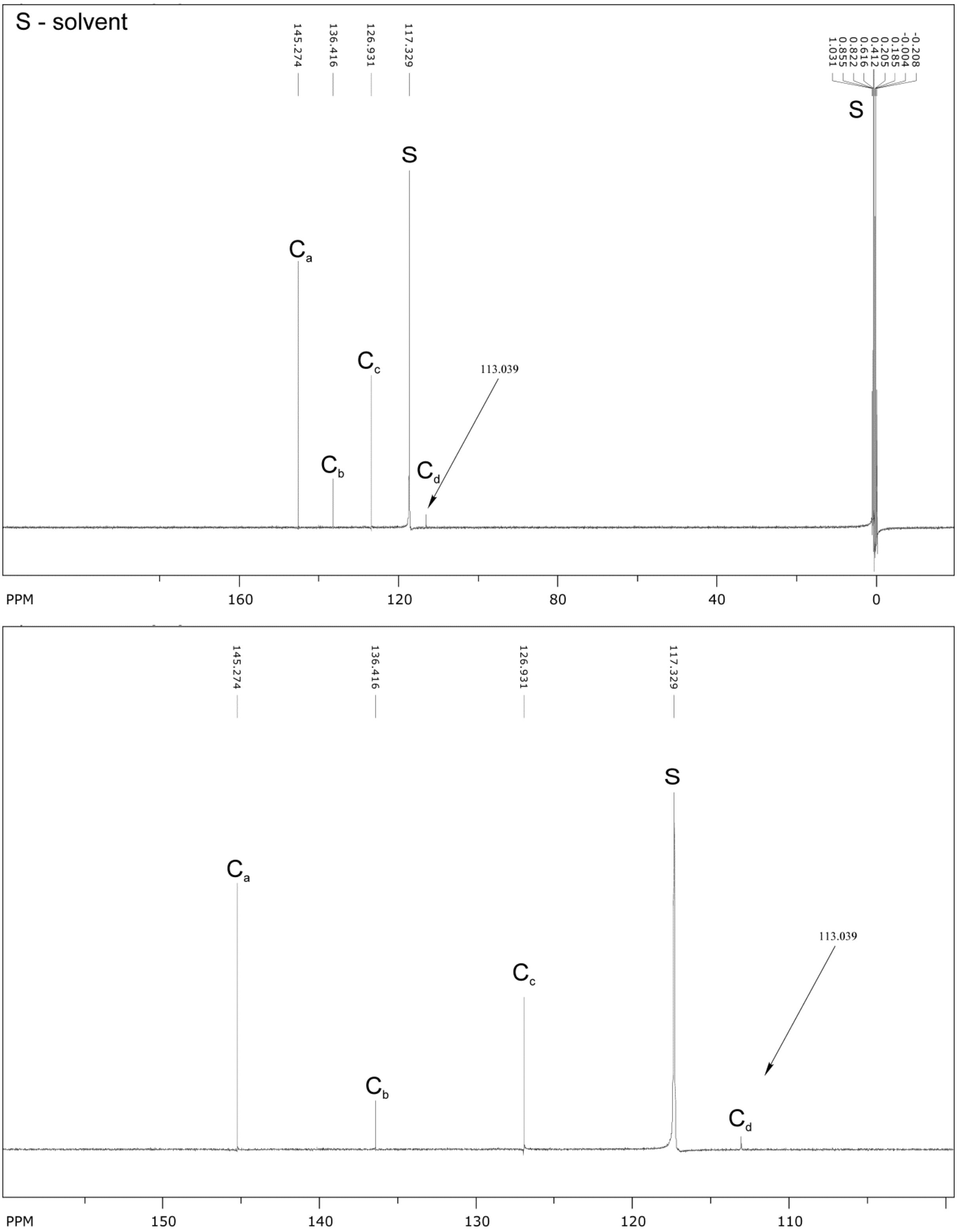

Figure S40 ^{13}C NMR spectra of Complex **2 [(2CNtz)CuI]∞** in CD_3CN-d_3.

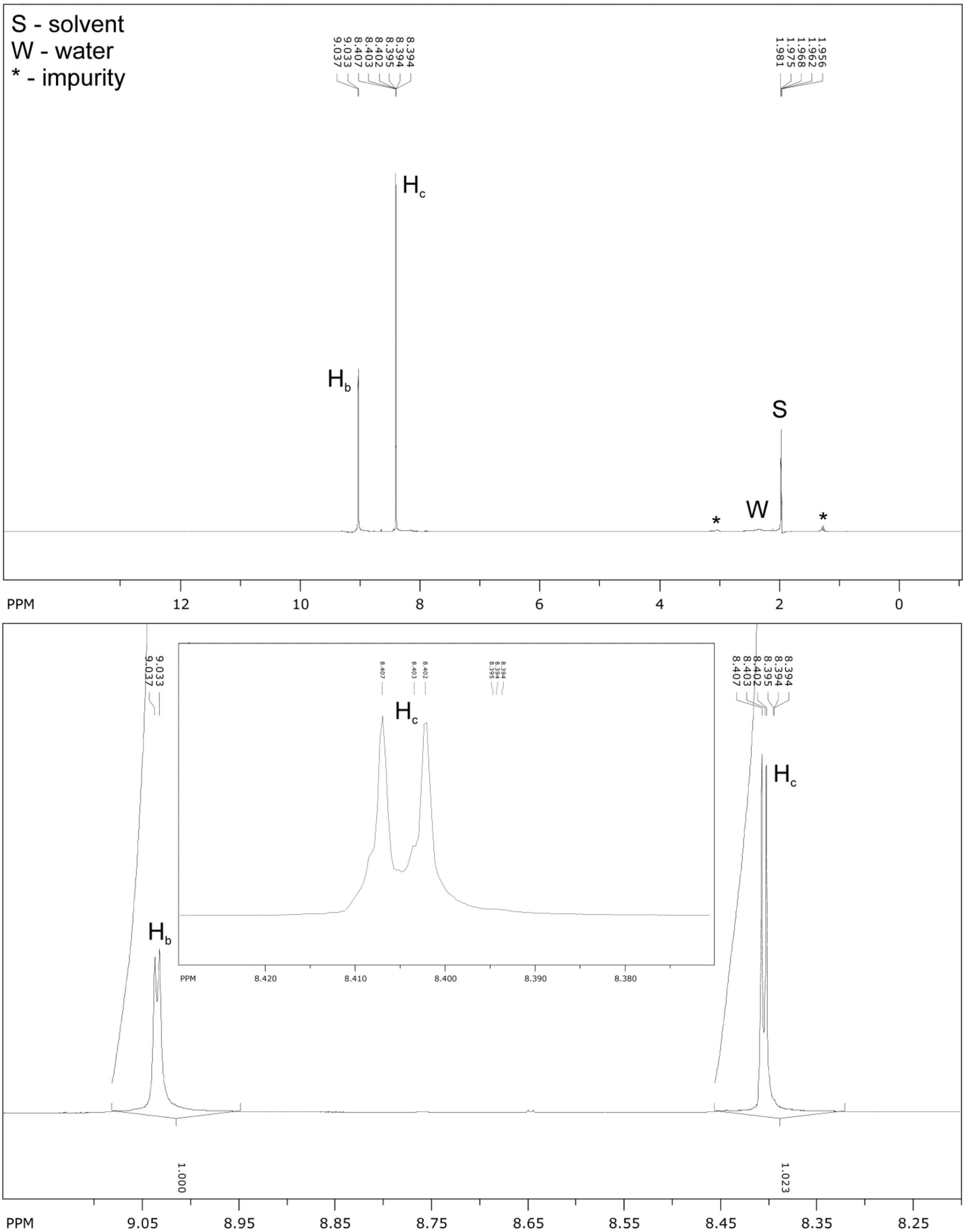

Figure S41 ^{1}H NMR spectra of **4CNtz** in CD_3CN-d_3.

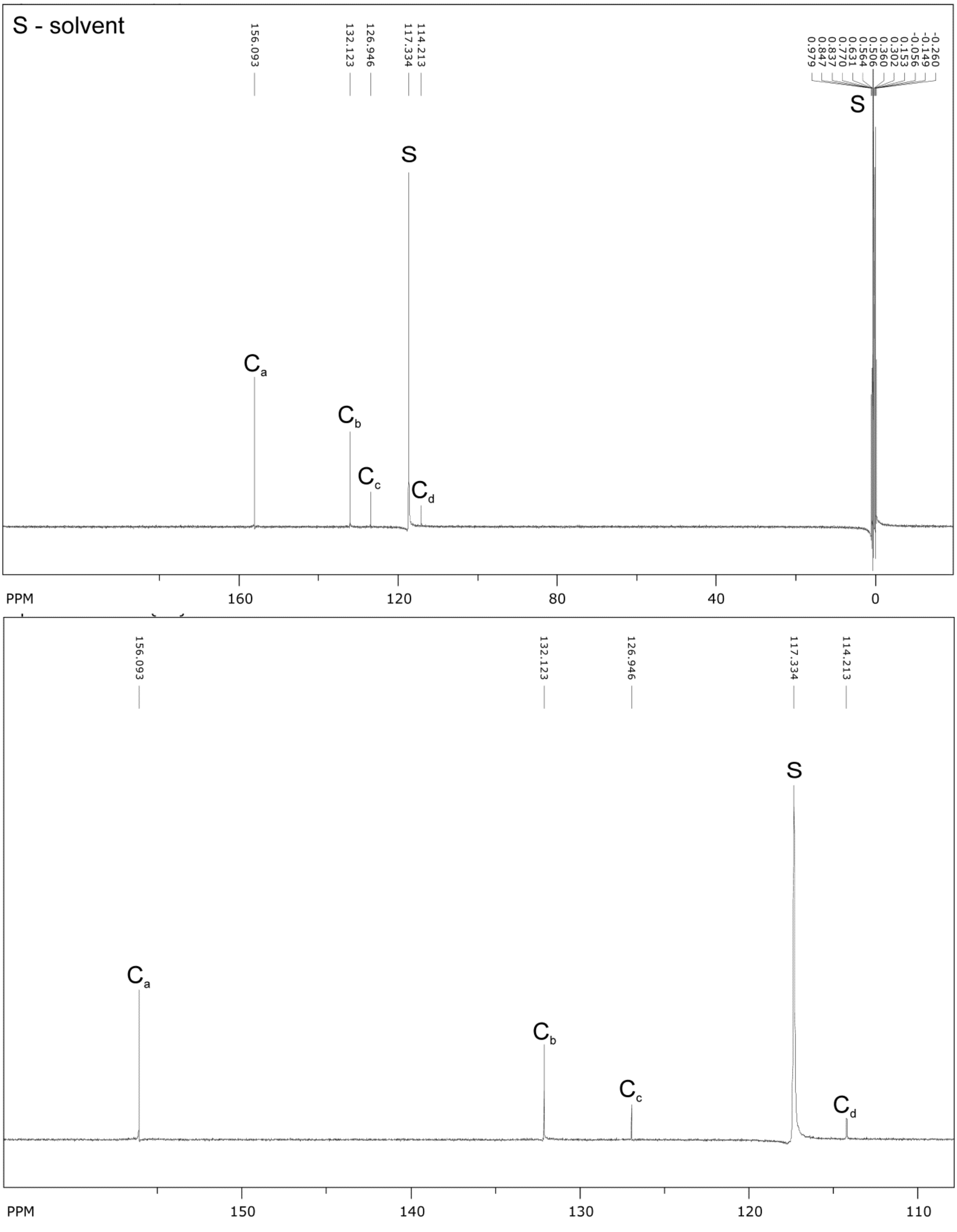

Figure S42 ^{13}C NMR spectra of **4CNtz** in CD_3CN-d_3.

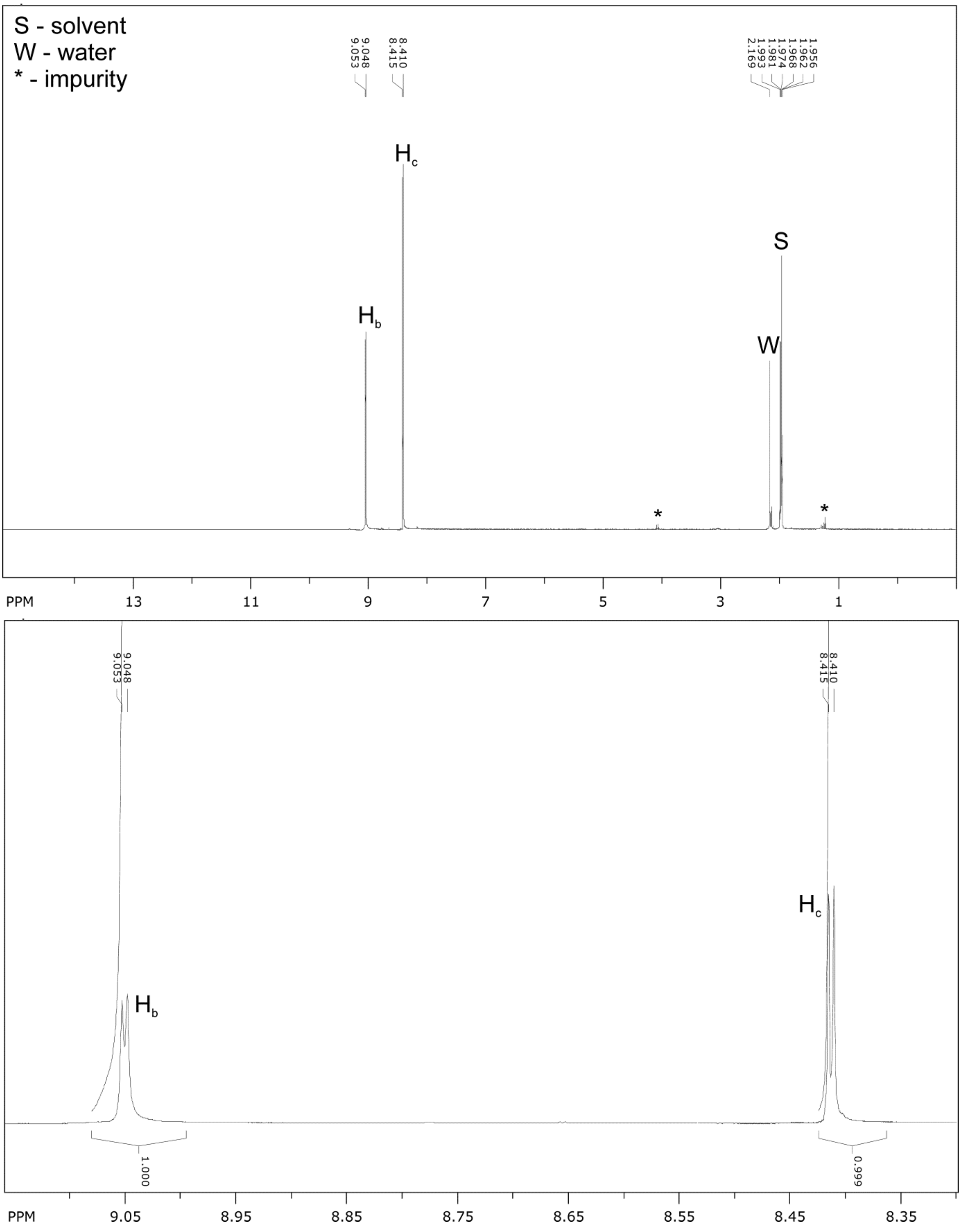

Figure S43 ^{1}H NMR spectra of Complex **3 [(4CNtz)CuI]$_{\infty}$** in CD_3CN-d_3.

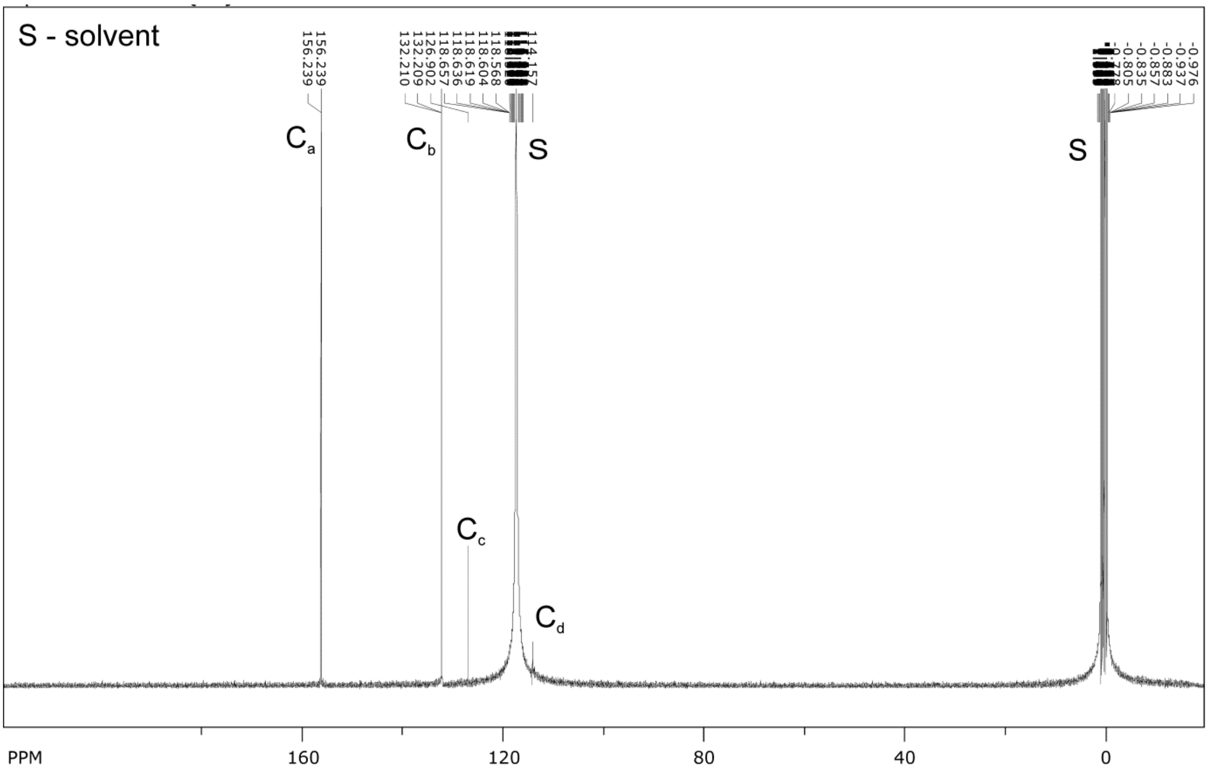

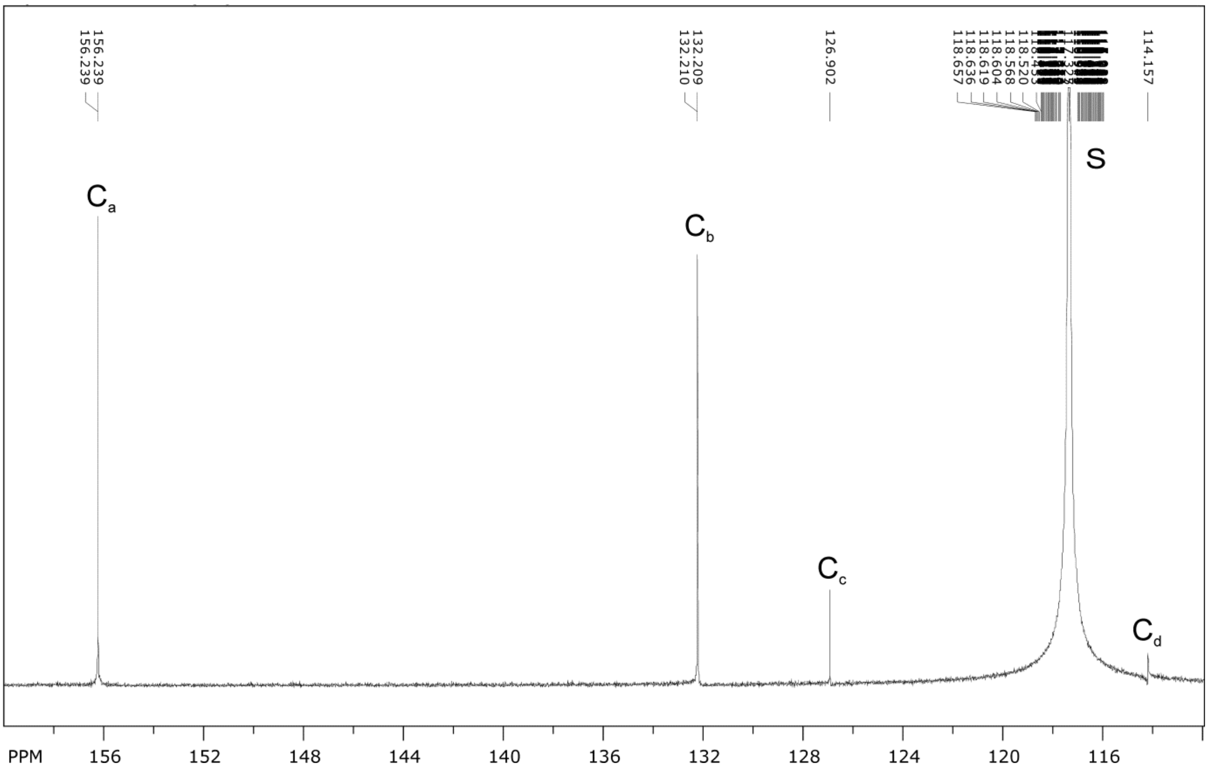

Figure S44 ^{13}C NMR spectra of Complex **3 [(4CNtz)CuI]$_\infty$** in CD_3CN-d_3.
